# Spt-Ada-Gcn5-Acetyltransferase (SAGA) Complex in Plants: Genome Wide Identification, Evolutionary Conservation and Functional Determination

**DOI:** 10.1371/journal.pone.0134709

**Published:** 2015-08-11

**Authors:** Rakesh Srivastava, Krishan Mohan Rai, Bindu Pandey, Sudhir P. Singh, Samir V. Sawant

**Affiliations:** 1 Plant Molecular Biology & Genetic Engineering Laboratory, Council of Scientific and Industrial Research, National Botanical Research Institute (CSIR-NBRI), Lucknow, Uttar Pradesh, India; 2 School of Biotechnology, Faculty of Science, Banaras Hindu University, Varanasi, Uttar Pradesh, India; 3 National Agri-Food Biotechnology Institute (DBT), Mohali, Panjab, India; National Institute of Plant Genome Research (NIPGR), INDIA

## Abstract

The recruitment of RNA polymerase II on a promoter is assisted by the assembly of basal transcriptional machinery in eukaryotes. The Spt-Ada-Gcn5-Acetyltransferase (SAGA) complex plays an important role in transcription regulation in eukaryotes. However, even in the advent of genome sequencing of various plants, SAGA complex has been poorly defined for their components and roles in plant development and physiological functions. Computational analysis of *Arabidopsis thaliana* and *Oryza sativa* genomes for SAGA complex resulted in the identification of 17 to 18 potential candidates for SAGA subunits. We have further classified the SAGA complex based on the conserved domains. Phylogenetic analysis revealed that the SAGA complex proteins are evolutionary conserved between plants, yeast and mammals. Functional annotation showed that they participate not only in chromatin remodeling and gene regulation, but also in different biological processes, which could be indirect and possibly mediated *via* the regulation of gene expression. The *in silico* expression analysis of the SAGA components in *Arabidopsis* and *O*. *sativa* clearly indicates that its components have a distinct expression profile at different developmental stages. The co-expression analysis of the SAGA components suggests that many of these subunits co-express at different developmental stages, during hormonal interaction and in response to stress conditions. Quantitative real-time PCR analysis of SAGA component genes further confirmed their expression in different plant tissues and stresses. The expression of representative salt, heat and light inducible genes were affected in mutant lines of SAGA subunits in *Arabidopsis*. Altogether, the present study reveals expedient evidences of involvement of the SAGA complex in plant gene regulation and stress responses.

## Introduction

The regulation of gene expression is accomplished by the coordinated action of multiple events to ensure a perfect synchrony of cellular activities from chromatin modification to mRNA formation [1–4]. Gene regulation in eukaryotes requires association of pre-initiation complex (PIC), transcription factors and activators at promoters [1, 5, 6]. One well-known mechanism for transcriptional activation suggests that activator proteins interact with promoter to recruit the components of transcriptional machineries and co-activators such as Transcription Factor II D (TFIID) complex, SAGA and mediator complexes [7–9]. The SAGA complex, a group of multi-protein complex, is important to induce the transcription of a subset of RNA polymerase II-dependent genes [10–12]. Indeed, the SAGA complex is a perfect archetype for multi-subunit histone modifying complexes and co-activator which regulates transcription by RNA polymerase II [13–15]. The first member of the SAGA complex family was isolated in budding yeast *Saccharomyces cerevisiae* [16]. The 1.8 megadalton *S*. *cerevisiae* SAGA complex is composed of 20 conserved proteins and contains different classes of transcriptional co-activator proteins such as SPT (Suppressor of Ty insertions), ADA (alteration/deficiency in activation), GCN5 (general control non-depressive), TAF (TBP-associated factors) proteins and DUBm (deubiquitylation module) [17]. These proteins are organized into different functional and structural sub-modules and thereby executing several cellular functions: nucleosomal histone acetyltransferase (HAT), histone deubiquitinylation, TATA-binding protein (TBP) binding and activator binding [10, 13, 18].

Interestingly, the SAGA complex is engaged in several transcription regulatory processes, for instance, facilitating recruitment of the RNA polymerase II, transcription elongation, promoting nucleosome eviction and replication-coupled nucleosome assembly [15, 19–21]. In addition, the SAGA complex is associated with nuclear export of transcribed mRNA, co-transcriptional spliceosome assembly and transcriptional silencing at telomere region [22–25]. Albeit, extensive evidences about the SAGA complex coding proteins in human, *S*. *cerevisiae* and other metazoan species are present, the knowledge about plants SAGA complex still needs to be elucidated. However, functions of few individual proteins of the SAGA complex are reported in plants, which have been shown to be involved in light signaling, stress response and histone modification [26–31].

The present study aims at determining the genes encoding subunits of the SAGA complex across the plant species, mainly in the *Arabidopsis thaliana* and *Oryza sativa*, using *in silico* approaches, and exploring their potential roles in the gene regulation. We have highlighted the functional annotation, co-expression profiles and possible interactome among different proteins of the plant SAGA complex. To better understand the plant SAGA complex, we investigated its roles in regulating the light and stress-induced gene expressions in *Arabidopsis*.

## Materials and Methods

### Plant materials and treatment methods

*Arabidopsis* ecotype Columbia-0 (Col-0) seeds were grown on 0.5 x Murashige and Skoog (MS) medium, kept for 48 hr at 4°C, and then shifted for growth at 20°C ± 1 underneath white light (16 hr light/ 8 hr dark at 100–120 μmol·m^−2^·s^−1^). For the stress treatments, three-week-old *Arabidopsis* excised leaves were used. Excised leaves were placed either in 0.5 x MS medium (mock treatment) or in 0.5 x MS medium with 150 mM NaCl solution for 24 hr. For the heat/high temperature treatment, excised *Arabidopsis* Col-0 leaves were kept in 0.5 x MS medium and transferred into a 37°C incubator for 2 hr, whereas the control samples were kept at 22°C. The following mutants were used in the present study: *gcn5‾* (Salk_030913c); *sgf29b‾* (Salk_128344c); *chr5‾* (Salk_020296c); *tra1a‾* (Salk_087015c); *taf12b‾* (Salk_132293c); *sgf11‾* (Salk_088988c) and *taf13‾* (Salk_024774c), which were acquired from the Arabidopsis Biological Resource Center [32].

### Plant’s genome database search for identification of SAGA complex

National Centre of Biotechnology Information (NCBI); TAIR (The *Arabidopsis* Information Resource) and RAP (Rice Genome Annotation Project) databases were used for the screening of the SAGA complex in *Arabidopsis*, *O*. *sativa* and other plant genomes. Protein sequences of *S*. *cerevisiae* and human SAGA complex components (Table 1, S1 and S2 Tables) were used as queries to execute a BLASTP program against the protein sequences of *Arabidopsis* and *O*. *sativa*.

**Table 1:** SAGA complex classification in *Arabidopsis* and *O*. *Sativa*.

SAGA Subunits	Yeast	Human	*Arabidopsis thaliana*		*Orzya sativa*		Functions	
			Name	Locus	Name	Locus	In plants	Ref.
**ADAs**	Ada1	ADA1	AtADA1a	At2g14850	OsADA1a	Os12g39090	-	
			AtADA1b	At5g67410	OsADA1b	Os03g55450	-	
	Ada2	ADA2b	AtADA2b	At4g16420	OsADA2b	Os03g53960	Response to auxin and cytokinin; Pleiotropic effects in development; Abiotic stress	[28, 99]
	Ada3	ADA3	AtADA3	At4g29790	OsADA3	Os05g28300	-	
	Gcn5 (Ada4)	GCN5	AtGCN5	At3g54610	OsGCN5	Os10g28040	HAT activity; Pleiotropic effects in development; Abiotic stress	[28, 45, 99]
**DUBm**	Ubp8	USP22	AtUBP22	At5g10790	OsUBP22	Os04g55360	-	
	Sgf11	ATXN7L3	AtSGF11	At5g58575	OsSGF11	Os05g28370	-	
	Sus1	ENY2	AtENY2	At3g27100	OsSUS1	Os01g69110	-	
	Sgf73	ATXN7	AtSGF73	ND	OsSGF73	ND	-	
**SPT**	Spt3	SPT3	AtSPT3	At1g02680	OsSPT3	Os01g23630	Seed development	[100]
	Spt7	STAF65/ SUPT7L	AtSPT7	At1g32750	OsSPT7	Os06g43790	-	
	Spt8	ND	AtSPT8	ND	OsSPT8	ND	-	
	Spt20 (Ada5)	SPT20	AtSPT20	At1g72390	OsSPT20	Os01g02860	Photoperiodic flowering regulation	[101, 102]
**TAFs**	Taf5	TAF5L	AtTAF5	At5g25150	OsTAF5	Os06g44030	Plant viability; Male gametogenesis; Pollen tube development	[90]
	Taf6	TAF6L	AtTAF6	At1g04950	OsTAF6	Os01g32750	Plant viability; Pollen tube growth	[83]
			AtTAF6b	At1g54360			-	
	Taf9	TAF9	AtTAF9	At1g54140	OsTAF9	Os03g29470	-	
		TAF9b			OsTAF9b	Os07g42150	-	
	Taf10	TAF10	AtTAF10	At4g31720	OsTAF10	Os09g26180	Salt tolerance during seed germination	[103]
	Taf12	TAF12	AtTAF12	At3g10070	OsTAF12	Os01g63940	-	
			AtTAF12b	At1g17440	OsTAF12b	Os01g62820	Negative response to ethylene and cytokinin signaling	[104, 105]
**Other Subunits**	Chd1	ND	AtCHR5	At2g13370	OsCHD1	OsJ_25446	Embryo development; Seed maturation	[106]
	Sgf29	STAF36	AtSGF29a	At3g27460	OsSGF29	Os12g19350	Flowering initiation, Auxiliary role in salt stress	[61]
			AtSGF29b	At5g40550			-	
	Tra1	TRRAP	AtTRA1a	At2g17930	OsTRA1	Os07g45064	-	
			AtTRA1b	At4g36080			-	

ND: Not detected.

### Alignment and phylogenetic analysis

Clustal-X version 1.83 software program was used for multiple sequence alignment of the protein sequences [33]. The aligned sequences were further used as input to create phylogenetic trees with the Neighbor-Joining method using a Jones-Taylor-Thornton (JTT) model. Bootstrapping was performed, involving 1000 replicates, to represent the evolutionary history of the group analyzed. The evolutionary distance was computed in MEGA 6.06 version [34].

### Domain analysis and chromosomal localization

The domain analysis was performed by CDD (Conserved Domain Database) and Pfam (protein families database) with an *e*-value **≤** 1.0. Chromosome Map Tool database was used to define the position of the SAGA complex genes on *Arabidopsis* chromosomes [35]. "Paralogous in *Arabidopsis*" were used for determining the gene duplications and their existence of duplicated segments on chromosome with parameters set to a threshold above 6 per block for paired proteins [36].

### Conserved motif analysis

The cis-regulatory elements/motifs were analyzed in 1000 bp upstream from the transcription start site (TSS) by using web based database Plant cis-acting regulatory DNA elements (PLACE) and Plant Cis-Acting Regulatory Elements (PlantCARE) databases and portals [37, 38].

### *In silico* microarray expression and protein interactome analysis

Microarray experiments data from Genevestigator database and analysis toolbox were employed to determine the gene expression profile of *Arabidopsis* and *O*. *sativa* SAGA complex genes in different tissue [39]. The cDNA signatures from Massively Parallel Signature Sequencing (MPSS) were used to count the number of corresponding mRNA molecules produced by each gene of *Arabidopsis* and *O*. *sativa* SAGA complex [40]. A protein-protein interaction network, for the prediction of functional associations within SAGA complex proteins, was prepared using the STRING database with a confidence threshold score of 0.6. [41]. The network was showed in the ‘evidence’ view, whereby lines linking proteins signify the category of evidence used in anticipating the association or interaction.

### Functional annotation and co-expression analysis

Functional annotation and Gene Ontology analysis were performed from TAIR and agriGO [35, 42]. Co-expression analysis for gene pairs and co-expressed gene network analysis for each SAGA gene was acquired from ATTED-II (The *Arabidopsis* trans-factor and cis-element prediction database) version c4.1 [43].

### RNA extraction and Real-time PCR analysis

Total RNA was extracted from the flowers, leaves, roots, seedlings, stems and siliques as well as from treated leaves by Sigma’s Spectrum plant total RNA isolation kit. The integrity of RNA, after DNase I treatment, was confirmed by agarose gel electrophoresis. Two microgram of total RNA was used as a template for first-strand cDNA synthesis using the Superscript-II RT kit (Invitrogen). Real-time PCR (qRT-PCR) gene expression analysis was performed and detected by using an ABI’s 7500 Fast Real-time PCR machine [44]. Gene specific forward and reverse primers were designed by using ABI’s-Primer express v2.0 software (S3 Table). The transcripts were normalized using Ubiquitin-10 (*Ubq10*, At4g05320) transcripts that work as internal control. The relative expression level of target genes was analysed by ΔΔCt method.

## Results

### Identification and classification of SAGA complex subunits in plants

The SAGA complex is a multiple subunit protein complex and is highly conserved among human, *S*. *cerevisiae* and *Drosophila* [13, 17]. The putative SAGA genes were identified in *Arabidopsis* and *O*. *sativa* genomes using protein sequences of *S*. *cerevisiae* and human SAGA genes as queries against the protein databases of *Arabidopsis* and *O*. *sativa* (NCBI, TAIR and RAP) (S1 and S2 Tables).

We identified four protein subunits in the ADA group of the SAGA complex, *viz*. ADA1, ADA2b, ADA3 and GCN5 (ADA4) (Table 1 and S2 Table). ADA2b (At4g16420) and GCN5 (At3g54610) have been previously studied in plants [7, 28, 30, 45, 46]; however, ADA1 and ADA3 proteins are yet to be characterized in plants. Two ADA1 proteins were identified each in *Arabidopsis* (At2g14850 and At5g67410) and *O*. *sativa* (Os12g39090 and Os03g55450) genome as homologs of *S*. *cerevisiae* and human ADA1 (Table 1 and S2 Table). Similarly, *Arabidopsis* (At4g29790) and *O*. *sativa* (Os01g73620) ADA3 were identified as homologs of *S*. *cerevisiae* and human ADA3 (Table 1 and S2 Table).

*S*. *cerevisiae* and human SAGA complex contain four proteins in DUBm group, which mainly participate in the histone deubiquitylation and mRNA export [47], however, we identified three out of four proteins in *Arabidopsis* and *O*. *sativa* (Table 1). *Arabidopsis* USP22 (At5g10790) and *O*. *sativa* UBP22 (Os04g55360) proteins were found as homologs of *S*. *cerevisiae* UBP8 and human USP22, respectively (S2 Table). *Arabidopsis* (At5g58575) and *O*. *sativa* (Os05g28370) SGF11 proteins were identified as homologs of human ATXN7L3 and *S*. *cerevisiae* SGF11 (Table 1 and S2 Table). Notably, *S*. *cerevisiae* SGF11 and human ATXN7L3 (*S*. *cerevisiae* SGF11 homolog) share low (15.3%) similarity between their protein sequences [48]. *Arabidopsis* (At3g27100) and *O*. *sativa* (Os01g69110) SUS1 showed significant homology with the corresponding *S*. *cerevisiae* SUS1 and human ENY2.

In *S*. *cerevisiae* and human, three to four proteins- SPT3, SPT7, SPT8 (not present in humans) and SPT20, have been reported in the SPT group of the SAGA complex (Table 1). Our study identified SPT3 and SPT20 proteins in *Arabidopsis* and *O*. *sativa*. Interestingly, the human SPT3 displays extensive sequence similarity to the histone fold motifs of TAF13 in its N-terminal region [49, 50]. We found conserved domain TAF13 in *Arabidopsis* At1g02680 and *O*. *sativa* Os01g23630 (Table 1 and S2 Table). The SPT20 domain was found to be conserved in *Arabidopsis* At1g72390 and *O*. *sativa* Os01g02860 proteins (Table 1 and S2 Table). In earlier studies, a low level of similarity was reported between SPT3 (30%) and SPT20 (32.5%) homologs of *S*. *cerevisiae* and human (S2 Table) [51, 52]. The SPT7 protein contains Bromo-domain, a motif found in several transcription factors and co-activators, which is responsible for the acetylation of histones and transcriptional activation [53–55]. In *Arabidopsis*, 29 Bromo-domain-containing proteins are reported [56]. The BLAST analysis suggested that *Arabidopsis* At1g32750 (e-value 3e-07) and *O*. *sativa* Os06g43790/Os02g38980 (e-value 2e-07/1e-07 and protein similarity 29 /25%, respectively) have the highest protein sequence similarity to *S*. *cerevisiae* SPT7 and particularly to its Bromo-domain region. However, human STAF65/SUPT7L (homolog of yeast Spt7) BLAST analysis revealed extremely low protein similarity and insignificant e-value of the search Spt7 homolog in *Arabidopsis* and rice genome. SPT8 protein of *S*. *cerevisiae* contains WD40 domain repeats and facilitates TBP interaction [8]. *Arabidopsis* and other plants encompass more than 200 putative WD40 domain containing proteins [57]. *Arabidopsis* At5g08390 and At5g23430 displayed protein similarity with corresponding *S*. *cerevisiae* SPT8. However, in plant genome, a large number of plant proteins comprising either Bromo-domain or WD40 domain, exhibited a substantial level of similarity with the Bromo-domain for SPT7 and the WD40 domain for SPT8, henceforth, further biochemical evidence is required to validate these subunits of the SAGA complex in the two plant species, *Arabidopsis* and *O*. *sativa*.

Interestingly, several TAFs subunits are shared by several complexes like TFIID, SAGA, SLIK (SAGA-like complex), and STAGA (SAGA altered, SPT8 absent) as earlier reported in *S*. *cerevisiae* and human [58]. Lago et al., 2004 explained about different TAFs and their conserved domain structures in *Arabidopsis* [59]. The TAF proteins in the SAGA complex include- TAF5, TAF6, TAF9, TAF10 and TAF12. However, our genome-wide similarity search analysis identified two candidate proteins representing TAF12 in *O*. *sativa* (Table 1), unlike only one protein reported previously [59].

Apart from these four groups, some other components also present in the SAGA complex, such as CHD1 (chromo-domain helicase DNA binding protein 1), TRA1 (Transcription-associated protein 1) and SGF29 (SAGA-associated factor 29) (Table 1). The CHD subfamily-I chromatin remodeling proteins, *S*. *cerevisiae* CHD1 and human CHD2, share 45% protein similarity (S2 Table) [60]. BLAST searches identified *Arabidopsis* CHR5 (At2g13370) and *O*. *sativa* CHD (OsJ_25446) as homologs of *S*. *cerevisiae* CHD1 and human CHD2 (Table 1 and S2 Table). Further, we also identified two proteins, At3g27460 and At5g40550 in *Arabidopsis* encoding SGF29, as reported recently [61] and one protein in *O*. *sativa* (Os12g19350) (Table 1 and S2 Table). TRA1 is a representative of a group of proteins that include DNA-dependent protein kinase catalytic subunit, ATM (Ataxia telangiectasia mutated) and TRRAP (transformation/transcription domain-associated protein), with the carboxyl-terminal regions related to phosphatidylinositol 3-kinases [62]. We identified two TRA1 protein orthologs in *Arabidopsis* (At2g17930 and At4g36080) and one in *O*. *sativa* (Os07g45064) with the corresponding *S*. *cerevisiae* TRA1 and human TRRAP (Table 1 and S2 Table). In some reports, RTG2 protein has been considered as a subunit of the SAGA complex [47], whereas sometimes it has been suggested as a variant of the SAGA complex, SLIK [1, 17, 63]. Further biochemical evidences are required to validate the presence of RTG2 in *Arabidopsis* and *O*. *sativa* SAGA/SLIK complex.

### Conserved domains in plant SAGA complex

**Fig 1:**
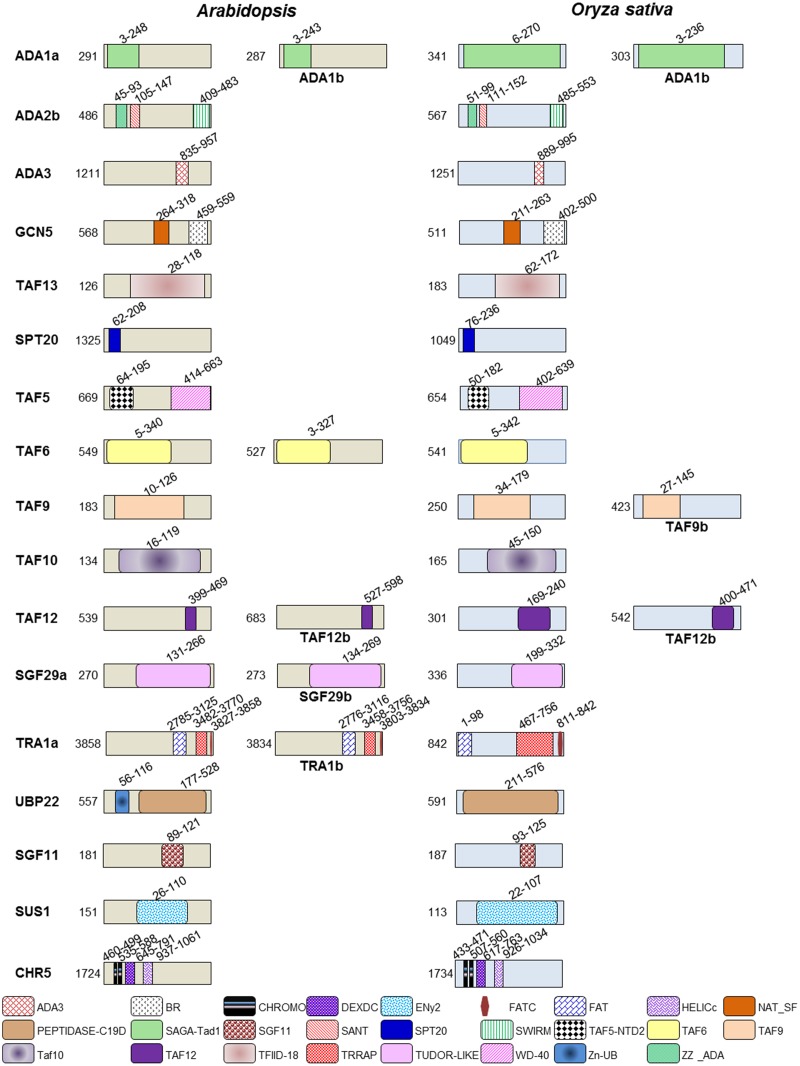
Domain organization of representative SAGA complex proteins in *Arabidopsis* and *Oryza sativa*. The positions of conserved domains which are typical for SAGA are shown. Domain abbreviations are: **BR:** Bromodomain; **NAT_SF:** N-Acyltransferase superfamily; **ZZ_ADA:** ZZ-type Zinc finger; **SANT:** SWI3, ADA2, N-CoR and TFIIIB DNA-binding domains; **SAGA-TAD1:** Transcriptional regulator of RNA pol II, SAGA, subunit; **ADA3:** ADA3 superfamily Histone acetyltransferases subunit 3; **TRRAP:** TRansformation/tRanscription domain-Associated Protein (TRRAP), pseudokinase domain; **FAT:** FRAP, ATM and TRRAP domain; **FATc:** FRAP, ATM, TRRAP C-terminal; **TAF5-NTD2:** N-terminal region of TATA Binding Protein (TBP) Associated Factor 5; **WD-40:** Trp-Asp (W-D) dipeptide 40 amino acid motifs; **TFIID-18:** Transcription factor II D 18kDa subunit; **PEPTIDASE C19D:** Peptidase C19 contains ubiquitinyl hydrolases; **Zn-UB:** Zn-finger in ubiquitin-hydrolases; **ENy2**: enhancer of yellow; **DEXDc:** DEAD-like helicases superfamily; **CHROMO:** Chromatin organization modifier (chromo) domain; **HELICc:** Helicase superfamily c-terminal domain; **SGF11:** SaGa associated Factor 11; **SWIRM:** SWI3, RSC8 and MOIRA. The number represents the amino acids in domain and protein.

The protein domains of *Arabidopsis* and *O*. *sativa* SAGA subunits, identified with the corresponding domains of *S*. *cerevisiae* and human SAGA subunits, is presented in Fig 1 and S1 Fig Intriguingly, numerous known structural features of protein domains in *Arabidopsis* and *O*. *sativa* SAGA complexes are common with *S*. *cerevisiae* and human SAGA complexes. These include HAT module, WD repeat domain, histone fold domains, DUBm, interaction and structural integrity protein domains. The protein similarity of each domain of the SAGA complex shows evolutionary conservation across the species (Table 2). The domains of plant SAGA components share moderate (30–50%) to the high (50% and above) similarity with their counterparts in *S*. *cerevisiae* and human excluding FAT-domain and chromo-domain (Table 2 and S1 Fig).

**Table 2:** Domain similarities in SAGA complex protein among *Arabidopsis*, human, *O*. *sativa* and *S*. *cerevisiae*.

SAGA Subunits	Domain	*Arabidopsis thaliana*			*Oryza sativa*		Human
		Human	*S*. *cerevisiae*	*O*. *sativa*	Human	*S*. *cerevisiae*	*S*. *cerevisiae*
**ADA1a**	SAGA-TAD1	33	40	57/60^a^	36	37	36
**ADA1b**	SAGA-TAD1	35	39	50/60^a^	36	41	-
**ADA2b**	ZZ_ADA2	47	64	89	57	69	51
	SANT	69	68	83	64	70	73
	SWIRM	63	60	76	58	54	50
**ADA3**	ADA3	45	46	51	39	44	45
**GCN5(ADA4)**	BROMO	72	63	83	72	59	63
	NAT_SF	71	80	89	72	81	74
**SPT3**	TAF13	57	60	74	50	50	63
**SPT20(ADA5)**	SPT20	48	33	62	42	34	34
**TAF5**	TAF5_NTD2	62	59	88	62	63	63
	WD40	50	51	94	49	51	66
**TAF6**	TAF6	62	58	88	61	60	57
**TAF6b**	TAF6	57	54	80	-	-	-
**TAF9**	TAF9	73	64	73	62	53	60
**TAF9b**	TAF9	-	-	68	63	54	-
**TAF10**	TAF10	69	55	87	66	56	53
**TAF12**	TAF12	54	53	62/59^a^	83	66	69
**TAF12b**	TAF12	76	68	83/85^a^	82	64	-
**TRA1a**	FAT	56	57	27	20	17	59
	TRRAP	59	58	93	56	57	57
	FATc	-	55	94	-	52	-
**TRA1b**	FAT	55	48	27	-	-	-
	TRRAP	58	57	91	-	-	-
	FATc	-	54	97	-	-	-
**SGF29a**	SGF29	49	48	82	52	47	36
**SGF29b**	SGF29	48	47	84	-	-	-
**UBP8**	PEPTIDASE-C19	60	49	74	60	47	52
**SGF11**	SGF11	70	76	91	69	73	61
**SUS1**	ENY2	76	76	89	81	81	100
**CHD1/CHR5**	CHROMO ^b^	19/35	18/34	72	21/33	14/32	18/16
	DEXDc	78	70	91	82	75	70
	HELICc	79	87	86	91	77	73

^a^For *Arabidopsis* first protein then second protein domain similarity percentage with *O*. *sativa* first protein and second protein domain given.

^b^For two domains of Chromo present in *S*. *cerevisiae* and human.

### Phylogenetic and evolutionary analysis of the SAGA complex family among different organisms

**Fig 2:**
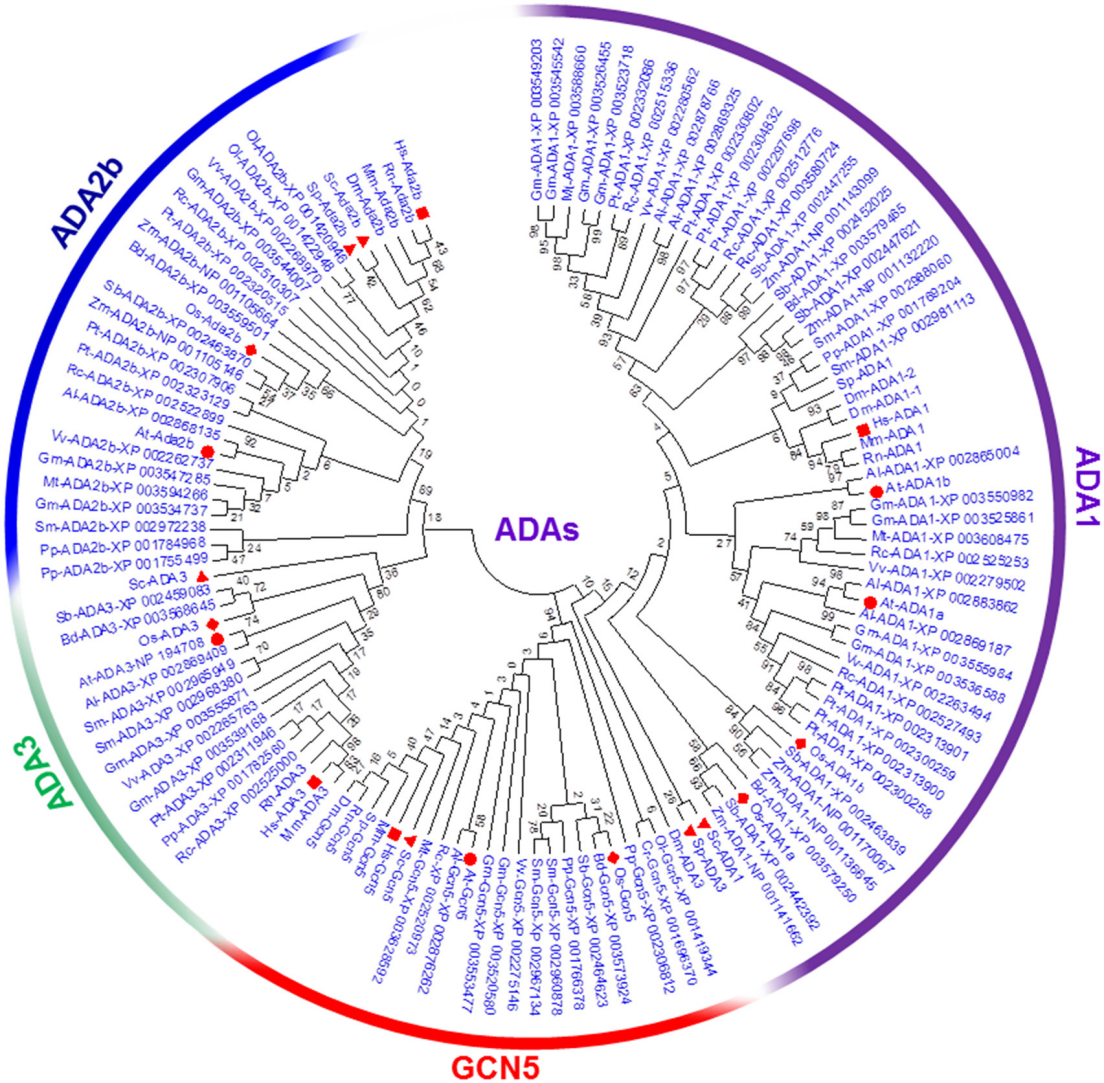
Phylogenetic relationship of the ADAs protein of the SAGA complex. ADAs protein sequences were used from At, *Arabidopsis thaliana* (red circle); Dm, *Drosophila melongaster*; Hs, *Homo sapiens* (red square); Os, *Oryza sativa* (red diamond shape); Mm, *Mus musculus*; Rs, *Rattus norvegicus;* Sc, *Saccharomyces cerevisiae* (red triangle); Sp, *Schizosaccharomyces pombe;* Zm, *Zea mays;* Rc, *Ricinus communis;* Pt, *Populus trichocarpa;* Vv, *Vitis vinifera;* Al, *Arabidopsis lyrta;* Mt, *Medicago truncatula;* Bd, *Brachypodium distachyon;* Sb, *Sorgum bicolor;* Sm, *Selaginella moellendorffii;* Pp, *Physcomitrella patens;* Cr, *Chlamydomonas reinhardtii* and Ol, *Ostreococcus lucimarinus*. Phylogeny reconstruction was analysed by neighbour-joining statistical method. Test of phylogeny was analysed by the bootstrap method (1,000 replicates).

**Fig 3:**
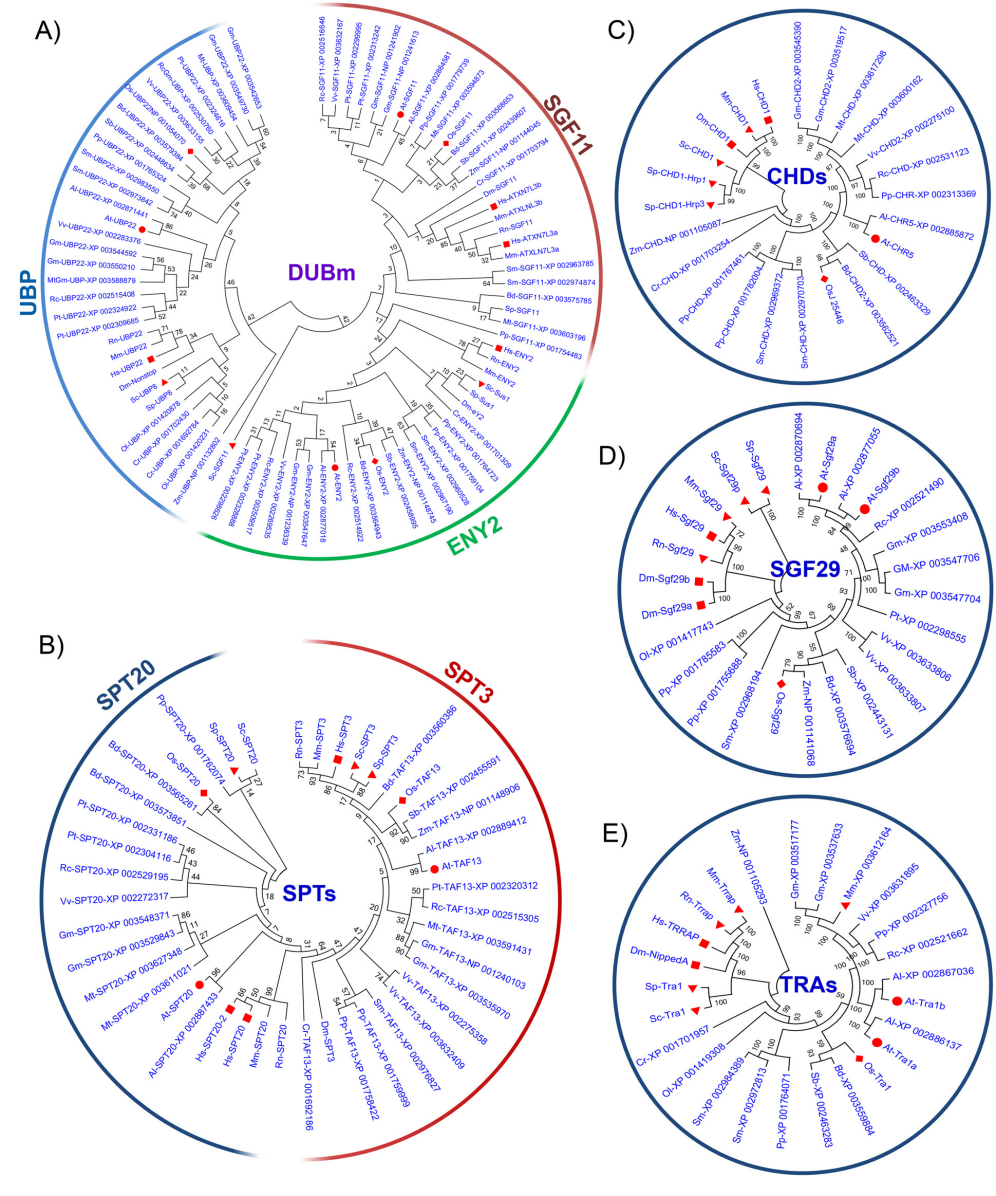
Phylogenetic relationship of DUBm, SPTs and other subunits of the SAGA complex. Different subunits of the SAGA complex protein sequences were used from At, *Arabidopsis thaliana* (red circle); Dm, *Drosophila melongaster*; Hs, *Homo sapiens* (red square); Os, *Oryza sativa* (red diamond shape); Mm, *Mus musculus*; Rs, *Rattus norvegicus;* Sc, *Saccharomyces cerevisiae* (red triangle); Sp, *Schizosaccharomyces pombe;* Zm, *Zea mays;* Rc, *Ricinus communis;* Pt, *Populus trichocarpa;* Vv, *Vitis vinifera;* Al, *Arabidopsis lyrta;* Mt, *Medicago truncatula;* Bd, *Brachypodium distachyon;* Sb, *Sorgum bicolor;* Sm, *Selaginella moellendorffii;* Pp, *Physcomitrella patens;* Cr, *Chlamydomonas reinhardtii* and Ol, *Ostreococcus lucimarinus*. Phylogeny reconstruction was analysed by neighbour-joining statistical method. Test of phylogeny was analysed by the bootstrap method (1,000 replicates). **(A)** DUBm protein; **(B)** SPTs protein; **(C)** CHDs protein; **(D)** SGF29 protein; **(E)** TRA1 protein.

To investigate the evolutionary association among *Arabidopsis* and *O*. *sativa* SAGA complex proteins, phylogenetic trees were made from the alignments of their full-length protein sequences together with SAGA complex proteins of mammals (*Homo sapiens*, *Mus musculus* and *Rattus norvegicus*), an arthropod (*Drosophila melanogaster*), fungi (*S*. *cerevisiae* and *Schizosaccharomyces pombe*), dicot plants (*A*. *thaliana*, *A*. *lyrata*, *Glycine max*, *Medicago truncatula*, *Populus trichocarpa*, *Ricinus communis*, and *Vitis vinifera*), monocot plants (*Brachypodium distachyon*, *O*. *sativa*, *Sorghum bicolor* and *Zea mays*), a tracheophyte (*Selaginella lepidophylla*), a bryophyte (*Physcomitrella patens*) and algae (*Chlamydomonas reinhardtii* and *Ostreococcus lucimarinus*). In order to evaluate the molecular evolutionary relationship and conservation among SAGA protein components in different organisms, we aligned the different SAGA subunits and constructed a phylogenetic tree for each group. The phylogenetic tree analysis inferred immense conservation among the SAGA protein domains in *S*. *cerevisiae*, mammals, *Arabidopsis*, *O*. *sativa*, algae, bryophyte and *Drosophila* (Figs 2 and 3; Table 3 and S2 Fig). In the case of ADA group, three clades were exhibited for each SAGA subunit. The first and second clades comprised GCN5 (ADA5) and ADA1 proteins, respectively, while the third clade further divided into sub groups- ADA3 and ADA2b (Fig 2). In the phylogenetic analysis of ADA proteins from various organisms fall in a similar clade, excluding SpADA3 and DmADA3, which were close to the ADA1 clade (Fig 2). Similar to ADA group, other groups made several clades based on their similar protein domain specific phylogenetic tree analyses (Fig 3). In the phylogenetic tree of TAFs group, CrTAF5 and OlTAF12 proteins were present in different clades (S2 Fig). The phylogenetic tree constructed from plant SAGA proteins revealed that these proteins diverge into monocots and dicots (Figs 2 and 3). Based on the phylogenetic tree analysis, most protein domains in the SAGA subunits were remained extremely conserved in *S*. *cerevisiae*, mammals, *Arabidopsis*, *O*. *sativa*, algae, lycopsida, bryophyte and *Drosophila* during the course of evolution (Table 3).

**Table 3:** Putative SAGA complex genes in higher and lower plant organisms.

Gene Symbol	Dicots						Monocots			Lycopsida	Bryophyte	Green alga	
	Gm^a^	Al^a^	Mt^a^	Pt^a^	Vv^a^	Rc^a^	Bd^a^	Sb^a^	Zm^a^	Sm^a^	Pp^a^	Cr^a^	Ol^a^
**SPT3**	NP_001240103	XP_002889412	XP_003591431	XP_002320312	XP_002275358	XP_002515305	XP_003560386	XP_002455591	NP_001148906	XP_002976827	XP_001759999	XP_001692186	XP_001418060
	XP_003535970	-	-	-	XP_003632409	-	-	-	-	-	XP_001758422	-	-
**SPT20**	XP_003529843	XP_002887433	XP_003627348	XP_002304116	XP_002272317	XP_002529195	XP_003573851	ND	ND	ND	XP_001762074	ND	ND
	XP_003548371	-	XP_003611021	XP_002331186	-	-	XP_003565261	-	-	-	-	-	-
**ADA1**	XP_003555984	XP_002883862	XP_003608475	XP_002330802	XP_002279502	XP_002527493	XP_003580724	XP_002442392	NP_001170067	XP_002988060	XP_001769204	ND	ND
	XP_003550982	XP_002878766	XP_003588660	XP_002332086	XP_002280562	XP_002525253	XP_003579465	XP_002447621	NP_001143099	XP_002981113	-	-	-
	XP_003549203	XP_002872330	-	XP_002313901	XP_002263494	XP_002515336	XP_003579250	XP_002447255	NP_001141662	-	-	-	-
	XP_003536588	XP_002869325	-	XP_002313900	-	XP_002512776	XP_003579249	XP_002452025	NP_001136645	-	-	-	-
	XP_003526455	XP_002869187	-	XP_002304832	-	-	-	XP_002463839	NP_001132220	-	-	-	-
	XP_003525861	XP_002865004	-	XP_002300259	-	-	-	-	-	-	-	-	-
	XP_003523718	-	-	XP_002300258	-	-	-	-	-	-	-	-	-
	XP_003545542	-	-	XP_002297698	-	-	-	-	-	-	-	-	-
**ADA2b**	XP_003534737	XP_002868135	XP_003594266	XP_002323129	XP_002262737	XP_002522899	XP_003559501	XP_002463870	NP_001105146	XP_002972238	XP_001755499	ND	XP_001422948
	XP_003547285	-	-	XP_002307906	XP_002268970	XP_002510307	-	-	NP_001105664	-	XP_001784968	-	XP_001420946
	XP_003544007	-	-	XP_002320515	-	-	-	-	-	-	-	-	-
**ADA3**	XP_003536708	XP_002869409	ND	XP_002311946	XP_002265763	XP_002525000	XP_003572250	XP_002445585	ND	XP_002968380	XP_001782560	ND	ND
	XP_003539168	-	-	-	-	-	-	-	-	XP_002965949	-	-	-
	XP_003555871	-	-	-	-	-	-	-	-	-	-	-	-
**GCN5**	XP_003520580	XP_002876262	XP_003628592	XP_002306812	XP_002275146	XP_002520973	XP_003573924	XP_002464623	NP_001105145	XP_002960878	XP_001766378	XP_001696370	XP_001419344
	XP_003553477	-	-	-	-	-	-	-	-	XP_002967134	-	-	-
**UBP22**	XP_003550210	XP_002871441	XP_003588879	XP_002309685	XP_002283376	XP_002515408	XP_003579384	XP_002448634	NP_001132802	XP_002973842	XP_001765324	XP_001692784	XP_001420878
	XP_003544592	-	XP_003609454	XP_002324922	XP_003633155	XP_002530760	-	-	-	XP_002983550	-	XP_001702430	XP_001420231
	XP_003549730	-	-	XP_002324616	-	-	-	-	-	-	-	-	-
	XP_003542653	-	-	-	-	-	-	-	-	-	-	-	-
**SGF11**	NP_001241902	XP_002864581	XP_003594873	XP_002299995	XP_003632167	XP_002516646	XP_003568653	XP_002439607	NP_001144045	XP_002963785	XP_001779739	XP_001703794	ND
	NP_001241613	-	XP_003603196	XP_002313242	-	-	XP_003575785	-	-	XP_002974874	XP_001754483	-	-
	-	-	-	-	-	-	-	-	-	-	XP_001760795	-	-
**ENY2**	XP_003547647	XP_002877018	ND	XP_002328888	XP_002269535	XP_002509517	XP_003564943	XP_002458995	NP_001148745	XP_002960528	XP_001764723	XP_001701309	ND
	NP_001236339	-	-	XP_002298626		XP_002514922	-	-	-	XP_002967190	XP_001759104	-	-
**CHR5**	XP_003519517	XP_002885872	XP_003617298	XP_002313369	XP_002275100	XP_002531123	XP_003562521	XP_002463329	NP_001105087	XP_002969372	XP_001767461	XP_001703254	XP_001418535
	XP_003545390	-	XP_003600162	-	-	-	-	-	-	XP_002970703	XP_001782004	-	-
**TAF5**	XP_003526182	XP_002872141	XP_003603301	XP_002309672	XP_003631761	XP_002515435	XP_003563321	ND	NP_001183382	XP_002974112	XP_001769775	XP_001696990	XP_001420161
	XP_003522395	-	-	XP_002324907	XP_002285276	-	-	-	-	XP_002968859	-	-	-
	XP_003549326	-	-	-	-	-	-	-	-	-	-	-	-
**TAF6**	XP_003518649	XP_002892254	XP_003621904	XP_002320500	XP_002264290	XP_002528944	XP_003577929	XP_002459984	ACL54361	XP_002969840	XP_001762306	XP_001692591	XP_001421048
	XP_003551737	-	XP_003600186	XP_002298845	XP_002276969	XP_002531209	-	-	-	XP_002985176	-	-	-
	XP_003551827	XP_002891885	-	-	-	-	-	-	-	-	-	-	-
**TAF9**	NP_001236385	XP_002894479	XP_003598543	XP_002299587	XP_002273931	XP_002521322	XP_003557734	XP_002467704	NP_001130845	XP_002967605	XP_001785776	XP_001702038	XP_001421943
	NP_001235586	XP_002891803	XP_003635996	-	-	-	XP_003578007	-	-	-	-	-	-
**TAF10**	NP_001236890	XP_002867282	XP_003624232	XP_002324360	XP_002267115	XP_002515379	XP_003557439	XP_002462422	NP_001148356	XP_002975098	XP_001781637	XP_001697833	XP_001415768
	XP_003524747	-	-	-	XP_002266754	-	-	XP_002460155	-	XP_002963932	-	-	-
**TAF12**	XP_003542594	XP_002884775	XP_002308150	XP_002304140	XP_002277150	XP_002528715	XP_003564657	XP_002458799	NP_001169752	XP_002975297	XP_001781440	XP_001692926	XP_001415532
	XP_003528481	XP_002892951	XP_003627263	XP_002299594	-	XP_002521336	XP_003577014	XP_002458756	-	-	-	-	-
**TRA1**	XP_003517177	XP_002867036	XP_003612164	XP_002327756	XP_003631895	XP_002521662	XP_003559884	XP_002463283	NP_001105293.1	XP_002972813	XP_001764071	XP_001701957	XP_001419308
	XP_003537633	XP_002886137	-	XP_002304328	-	-	-	-	-	XP_002984389	-	-	-
**SGF29**	XP_003547706	XP_002870694	ND	XP_002298555	XP_003633806	XP_002521490	XP_003576694	XP_002443131	NP_001141068	XP_002968194	XP_001755688	ND	XP_001417743
	XP_003547704	XP_002877055	-	-	XP_003633807	-	-	-	-	-	XP_001785583	-	-
	XP_003553408	-	-	-	-	-	-	-	-	-	-	-	-
	XP_003547705	-	-	-	-	-	-	-	-	-	-	-	-

^**a**^Gm, *Glycine max*; Al, *Arabidopsis lyrta*; Mt, *Medicago truncatula*; Pt, *Populus trichocarpa*; Vv, *Vitis vinifera*; Rc, Ricinus communis; Bd, *Brachypodium distachyon*; Sb, *Sorgum bicolor*; Zm, *Zea mays*; Sm, *Selaginella moellendorffii*; Pp, *Physcomitrella patens*; Cr, *Chlamydomonas reinhardtii* and Ol, *Ostreococcus lucimarinus*.

ND: Not detected.

The phylogenetic trees were also constructed using the representative domain sequences of each protein of the SAGA complex of *Arabidopsis*, *D*. *melanogaster*, mammals (*H*. *sapiens* and *M*. *musculus*), *O*. *sativa* and *S*. *cerevisiae* (S3 Fig). The analysis of phylogenetic tree from different domain of ADA protein groups of the SAGA complex showed two clades, the first clade comprised of ADA3, GCN5, SWRIM-ADA2b and SANT-ADA2b that represents HAT modules of the SAGA complex. However, the ZZ-ADA2b domain, which is involved in interaction with GCN5, presented with ADA1 domains in the second clade (S3A Fig). The phylogenetic tree analysis of full length protein sequences indicated that ADA1 forms a different group from other ADA protein groups (Fig 2). The apparent reason behind the presence of two ZZ-ADA2b and ADA1 domains in one group might be that both protein domains are involved in protein-protein interactions. The phylogenetic tree constructed from each domain of the DUBm SAGA complex subunits suggested that SGF11 and Peptidase C19D-UBP domains were present in the same clade (S3C Fig), which is against the result obtained in the phylogenetic tree with full length protein (Fig 3A). TAFs domain grouped according to their domain features, for example, histone-fold domain containing TAF6, TAF9 and TAF12 domain were present in the same clade, in which TAF6 and TAF9 contains similar histone-fold domain (S3G Fig) [59]. TAF5 and TAF10 proteins were present in the same sub-group (S2 Fig) and their domains (NTD-TAF5 and TAF10) also showed a close relation (S3G Fig).

Notably, several paralogs were found for SAGA complex components in selected plants, mainly in *G*. *max* and *P*. *trichocarpa* (Table 3). Moreover, a variation was observed in the total number of SAGA complex components among dicots, as compared to monocots (Table 3). The variation in the number of the SAGA complex subunits suggested that these components could have been executed to accomplish the distinct and specialized roles in plants.

### Chromosomal distribution and functional annotation of plant SAGA complex

The *Arabidopsis* Genome Initiative provides the opportunity to identify the instances of chromosomal block duplication in the genome [64]. We intended to investigate, whether proteins encoding for the SAGA complex are associated with chromosomal block duplication in *Arabidopsis*. We used TAIR chromosome map viewer and Paralogons in *Arabidopsis* for the localization of the SAGA components across the five chromosomes (S4 Fig). Most of these SAGA complex proteins were in the duplicated segmental regions of *Arabidopsis* chromosome [36]. Moreover, we also identified that some of the *Arabidopsis* and *O*. *sativa* SAGA subunits were found in more than one copy such as ADA1, TAF6, TAF9, SFG29 and TRA1. Thus, it seems that some of the SAGA proteins were duplicated during evolution.

**Fig 4:**
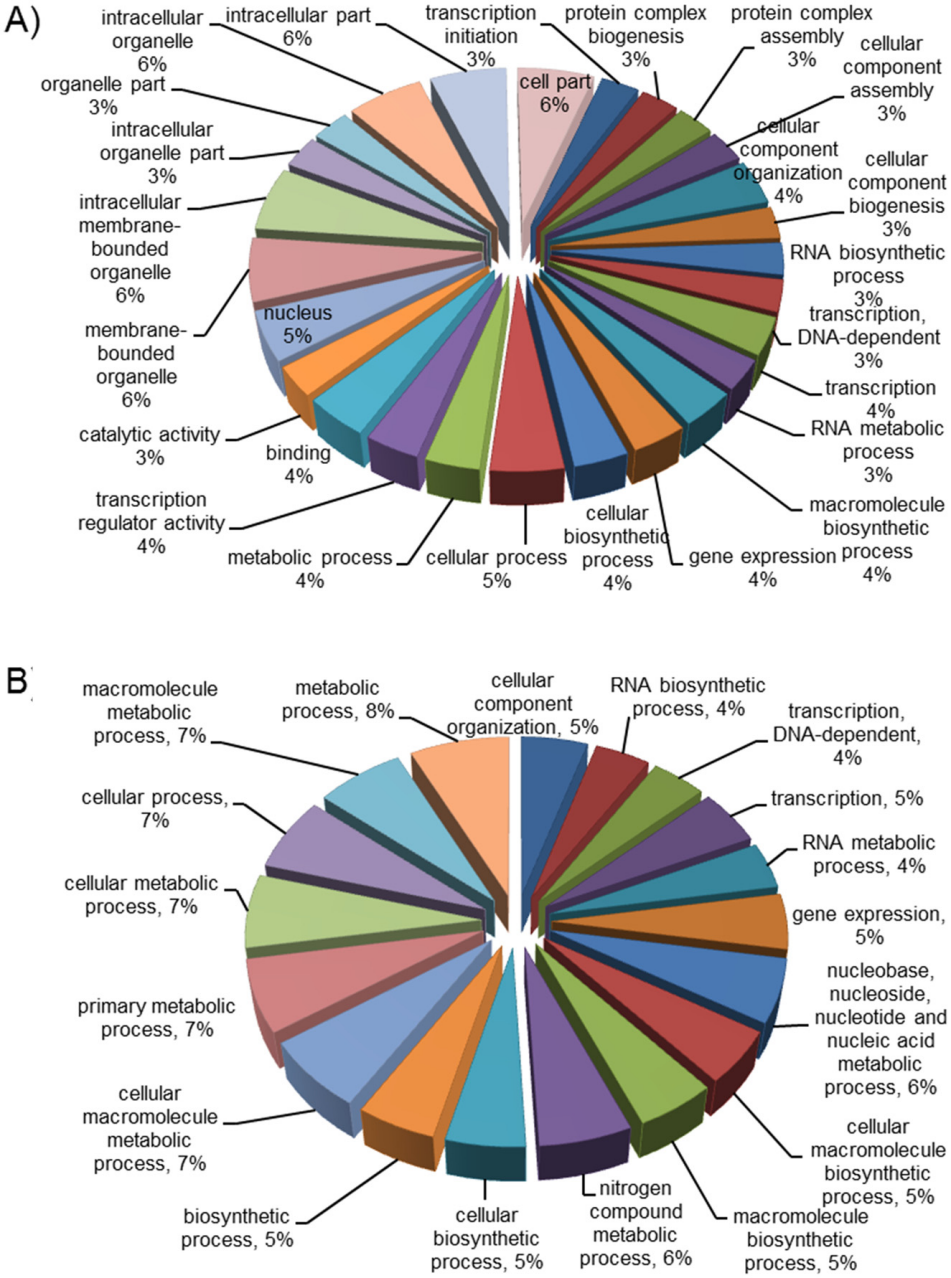
Functional annotation of plant SAGA complex components. **(A)** *Arabidopsis* **(B)** *O. sativa*. Functional annotations were performed by TAIR and agriGO databases. The percentage (%) associated with each annotation indicates the percentage of segments annotated to that category.

The functional characterization analysis showed that *Arabidopsis* and *O*. *sativa* SAGA complex components play a key role in gene expression, transcription initiation, complex assembly and several metabolic and cellular processes (Fig 4). Gene Ontology predicted that plant SAGA complex components also participate in a transcription regulator activity, binding, catalytic activity as well as in the development of cell and organelle parts (S4 Table). Recent studies suggest participation of some of the plant SAGA complex subunits, for example *Arabidopsis* ADA2B, SGF29 and GCN5, in the light- [29], cold- [28, 65] and salt-induced [61] gene expression, flower development [66], histone acetylation [30, 45]. The functional characterization analysis also indicated that their involvement in auxin, cytokinin, ethylene and jasmonic acid mediated signaling pathways (S4 Table). In sum, functional and GO analysis predicated the involvement of the plant SAGA complex not only in chromatin remodeling, but also in abiotic and biotic processes.

### Protein-protein interactome analysis of *Arabidopsis* SAGA complex

**Fig 5:**
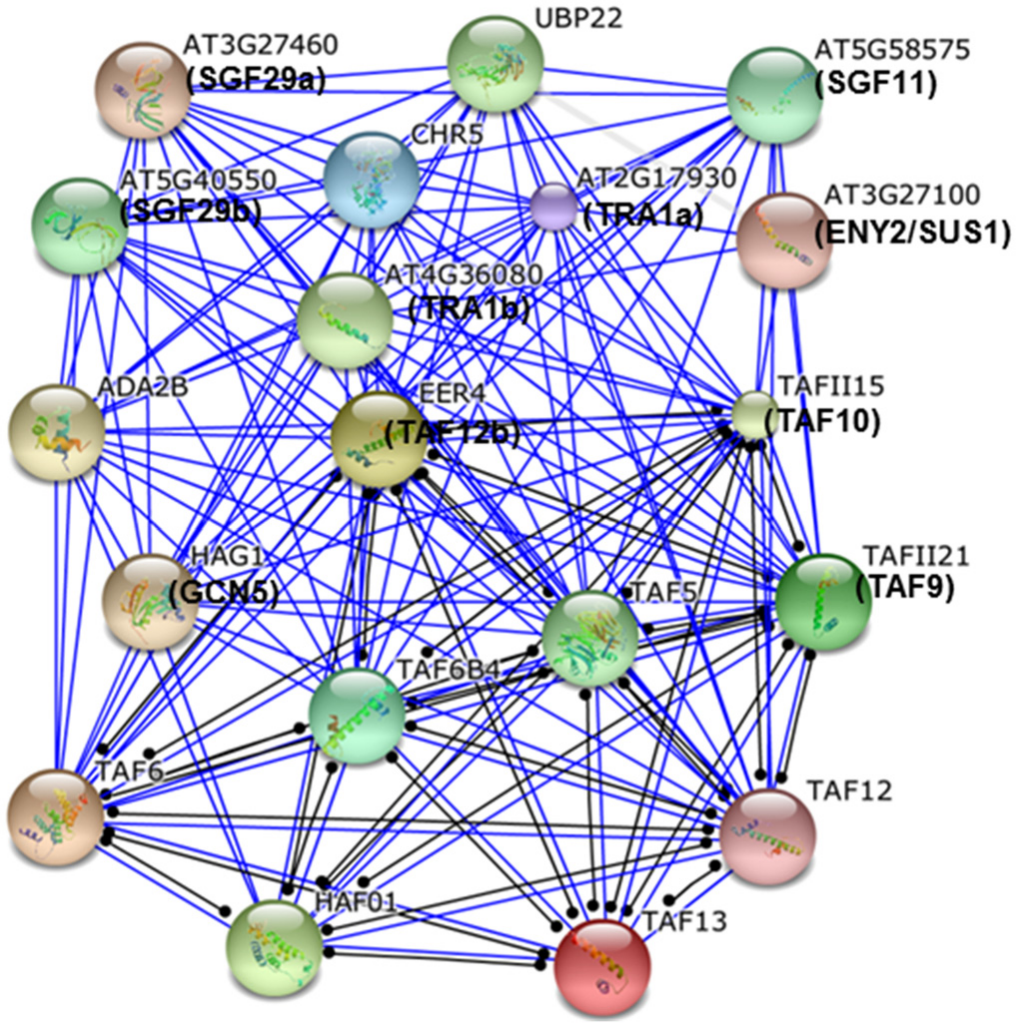
Interactome of the SAGA complex subunits. SAGA complex subunits interactomes were obtained from STRING database. Interactome between the protein pair is shown.in a confidence view where associations are represented by blue and black lines. Blue lines suggested the binding and black lines suggest a reaction between the proteins. The protein-protein network among SAGA component genes was analysed with high confidence of score 0.6.

To examine interactions among *Arabidopsis* SAGA complex components, we mapped the SAGA proteins over STRING interactome, a database of known and predicted protein interactions [41]. The analysis of *Arabidopsis* SAGA component proteins revealed an interconnected sub-network of 131-hub proteins (confidence score 0.6, Fig 5 and S5 Table). These analyses suggested that many hub proteins create a network which behaves as a functional module within the complex. Moreover, the protein-protein interaction analysis of *S*. *cerevisiae* SAGA proteins using the STRING database displayed 190-protein interactions with a confidence score of 0.6 (S6 Table). Interestingly, most of these protein-protein interactions were similar in the SAGA proteins of *Arabidopsis* and *S*. *cerevisiae* (S5 and S6 Tables). The mutation and biochemical characterization studies in *S*. *cerevisiae* and mammals established that these interactions are essential for SAGA structure and its stability. For instance, any alteration in SPT7, SPT20, TAF5, TAF10, or TAF12 affects the SAGA composition and integrity [67–69].

### *In silico* expression analysis of the SAGA complex encoding genes

**Fig 6:**
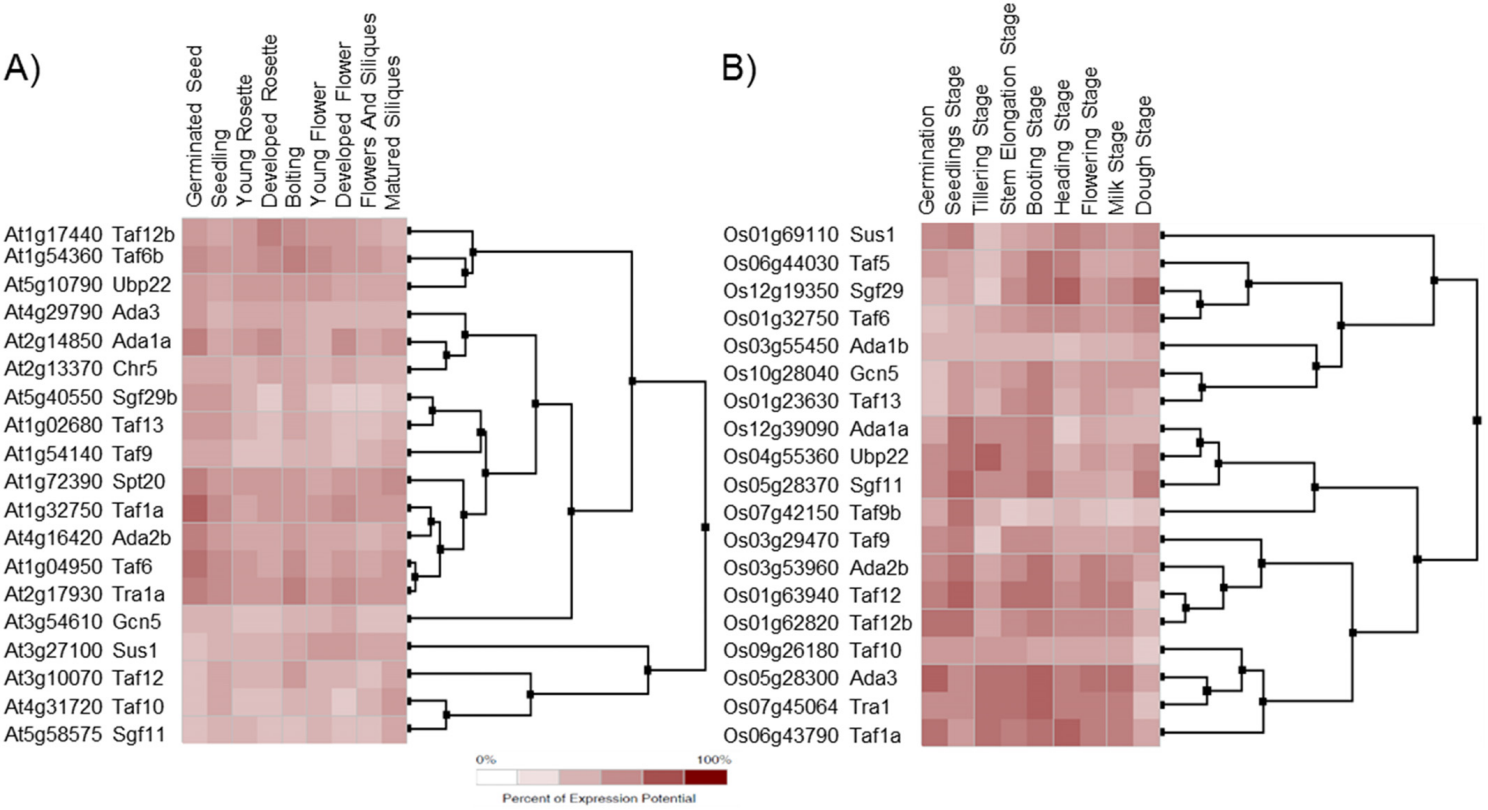
*In silico* expression patterns of the SAGA complex genes in developmental tissues. The expression patterns in different developmental tissues for SAGA genes were obtained from the Genevestigator microarray database tool. **(A)** *Arabidopsis* developmental tissues; **(B)** *O*. *sativa* developmental tissues.

**Fig 7:**
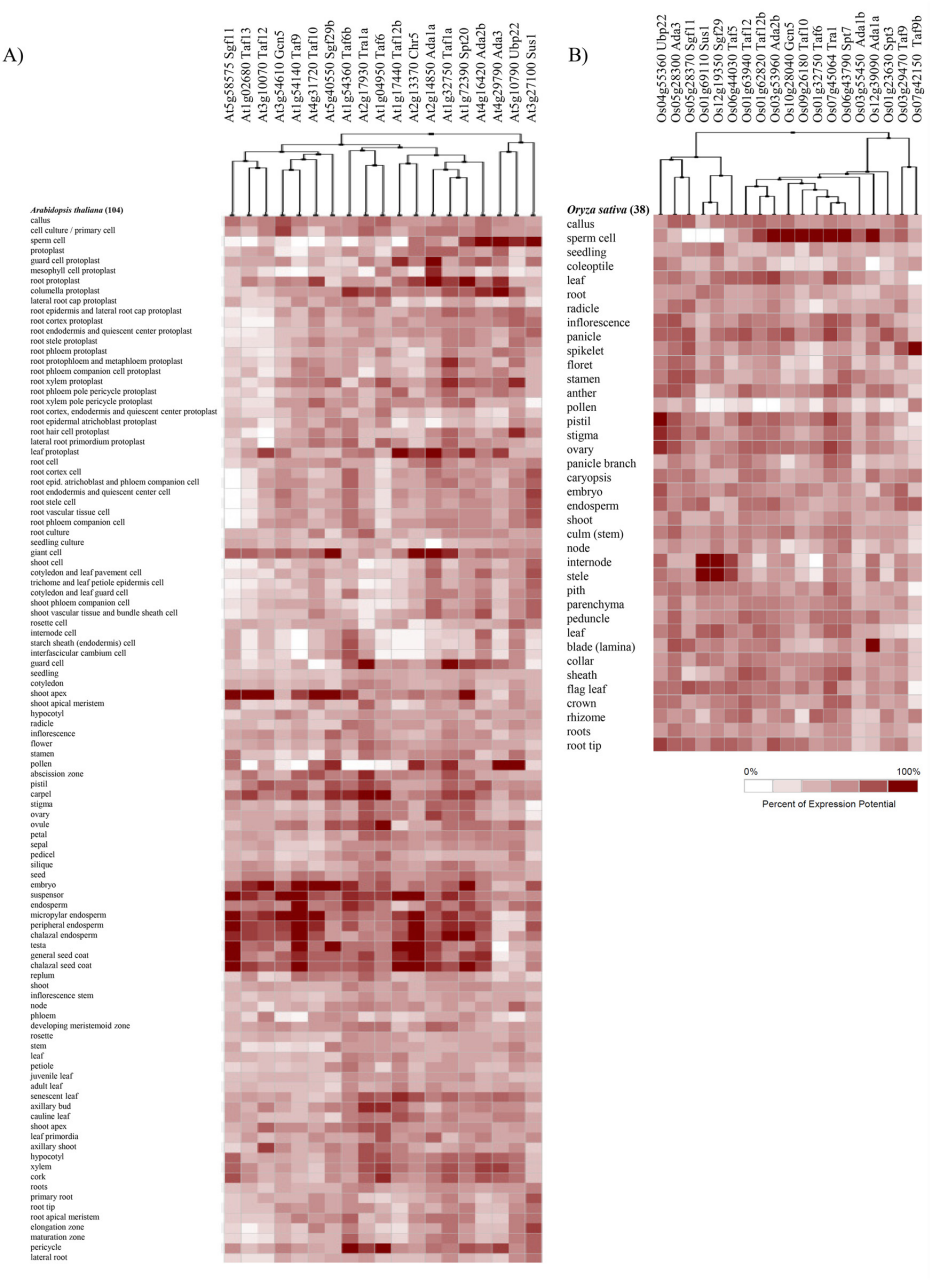
*In silico* expression patterns of the SAGA complex genes in anatomical tissues. The expression patterns in different anatomical tissues for SAGA genes were obtained from the Genevestigator microarray database tool. **(A)** *Arabidopsis* anatomical tissues; **(B)** *O*. *sativa* anatomical tissues.

Gene expression profiles of the SAGA complex components can provide significant evidences for their potential functional roles. The functional annotation of SAGA components in *Arabidopsis* and *O*. *sativa* revealed their diverse roles in plant development (Fig 4). To further validate, we examined the expression profile of the SAGA complex components in different tissues using Genevestigator microarray database and its expression meta-analysis tool [54], and MPSS database [55]. The expression profile of the SAGA complex encoding genes was examined in 9 different plant organs of *Arabidopsis and O*. *sativa* (Fig 6). *AtTaf10*, *AtGcn5* and *AtChr5* were expressed at low levels in all the examined developmental stages (Fig 6A). However, the expression of *AtAda1a*, *AtTaf12b*, *AtTaf6b* and *AtTra1a* was higher in the aforesaid developmental stages. In the case of *O*. *sativa*, SAGA subunit genes were found highly expressed in booting, seedling, milk, flower and stem elongation stages (Fig 6B). During germination, transcript accumulation was observed at higher levels for *AtTaf6/6b*, *AtTra1a*, *AtAda2b*, *AtTaf1a*, *AtTaf12b* and *AtUbp22* genes in *Arabidopsis*, whereas, for *OsAda2b*, *OsAda3*, *OsTaf5*, *OsTaf12/12b*, *OsTaf1a*, *OsUbp22*, *OsSus1*, *OsSgf11* and *OsTaf9* genes in *O*. *sativa*. In the booting stage of *O*. *sativa*, *OsTaf5*, *OsTaf13*, *OsTaf12/12b*, *OsGcn5*, *OsAda2b*, *OsAda1a*, *OsSgf29*, *OsAda3*, and *OsSgf11* were among the highly expressed genes, whereas, *OsSus1*, *OsSgf29*, *OsUbp22*, *OsTaf6* and *OsSgf11* were the genes that highly expressed during dough developmental stages. The meta-analysis displayed an enhanced expression of SAGA component genes in the endosperm (micropylar, peripheral and chalazal), seed coat, suspensor callus and primary cells of *Arabidopsis* (Fig 7A), whereas in callus, sperm cells, panicle, leaf, pistil, stigma, ovary and root tip of *O*. *sativa* (Fig 7B). The results suggest a diverse role of SAGA component genes being expressed throughout different developmental phases in distinct plant organs and tissues.

Data was extracted from the MPSS database library (17 and 20 bases), representing 12 and 13 different anatomical parts of *Arabidopsis* and *O*. *sativa*, respectively. These signatures uniquely recognize specific gene, which show a perfect match (100% identity over the tag length), and signify a quantitative estimation of expression of that gene. These MPSS tags further confirmed transcript abundance of SAGA protein encoding genes in different plant parts (S7 and S8 Tables). Transcript differences are generally presented by the total number of tags (TPM, transcripts per million), low expression if smaller than 25 TPM, moderate expression if 26 to 250 TPM, while highly expressed in case of >250 TPM. Based on these signatures/tags, five *Arabidopsis* genes *viz*., *AtAda3*, *AtAda1b*, *AtTaf12*, *AtTra1a* and *AtSgf29* were expressed at low levels, whereas *AtTaf10* expressed at a higher level in leaf, root, siliques and callus (S7 Table). Other *Arabidopsis* SAGA genes exhibited a moderate level of transcript accumulation. MPSS analysis in *O*. *sativa* showed that *OsAda2b*, *OsTaf10* and *OsTra1* expressed at higher levels (>250 TPM). The maximum transcript abundance was observed for *OsAda2b* in mature leaves and for *OsTaf10* in young leaves, ovary and mature stigma and callus, whereas *OsTra1* was significantly expressed in most of the plant parts, except germinating seed and stem. The SAGA genes, *OsGcn5*, *OsAda1a*, *OsSpt20*, *OsSgf11*, *OsTaf9b* and *OsTaf12* expressed at low levels, whereas others at moderate levels (S8 Table).

### Co-expression analysis for gene pairs and gene network analysis of the SAGA complex

The expression profiles of SAGA components in *Arabidopsis* and *O*. *sativa* using Genevestigator and MPSS revealed that many of the components have distinct tissue-specific expressions. We further examined whether these genes co-express during plant development or in any other physiological condition. The co-expression data for each SAGA component gene pairs were generated from ATTED-II database, which includes 1388 microarray experimental data [43]. The strength of co-expression for the interconnecting gene pairs was determined by Mutual Rank (MR) process using these microarray data. Forty-two significant co-expression patterns (Table 4) were obtained between SAGA components from 171 co-expressing gene pairs (S9 Table). These co-expression patterns were identified under different biotic, abiotic, hormonal and tissue conditions, for example, co-expression analysis of gene pairs data showed that *Ada2b* was strongly co-expressed with *Taf6*, *Taf13* and *Spt20* genes in all the developmental and environmental stress conditions. Likewise, *Ubp22* co-expressed with *Sgf29b* and *Taf1b* with *Tra6* and *Taf13*, at high MR values. The significant MR values for *Taf1b*, *Spt20*, *Tra1*, *Taf9*, *Gcn5*, *Ada2b* and *Ada3* suggest their co-expression at the tissue level. The genes, *Spt20*, *Ada2b*, *Taf12b*, *Chr5*, *Taf9* and *Taf10* showed co-expression in abiotic stress conditions (Table 4). Under hormonal condition, *Ada2b*, *Ada3*, *Chr5*, *Taf6*, *Taf10*, *Taf12b*, *Taf13* and *Tra1a* exhibited a substantial level of co-expression strength, whereas *Spt20* was found to be co-expressed with *Chr5* in biotic stress condition (Table 4).

**Table 4:** Co-expression analysis of SAGA component genes in *Arabidopsis*.

S.N.	LOCUS1		LOCUS2		Mutual Rank (MR)^a^				
					All	Tissue	Abiotic	Biotic	Hormone
1	At1g04950	TAF6	At4g16420	ADA2B	**42.5**	**706.1**	**469.5**	2967.2	**59.4**
2	At1g02680	TAF13	At4g16420	ADA2B	**47**	**143.4**	**451.7**	**706.6**	**10.2**
3	At1g72390	SPT20	At4g16420	ADA2B	**59**	**625.7**	**59.7**	**532.6**	**555.5**
4	At5g10790	UBP22	At5g40550	SGF29B	**100**	4698.1	**223.4**	3121.7	8550.2
5	At1g04950	TAF6	At2g17930	TRA1A	**137.2**	**150.1**	1762	**849.3**	3776.9
6	At1g02680	TAF13	At2g17930	TRA1A	**158.3**	**24.1**	6755.4	1645.4	6751.4
7	At1g72390	SPT20	At4g29790	ADA3	**164.7**	**121.5**	**720.7**	2500.2	1786.6
8	At1g32750	TAF1B	At4g16420	ADA2B	**187**	**554.3**	**613.6**	2615.8	10053.6
9	At2g13370	CHR5	At4g16420	ADA2B	**187.3**	1798.5	**270.9**	**358.2**	2263.7
10	At1g54140	TAF9	At3g54610	GCN5	**256.6**	**92.3**	5502.3	3446.5	2020.6
11	At1g32750	TAF1B	At1g72390	SPT20	**258.8**	**5.5**	1702.5	1398.6	17199.5
12	At1g17440	TAF12B	At2g13370	CHR5	**261.8**	3768.4	**129**	1889.8	**19.6**
13	At1g02680	TAF13	At1g32750	TAF1B	**285.5**	**744.5**	2116	2013.9	1885.3
14	At1g72390	SPT20	At2g13370	CHR5	**296.7**	**959.4**	**206.5**	**140.8**	3661.9
15	At4g16420	ADA2B	At4g29790	ADA3	**315.6**	2734.4	**966.2**	**976.3**	**84.4**
16	At1g02680	TAF13	At1g04950	TAF6	**330.5**	**346.1**	1827.7	1061.4	**523.2**
17	At1g02680	TAF13	At3g27100	SUS1	**334.8**	2570.5	**345.5**	**508.5**	6808.6
18	At1g32750	TAF1B	At2g17930	TRA1A	**339.7**	**912.1**	**889.7**	2158.4	5755.3
19	At2g17930	TRA1A	At4g16420	ADA2B	**358.8**	**124.2**	4677.5	2905.3	1184.4
20	At1g04950	TAF6	At4g31720	TAF10	**377.1**	**864.8**	2178.7	**634.8**	**32.6**
21	At1g02680	TAF13	At1g54140	TAF9	**430.9**	**142.8**	1530.5	15622.6	3374
22	At1g17440	TAF12B	At1g72390	SPT20	**435.2**	1511	1180.6	1791.3	1163.2
23	At2g13370	CHR5	At4g29790	ADA3	**469.1**	**373.4**	1177.1	2125	**843.3**
24	At1g02680	TAF13	At4g31720	TAF10	**482**	2554	**228.8**	**427.2**	1064.2
25	At1g32750	TAF1B	At2g13370	CHR5	**489.7**	1185	3523.9	1579	2548.7
26	At1g72390	SPT20	At2g17930	TRA1A	**491.9**	**929.8**	7664.1	2540.6	**382.9**
27	At1g17440	TAF12B	At2g14850	ADA1A	**509.9**	**563.8**	2349.7	**328**	12012.7
28	At1g54140	TAF9	At4g31720	TAF10	**543.1**	**547.6**	**137.8**	18320.2	**280.8**
29	At1g32750	TAF1B	At5g58575	SGF11	**573.2**	1972.6	1479.7	1786.7	12185
30	At3g10070	TAF12	At5g40550	SGF29B	**576.3**	1814.2	**466.1**	1005.4	19138.8
31	At1g17440	TAF12B	At4g29790	ADA3	**601.2**	1368.2	1636.9	2773	**572.6**
32	At1g54360	TAF6B4	At2g17930	TRA1A	**613.1**	2589.2	1343.7	1096.5	1074.8
33	At1g72390	SPT20	At2g14850	ADA1A	**659.8**	**224.1**	2409.6	2249.2	4698.8
34	At1g17440	TAF12B	At2g17930	TRA1A	**718.4**	1106.6	5512.9	1279.2	**117.6**
35	At1g02680	TAF13	At2g14850	ADA1A	**777.9**	**446.8**	5712.8	6936.2	11359.6
36	At1g32750	TAF1B	At2g14850	ADA1A	**780.8**	**129.4**	7427.5	1777.1	1345.7
37	At1g17440	TAF12B	At4g16420	ADA2B	**785.6**	1057.4	3349.8	2005.7	1624.9
38	At3g27100	SUS1	At4g31720	TAF10	**793.1**	3246.7	**821**	**350.5**	5879.1
39	At2g14850	ADA1A	At4g16420	ADA2B	**872.4**	**652.8**	**965.4**	**326.1**	12755.7
40	At1g54360	TAF6B4	At3g54610	GCN5	**897**	1218.2	**422.4**	1624.3	8621.1
41	At1g32750	TAF1B	At4g29790	ADA3	**897.7**	**186.4**	9527.1	1397.2	12528
42	At1g02680	TAF13	At1g72390	SPT20	**923.8**	1201.9	4235.8	**744.2**	7934.4

^a^ MR values represent here <1000 shows significant co-expression data (in bold) [43]

The co-expressed gene network analysis was done to identify the genes, which co-regulate with the SAGA complex (S5 Fig). Co-expressed gene network provides the evidence of highly interconnected expression modules of a subset of genes, which additionally show another layer of regulation, and consequently the complementary evidences to understand gene function network. A total of 181 proteins was found to be co-regulated with SAGA complex components (S10 Table). Approximately 36% of 181 proteins are recognized to be involved with regards to abiotic or biotic stimulus or stress, developmental processes, transcription regulation, signal transduction and other biological processes (S6 Fig). This analysis further indicates a potential role of the SAGA complex in regulating plant development and responses to various physiological stresses.

### Expression analysis of the SAGA complex subunit during developmental stages and stress conditions

**Fig 8:**
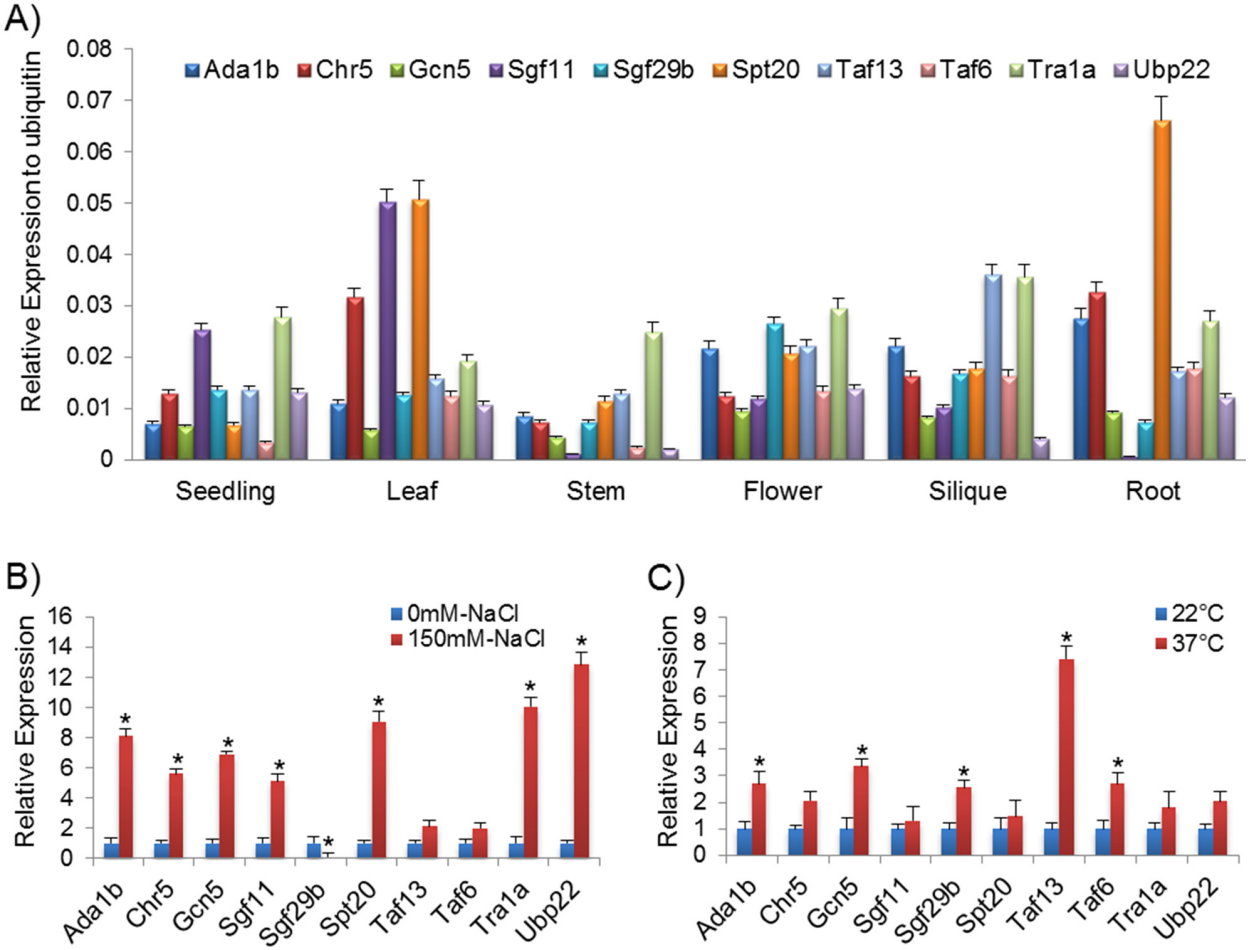
Real time PCR for *Arabidopsis* SAGA complex subunits in different development tissues and stress conditions. **(A)** The expression pattern of the SAGA complex subunits in different tissues. Relative expression analysis of each was plotted with reference to the expression of ubiquitin (either 6-day-old light-grown seedlings 16 / 8hr, mature leaves, stems, flowers, siliques and roots). **(B)** The expression pattern of *Arabidopsis* SAGA complex subunits in response to abiotic salt stress conditions. *Arabidopsis* Col-0 leaves treated with MS liquid medium (as a control) and NaCl (150 mM in MS media). **(C)** The expression pattern of *Arabidopsis* SAGA complex subunits in response to high temperature conditions. *Arabidopsis* Col-0 leaves kept in MS liquid medium at 22°C (as a control) and 37°C (2 hr in MS media). The asterisks (*) denote *P ≤* 0.01.

We performed a quantitative gene expression analysis of ten representative SAGA components by QRT-PCR in different *Arabidopsis* tissues: flowers, mature leaves, siliques, six-day-old seedlings, stems and roots (Fig 8A). The gene expression profile of the SAGA complex components was plotted with reference to the expression of ubiquitin. The genes of the SAGA complex, although expressing at a lower level compared with Ubiquitin, showed consistent expression in almost all the examined plant parts (Fig 8A). These indicate the involvement of the SAGA complex in gene regulation throughout the plant body. However, there were certain components that showed spatial preference, for example, the expression of *Spt20* was relatively higher in root and leaf, whereas, *Sgf11* in leaves and seedlings than other examined tissues.

The effect of high temperature and salt concentrations was also examined on the expression pattern of the SAGA components in *Arabidopsis*. The excised leaves of *Arabidopsis* were either exposed to a high temperature at 37°C for 2 hr or 150 mM NaCl for a period of 24 hr for high salt stress condition, and the gene expression of the SAGA components was compared with their respective controls. The gene expression of the most of selected components of the SAGA complex was induced under elevated salt concentration (Fig 8B) and high temperature (Fig 8C); however, the fold of induction varies for different components. Interestingly, *Sgf29b* expression was suppressed in salt treatment condition (Fig 8B). Thus, the qRT-PCR results suggested the significance of SAGA components gene expression in plants during abiotic stresses.

As discussed above, *in silico* co-expression analysis of the SAGA complex subunits suggested that these subunits were co-expressed in the tissues, hormones and stress conditions. Notably, the quantitative gene expression analysis of selected SAGA components further supported the co-expression analysis, such as *Spt20* and *Chr5*, *Taf13* and *Tra1*, *Spt20* and *Chr5* showed high co-expression with significant MR value in tissue; while *Spt20* and *Ubp22*, *Sgf11* and *Tra1*, *Spt20* and *Chr5*, *Gcn5* and *Taf6* were co-expressed and considerable MR value in abiotic stress. The qRT-PCR analysis is in agreement with the *in silico* co-expression profile (Table 4, Fig 8 and S9 Table).

### SAGA complex regulates expression of heat, salt and light-induced genes

**Fig 9:**
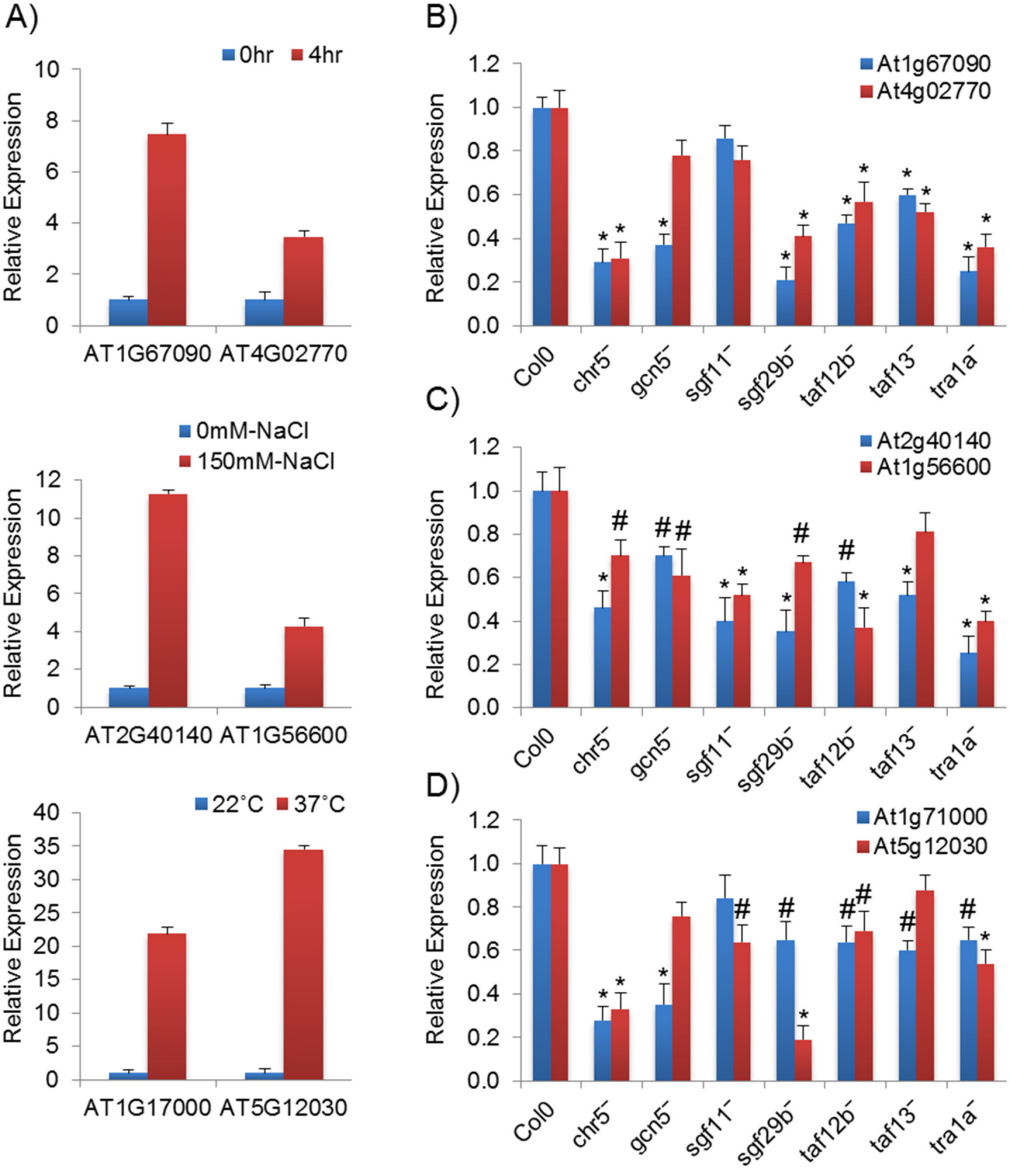
The effect of the SAGA complex on light and stress induced gene expression. For light condition, RNA was isolated from seedling grown for 5 days and then transfer into light for 4 hr. For salt condition, RNA was isolated from leaves kept in either in 0.5 x MS media (as a control) or in 0.5 x MS media with 150mM NaCl solution for 24 hr. For heat condition, RNA was isolated from *Arabidopsis* Col-0 leaves kept in MS liquid medium at 22°C (as a control) and 37°C (2 hr in MS medium). Ubiquitin used as internal control. The mark symbols denote (*)—*P ≤* 0.001; (#)—*P ≤* 0.01. **(A)** Real time PCR for different selected induced responsive genes in light, salt and heat conditions. **(B)** The effect of *Arabidopsis* SAGA complex subunit mutants on light activated gene expression. **(C)** The effect of *Arabidopsis* SAGA complex mutant subunits on salt-induced gene expression. **(D)** The effect of *Arabidopsis* SAGA complex subunits on heat-induced gene expression.

SAGA complex facilitates the PIC assembly in the core promoter region of yeast and human genes [70–74]. Little is known about how SAGA complex facilitates gene regulation in plants. To address this, RNA was isolated from seven homozygous T-DNA SAGA subunit *Arabidopsis* mutants and wild type plants, grown under different conditions such as light/dark, high salt or heat stress (Fig 9 and S7 Fig). The gene expression of light induced (At1g67090 and At4g02770) [2, 75], salt induced (At2g40140 and At1g56600) [76, 77] and heat induced (At1g71000 and At5g12030) [78] genes was examined in these mutant in comparison to the wild type plants by qRT-PCR (Fig 9A). The expression of both the light activated genes was considerably reduced in all the mutants, except in *sgf11⁻* for both the genes and in *gcn5⁻* for At4g02770, in which the relative expression values were not statistically significant (Fig 9B). In the case of salt stress, the expression level of both the salt induced genes declined in mutants as compared to the wild type, except At1g56600 in *taf13⁻*, which was statistically not significant (Fig 9C). Under heat stress, expression of the heat activated genes was decreased in mutants, except At5g12030 in *gcn5⁻* and *taf13⁻* and At1g71000 in *sgf11⁻* mutant which were not statistically significant (Fig 9D). These results anticipated that SAGA complex plays significant roles in the transcription regulation of stress inducible genes.

## Discussion

The SAGA complex has been previously shown to be associated with transcriptional regulation of ~10% RNA polymerase II-dependent *S*. *cerevisiae* genes, which contribute in response to DNA damage and other stress conditions such as heat, oxidation, and metabolic starvation [71, 72, 79]. A recent report indicates that SAGA complex regulates all active genes and present at their promoters and transcribed regions [80]. With the computational approach, we identified 18 putative SAGA complex subunits in *Arabidopsis* and *O*. *sativa*. The protein similarities among *Arabidopsis* and *S*. *cerevisiae* SAGA complex subunits are low (17%) to medium (51%), as observed between *S*. *cerevisiae* and human SAGA complex (15% to 56%; S2 Table). Since the SAGA complex is involved in the fine-tuning of gene expression, this could be one of the reasons for the poor protein similarities. Our results on *in silico* expression, GO analysis and qRT-PCR of plant SAGA complex representative genes suggested their role in various cellular, physiological and molecular processes. The previous reports on the functions of ADA2b, GCN5, TAF10, TAF6 and SGF29 in plants are in accordance with our study, suggesting conservation of the SAGA complex throughout evolution [28, 46, 61, 81–83]. Thus, the presence of conserved domain is helpful in identifying most of the putative members of plant SAGA complexes in different plant organism databases. Beside the low level similarity in full protein sequence (S2 Table), most of the domains present in plant SAGA complex encoding genes were found conserved among different organisms (Figs 2 and 3; Table 2). The similarity between conserved domain’s amino acid sequences of *Arabidopsis* SAGA was observed higher, i.e. from 30% to 97% (Table 2 and S1 Fig). Notably, similar range of similarities was found between the key domains of the SAGA complex in *S*. *cerevisiae* and human (Table 2 and S1 Fig). On the basis of protein or conserved domain similarity and phylogenetic analysis, our results altogether suggested that plant SAGA complex was observed to be closer to the human than that to the yeast SAGA complex (Figs 2 and 3; Table 2 and S2 Table).

Our analysis of protein alignment, phylogenetic tree and chromosomal distribution suggested that many plant SAGA complex representative genes might have duplicated during evolution (Figs 2 and 3; S2 Fig). For example, *Taf6*, *Taf9*, *Taf12*, *Ada1*, *Tra1* and *Sgf29* have been found duplicated in either *O*. *sativa* or *Arabidopsis*. Besides these genes, other SAGA subunit genes are also found duplicated in other lower and higher plant groups (Table 3). This duplication event may also lead to variability in the SAGA complex components in plants like Ada2a-containing (ATAC), SLIK/SALSA or STAGA [74], or sometimes shares subunits with other complexes like TFIID [68]. The protein interactome analysis suggested that *Arabidopsis* SAGA complex proteins interact with each other and thus further suggested their conservation in plants (Fig 5). The structural integrity of the SAGA complex is dependent on the protein-protein interactions as evident in our study, and also discussed in previous reports; such as TAF10 and TAF12 associate directly via their histone fold domains with SPT7 and ADA1, establishing SPT7-TAF10 and ADA1-TAF12 heterodimer, respectively [84, 85], whereas TAF5 interact with ADA1, ADA3 and SPT7 [69].

Our results suggested that the SAGA complex encoding genes expressed in most of the plant parts and playing an essential role in plant development. Previous reports in *Arabidopsis*, *gcn5⁻* exhibit pleiotropic developmental abnormalities, such as abnormal meristem role, dwarfism, loss of apical dominance, defects in floral organ identity [28, 29, 31, 86–88]. An insertion of T-DNA elements in the *Arabidopsis Ada2b* produces a dwarf phenotype with defects in root and shoot development [28, 87, 89]. *Arabidopsis sgf29a⁻* shows a little delay in leaf and flower development [61]. Importantly, some reports on plant TAFs (TAF5, 6 and 10) indicated their indispensable role in plant development [81, 83, 90]. Notably, SAGA complex is also critically involved in developmental aspects and is indispensable for viability in metazoan [11]. Recently, ubiquitin protease activity of the SAGA complex showed significant regulation of the expression of the tissues specific genes and developmental processes in *Drosophila* [91]. In *Drosophila*, loss of SAGA subunit functions, such as ADA2b, SGF11 and Nonstop protein (homolog of ENY2), display photoreceptor axon targeting defects, whereas, GCN5 has an essential role in the development of eye and wing disc [92, 93]. While, mice TAF9b and GCN5 are required for the regulation of genes during neuronal and mesoderm development [94, 95]. These accumulating evidences indicate that the functions of the SAGA complex in higher organisms involve more sophisticated mechanisms in regulation of gene expression than unicellular counterpart like *S*. *cerevisiae* during development processes.

SAGA complex expedites the gene expression that anticipates to various environmental cues such as DNA damage and abiotic stress conditions [12]. Many reports, as discussed above, reveal that the SAGA complex is directly or indirectly contributing in various developmental and stress regulated processes, for example, arsenite stress conditions [52] osmotic stress [96] and ultraviolet induced [97]. The yeast SAGA complex also takes part in the up-regulation of several genes during environmental stress, including carbon starved condition [71]. Our results support the stress inducible expression of several SAGA components in *Arabidopsis*. Interestingly, the promoter sequence analysis of the SAGA components revealed several stress responsive *cis*-motifs (S11 Table), indicating their involvement in transcription regulation activities in response to stress. Nevertheless, further experimentation is needed to validate the involvement of these motifs in the regulation of the SAGA component genes. The expression analysis of the SAGA subunits supports its potential roles in response to environmental cues (Figs 6 and 7). These results are in accordance with the earlier published reports on plant ADA2b, GCN5 and SGF29a [26, 28, 46, 61]. *Arabidopsis ada2b-1⁻* mutant displays enhanced hypersensitivity to salt and abscisic acid stress than wild-type plants [26, 61]. Although, loss of SGF29a function displays salt stress tolerance, the gene expression level of stress-related genes markers such as COR78 (cold regulated 78), RAB18 (responsive to aba 18), and RD29b (responsive to desiccation 29b) are lower in *sgf29a⁻* mutant after 3 hr of NaCl treatment [61]. *Arabidopsis* HAT protein GCN5 and co-activator ADA2b proteins play significant roles in cold responses and loss of functions of these proteins showed a decline in the expression of several cold-regulated genes [27, 28, 98]. Altogether, the property of the SAGA complex in the regulation of stress genes is not only well maintained within plants, but also comparable to *S*. *cerevisiae* or human [71, 74].

In conclusion, we identified 18 subunits of the SAGA complex in *Arabidopsis* and *O*. *sativa*. The protein similarities at the level of conserved domain indicate that the SAGA complex is conserved in eukaryotes such as *S*. *cerevisiae*, plants and mammals. The expression analysis of the SAGA components indicates that the networks of SAGA complex are involved in various biological processes in plants, including development, physiology and response to environmental stresses via gene regulation. This study advances our understanding about SAGA components and their different functions in plants.

## Supporting Information

S1 Fig Domain similarity between human, *S*. *cerevisiae*, *Arabidopsis* and *O*. *sativa* SAGA complex encoding genes. Sequence alignments of the representative domains of each protein of the SAGA complex were done by using Clustal X.
(PDF)

S2 Fig The phylogenetic relationship of the TAFs group of the SAGA complex. SAGA complex subunit protein sequences were used from At, *A*. *thaliana* (red circle); Dm, *D*. *melongaster*; Hs, *H*. *sapiens* (red square); Os, *O*. *sativa* (red diamond shape); Mm, *M*. *musculus*; Rn, *R*. *norvegicus;* Sc, *S*. *cerevisiae* (red triangle); Sp, *S*. *pombe;* Zm, *Z*. *mays;* Rc, *R*. *communis;* Pt, *P*. *trichocarpa;* Vv, *V*. *vinifera;* Al, *A*. *lyrta;* Mt, *M*. *truncatula;* Bd, *B*. *distachyon;* Sb, *S*. *bicolor;* Sm, *S*. *moellendorffii;* Pp, *P*. *patens;* Cr, *C*. *reinhardtii* and Ol, *O*. *lucimarinus*. Phylogeny reconstruction was analyzed by neighbour-joining statistical method based on the JTT matrix-based model. Test of phylogeny was analyzed by the bootstrap method (1,000 replicates). Evolutionary analyses were conducted in MEGA 6.06.
(PDF)

S3 Fig Molecular phylogenetic analysis of domains of the SAGA complex components. Amino acid sequences of domains of the SAGA complex subunits were used from At, *A*. *thaliana*; Dm, *D*. *melongaster*; Hs, *H*. *sapiens*; Os, *O*. *sativa*; Mm, *M*. *musculus*; Sc, *S*. *cerevisiae*. **(A)** Protein domain of ADAs group; **(B)** Protein domain of SPTs group; **(C)** Protein domain of DUBm group; **(D)** Protein domain of CHD subunit; **(E)** Protein domain of SGF29 subunit; **(F)** Protein domain of TRA1 subunit; **(G)** Protein domain of TAFs group.
(PDF)

S4 Fig Chromosomal distribution of the SAGA subunit genes in the *Arabidopsis* genome. SAGA encoding genes are plotted on the five *Arabidopsis* chromosomes according to their sequence spots. The chromosome number is shown at the top of each chromosome and the centromeric regions by constriction on chromosome line bar. Each identical duplicated chromosomal segment is marked by same line colour. The scale is in mega bases (Mb).
(PDF)

S5 Fig Co-expressed gene network analysis for *Arabidopsis* SAGA complex. The co-expressed gene networks are drawn based on their rank of correlation from ATTED-II database. Orange line displays conserved co-expressed which is inferred from the comparison with mammalian co-expression data provided from COXPRESdb; Red dotted line display protein-protein interaction information that is provided from TAIR and IntAct. The octagon shape indicates transcription factor genes. White circles shape indicates SAGA complex genes which were used to give input for generating gene network. Gray circle shape indicates other genes in co-expressed gene networks.
(PDF)

S6 Fig Analysis of the biological process of co-expressed gene network. A biological process is analyzed by TAIR database using 181 co-expressed genes obtained from ATTED-II database.
(PDF)

S7 Fig Characterization of *Arabidopsis* mutant lines. Characterization of *Arabidopsis chr5‾*, *gcn5 ‾*, *sgf11‾*, *sgf29b‾*, *taf12b‾*, *taf13‾* and *tra1a‾* T-DNA insertion homozygous mutants were done by qRT-PCR. RNA was isolated from homozygous T-DNA insertion mutants and Col-0 leaves or seedlings.
(PDF)

S1 Table Query sequences from *S*. *cerevisiae* and human used to search *Arabidopsis* and *O*. *sativa* genome for SAGA gene families. (PDF)

S2 Table Protein similarities of SAGA encoding gene in *Arabidopsis*, *O*. *sativa*, human and *S*. *cerevisiae*. (PDF)

S3 Table List of primer used in qRT-PCR. (PDF)

S4 Table Gene Ontology list of *Arabidopsis* and *O*. *sativa* for SAGA complex gene. (PDF)

S5 Table List of *Arabidopsis* SAGA complex subunits known and predicted protein interactions from STRING database. (PDF)

S6 Table List of *S*. *cerevisiae* SAGA complex subunits known and predicted protein interactions from STRING database. (PDF)

S7 Table MPSS data for *Arabidopsis* SAGA complex encoding genes showing different tissue-specific abundance. (PDF)

S8 Table MPSS data for *O*. *sativa* SAGA complex encoding genes showing different tissue-specific abundance. (PDF)

S9 Table List of co-expression genes in *Arabidopsis*. (PDF)

S10 Table List of genes obtained from ATTED-II for *Arabidopsis* SAGA complex co-expressed gene network analysis. (PDF)

S11 Table Analysis of cis-regulatory element in 1000bp upstream promoter sequences from TSS in SAGA complex subunit genes using PlantCARE and PLACE database. (PDF)
