# Supplementary material for: Spt-Ada-Gcn5-Acetyltransferase (SAGA) Complex in Plants: Genome Wide Identification, Evolutionary Conservation and Functional Determination
Source: PLoS One. 2015 Aug 11;10(8):e0134709. doi: 10.1371/journal.pone.0134709 (PMC4532415; doi:10.1371/journal.pone.0134709)
Supplement: S1 Fig — Sequence alignments of the representative domains of each protein of the SAGA complex were done by using Clustal X. (PDF) [file pone.0134709.s001.pdf]

# S1 Fig.

## >GCN5_BR

```
AT_br_459-559      ----------QLNALMRALLKTMQDHADAWPFKEPVDSRDVPDYYDIIKDPIDLKVIAKRVESEQYYVTLDMFVADARRMFNNCRTYNSPDTIYYKCATRLETHFHSKVQA--------
Os_br_402-500      ----------QLTNLMRSLLKNMNEHPDAWPFKEPVDSRDVPDYYDIIKDPIDLKTMSKRVESEQYYVTLEMFVADMKRMFSNAKTYNSPDTIYYKCASRLESFFSNKVASQLAQASTK
HS_br_727-826      ----------QLYSTLKSILQQVKSHQSAWPFMEPVKRTEAPGYYEVIRFPMDLKTMSERLKN-RYYVSKKLFMADLQRVFTNCKEYNPPESEYYKCANILEKFFFSKIKE--------
Sc_br_331-431      DALAQRPKRGPHDAAIQNILTELQNHAAAWPFLQPVNKEEVPDYYDFIKEPMDLSTMEIKLES-NKYQKMEDFIYDARLVFNNCRMYNGENTSYYKYANRLEKFFNNKVKEI-------
Clustal Consensus                    :: :*  ::.*  **** :**.  :.*.**:.*: *:**..:  :::. . * . . *: * : :*.*.: **  :: *** *. **..* .*:
```

## >Gcn5_NAT_SF

```
At-Nat-sf_264-318  SVMVLRGNL-VVGGITYRPYHSQKFGEIAFCAITADEQVKGYGTRLMNHLKQHARD---
Os-Nat-sf_211-263  ---VIRNNI-VVGGITYRPYTSQKFGEIAFCAITADEQVKGYGTRLMNHLKQHARDA--
Sc-Nat-sf_148-202  -MAVIRKPLTVVGGITYRPFDKREFAEIVFCAISSTEQVRGYGAHLMNHLKDYVRN---
Hs-Nat-sf_546-602  -LALIKDGR-VIGGICFRMFPSQGFTEIVFCAVTSNEQVKGYGTHLMNHLKEYHIKHDI
Clustal Consensus      :::    *:*** :* : .: * **.***::: ***:***::******::  .
```

## >Taf5_NTD2

```
At_Taf5_NTD2-64-195   EDDPTRYREGYSKLRSWAYNSLDLYKHELLRVMYPVFIHCYMDLVGKGHTQEARAFFNSFRKDHEMVHLRDLQKLEGVLSPSHLEEMEFARSLRKSKVNIKFCQYSYELLLQYLHSTVST---LMLGIINEHINFQVY-
Os_Taf5_NTD2-50-182   ---PARYYDGYSKLRTWAYSSLDQYKHELLRVLYPVFIHSFMDLVAEGHTQEARSFFHTFHEDHELMHSRDLQKLEGILSPSHLEEMELARSLRKNQFRIKLCEYSYELLLQYLQKTQAL---VVLGIINERTTFDVSP
HS_Taf5_NTD2_211-343  ---PTMYEEYYSGLKHFIECSLDCHRAELSQLFYPLFVHMYLELVYNQHENEAKSFFEKFHGDQECYYQDDLRVLSSLTKKEHMKGNETMLDFRTSKFVLRISRDSYQLLKRHLQEKQNN---QIWNIVQEHLYIDIFD
Sc_Taf5_NTD2_149-283  ---PENYIRAYSMLKNWVDSSLEIYKPELSYIMYPIFIYLFLNLVAK-NPVYARRFFDRFSPDFKDFHGSEINRLFSVNSIDHIKENEVASAFQSHKYRITMSKTTLNLLLYFLNENESIGGSLIISVINQHLDPNIVE
Clustal Consensus        *   *   ** *: :   **: :: **  ::**:*:: :::** : :   *: **. *  * :  :  ::. * .: . .*::  *    ::. :  : :.. : :**  .*:..       : .:::::   ::
```

## >Taf5_WD40

```
At_TAF5-WD40-414-663  -----------------------------------------------------------------------------LLGHSGPVYSATFSPPGDFVLSS
Os_TAF5-WD40-402-639  -----------------------------------------------------------------------------FQGHSGPVYSAAFSPFGDFLLSS
Sc_TAF5-WD40-458-722  CMYTFQNTNKDMSCLDFSDDCRIAAAGFQDSYIKIWSLDGSSLNNPNIA--LNNNDKDED------------PTCKTLVGHSGTVYSTSFSPDNKYLLSG
Hs_TAF5-WD40-472-739  ----------GLTAVDVTDDSSLIAGGFADSTVRVWSVTPKKLRSVKQASDLSLIDKESDDVLERIMDEKTASELKILYGHSGPVYGASFSPDRNYLLSS
Clustal Consensus                                                                                   : ****.**.::***  .::**.

At_TAF5-WD40-414-663  SADTTIRLWSTKLNANLVCYKGHNYPVWDAQFSPFGHYFASCSHDRTARIWSMDRIQPLRIMAGHLSDVDCVQWHPNCNYIATGSSDKTVRLWDVQTGEC
Os_TAF5-WD40-402-639  SSDSTIRLWSTKLNANLVCYKGHNYPVWDVQFSPVGHYFASASHDRTARIWSMDKIQPLRIMAGHLSDVDCVQWHVNCNYIATGSSDKTVRLWDVQTGEC
Sc_TAF5-WD40-458-722  SEDKTVRLWSMDTHTALVSYKGHNHPVWDVSFSPLGHYFATASHDQTARLWSCDHIYPLRIFAGHLNDVDCVSFHPNGCYVFTGSSDKTCRMWDVSTGDS
Hs_TAF5-WD40-472-739  SEDGTVRLWSLQTFTCLVGYKGHNYPVWDTQFSPYGYYFVSGGHDRVARLWATDHYQPLRIFAGHLADVNCTRFHPNSNYVATGSADRTVRLWDVLNGNC
Clustal Consensus     * * *:**** .  : ** *****:****..*** *:**.: .**:.**:*: *:  ****:**** **:*. :* *  *: ***:*:* *:*** .*:.

At_TAF5-WD40-414-663  VRIFIGHRSMVLSLAMSPDGRYMASGDEDGTIMMWDLSTARCITPLMGH-NSCVWSLSYSGEGSLLASGSADCTVKLWDVTSSTKLTKAEEKNGNSNRLR
Os_TAF5-WD40-402-639  IRMFIGHRSMVLSLAMSPDGRYMASGDEDGTIMMWDLSSGRCVSPLGGH-SSCVWSLAYSCEGALLASGSADCTVKLWDVASSTKVLKTDDTS--TNRLR
Sc_TAF5-WD40-458-722  VRLFLGHTAPVISIAVCPDGRWLSTGSEDGIINVWDIGTGKRLKQMRGHGKNAIYSLSYSKEGNVLISGGADHTVRVWD---------------------
Hs_TAF5-WD40-472-739  VRIFTGHKGPIHSLTFSPNGRFLATGATDGRVLLWDIGHGLMVGELKGH-TDTVCSLRFSRDGEILASGSMDNTVRLWD---------------------
Clustal Consensus     :*:* ** . : *:..*:**::::*  ** : :**:. .  :  : ** .. : ** :* :* :* **. * **::**

At_TAF5-WD40-414-663  SLRTFPTKSTPVHALRFSRRNLLFAAGA
Os_TAF5-WD40-402-639  MLKTLRTKSTPVYTLRFSRRNLLFAAGA
Sc_TAF5-WD40-458-722  ----------------------------
Hs_TAF5-WD40-472-739  ----------------------------
Clustal Consensus
```

## >Taf6

```
At_TAF6_5-340      PKETVEVIAQSIGITNLLPEAALMLAPDVEYRVREIMQEAIKCMRHSKRTTLTASDVDGALNLRNVEPIYGFASGG----PFRFRKAI--GHRDLFYTDD
Os_TAF6_5-342      PKETIEVIGQSVGIANLPADVSAALAPDVEYRLREIMQEAIKCMRHAKRTVLTADDVDSALSLRNVEPVYGFASGD----PLRFKRAV--GHKDLFYIDD
At_TAF6b-3-327     TKESIEVIAQSIGLSTLSPDVSAALAPDVEYRVREVMQEAIKCMRHARRTTLMAHDVDSALHFRNLEPTSGSKS-------MRFKRAP--ENRDLYFFDD
Sc_TAF6_13-405     PQDTVKDVAESLGLENINDDVLKALAMDVEYRILEIIEQAVKFKRHSKRDVLTTDDVSKALRVLNVEPLYGYYDGSEVNKAVSFSKVNTSGGQSVYYLDE
Hs_TAF6_13-387     PSESMKVVAESMGIAQIQEETCQLLTDEVSYRIKEIAQDALKFMHMGKRQKLTTSDIDYALKLKNVEPLYGFHAQE----FIPFRFAS-GGGRELYFYEE
Clustal Consensus  ..:::: :.:*:*:  :  :.   *: :*.**: *: ::*:*  : .:*  * : *:. ** . *:**  *          . *  .     :.::: ::

At_TAF6_5-340      REVDFKDVIEAPLPKAPLDTEIVCHWLAIEGVQPAIPENAPLEVIRAPA--------------------------ETKIHEQ---------KDGPLIDVR
Os_TAF6_5-342      REVDFKEIIEAPLPKAPLDTAVVAHWLAIEGVQPAIPENPPVDAIVAPT--------------------------ENKRTEHGK-------DDGLPVDIK
At_TAF6b-3-327     KDVELKNVIEAPLPNAPPDASVFLHWLAIDGIQPSIPQNSPLQAISDLK--------------------------RSEYKDD-----------------G
Sc_TAF6_13-405     EEVDFDRLINEPLPQVPRLPTFTTHWLAVEGVQPAIIQNPNLNDIRVSQPPFIRGAIVTALNDNSLQTPVTSTTASASVTDTGASQHLSNVKPGQNTEVK
Hs_TAF6_13-387     KEVDLSDIINTPLPRVPLDVCLKAHWLSIEGCQPAIPENPPPAPKEQQKAEATEPLKSAKPGQEEDGPLKGKGQGATTADGKGKEKKAPPLLEGAPLRLK
Clustal Consensus  .:*::. :*: ***..*    .  ***:::* **:* :*.

At_TAF6_5-340      LPVKHVLSRELQLYFQKIAELAMSKSNP-----PLYKEALVSLASDSGLHPLVPYFTNFIADEVSNG--LNDFRLLFNLMHIVRSLLQNPHIHIEPYLHQ
Os_TAF6_5-342      LPVKHVLSRELQMYFDKIAELTMSRSET-----SVFREALVSLSRDSGLHPLVPYFSYFIADEVTRS--LGDLPVLFALMRVVQSLLHNPHIHIEPYLHQ
At_TAF6b-3-327     LAARQVLSKDLQIYFDKVTEWALTQSGS-----TLFRQALASLEIDPGLHPLVPFFTSFIAEEIVKN--MDNYPILLALMRLARSLLHNPHVHIEPYLHQ
Sc_TAF6_13-405     PLVKHVLSKELQIYFNKVISTLTAKSQADEAAQHMKQAALTSLRTDSGLHQLVPYFIQFIAEQITQN--LSDLQLLTTILEMIYSLLSNTSIFLDPYIHS
Hs_TAF6_13-387     PRSIHELSVEQQLYYKEITEACVGSCEA------KRAEALQSIATDPGLYQMLPRFSTFISEGVRVNVVQNNLALLIYLMRMVKALMDNPTLYLEKYVHE
Clustal Consensus      : ** : *:*:.:: .     . .         ** *:  *.**: ::* *  **:: :  .   .:  :*  ::.:  :*: *. :.:: *:*.
```

```
At_TAF6_5-340      LMPSVVTCLVSRKLGNRFADN-----------HWELRDFAANLVSLICKRYGTVYITLQSRLTRTLVNALLDPKKALTQHYGAIQGLAALGHTVV
Os_TAF6_5-342      LMPSIITCMVAKRLGHRLSDN-----------HWELRDFSANLVGSVCRRFGHAYHNIQTRVTRTLVQGFLDPQKSLTQHYGAIQGISALGPSAI
At_TAF6b-3-327     LMPSIITCLIAKRLGRRSSDN-----------HWDLRNFTASTVASTCKRFGHVYHNLLPRVTRSLLHTFLDPTKALPQHYGAIQGMVALGLNMV
Sc_TAF6_13-405     LMPSILTLLLAKKLGGSPKDDSPQEIHEFLERTNALRDFAASLLDYVLKKFPQAYKSLKPRVTRTLLKTFLDINRVFGTYYGCLKGVSVLEGESI
Hs_TAF6_13-387     LIPAVMTCIVSRQLCLRPDVDN----------HWALRDFAARLVAQICKHFSTTTNNIQSRITKTFTKSWVDEKTPWTTRYGSIAGLAELGHDVI
Clustal Consensus  *:*:::* :::::*      :              **:*:*  :    :::  .  .: .*:*::: :  :*        **.: *:  *    :
```

## >TAF9

```
At_TAF9_10-126     PRDAKIVKSLLKSMGVED--YEPRVIHQFLELWYRYVVEVLTDAQVYSEHASKPN-----IDCDDVKLAIQSKVNFSFSQPPPRE--------------------------VLLELAASRNKIPLPKSIAGPG-VPLPPEQDTLLSPNYQL
Os_TAF9_34-179     PRDARVVRELLRSMGLSEGEYEPRVVHQFLDLAYRYVGDVLGDAQVYADHAGKPQ-----LDADDVRLAIQSKVNFSFSQPPPRECSEFFHSDQDFRSRSLPSDNPLFFSMVLLEVARNRNKIPLPKSIAPPGSIPLPPEQDTLLSQNYQL
Os_TAF9b_27-145    PRDLRVVREILHSLGLREGDYEEAAVHKLLLFAHRYAGDVLGEAKAYAGHAGRES-----LQADDVRLAIQARG-MSSAAPPSRE--------------------------EMLDIAHKCNEIPIPKPCVPSGSISLPHYEDMLLNKKHIF
Hs_TAF9_13-129     PKDAQMMAQILKDMGITE--YEPRVINQMLEFAFRYVTTILDDAKIYSSHAKKAT-----VDADDVRLAIQCRADQSFTSPPPRD--------------------------FLLDIARQRNQTPLPLIKPYSG-PRLPPDRYCLTAPNYRL
Sc_TAF9_30-151     PRDVRLLHLLLASQSIHQ--YEDQVPLQLMDFAHRYTQGVLKDALVYNDYAGSGNSAGSGLGVEDIRLAIAARTQYQFKPTAPKE--------------------------LMLQLAAERNKKALPQVMGTWG-VRLPPEKYCLTAKEWDL
Clustal Consensus  *:* :::  :* . .: :  **  .  ::: : .**.  :* :*  *  :*         :  :*::*** .:   .   ...::                            :*::* . *: .:*      *   **  .  *   :  :
```

## >TAF10

```
At_Taf10_16-119    ----KHEDDAALTEFLASLMDYTPTIPDDLVEHYLAKSGFQCPDVRLIRLVAVATQKFVADVASDALQHCKARPAPVVKDKK--QQKDK----------------------------RLVLTMEDLSKALREYGVNVKHPEYF
Os_Taf10_45-150    ---------AVLTEFLSSLMDYTPTIPDELVEHYLGRSGFYCPDLRLTRLVAVATQKFISDIASDSLQHCKARVAAPIKDNKSKQPKDR----------------------------RLVLTMDDLSKALQEHGVNLKHPEYF
Hs_Taf10_115-217   GDVKPVVSSTPLVDFLMQLEDYTPTIPDAVTGYYLNRAGFEASDPRIIRLISLAAQKFISDIANDALQHCKMKGTASGSSRS--KSKDR----------------------------KYTLTMEDLTPALSEYGINVKKPHYF
Sc_Taf10_72-205    ---------KTLEEILEMMDSTPPIIPDAVIDYYLTKNGFNVADVRVKRLLALATQKFVSDIAKDAYEYSRIRSSVAVSNANNSQARARQLLQGQQQPGVQQISQQQHQQNEKTTASKVVLTVNDLSSAVAEYGLNIGRPDFY
Clustal Consensus             * ::*  : . .* *** :  :** : **  .* *: **:::*:***::*:*.*: ::.: : :   .. .  : : :                            : .**::**: *: *:*:*: :*.::
```

## >TAF12

```
Hs_Taf12_57-128    ------------------VLTKKKLQDLVREVDPNEQ-----LDEDVEEMLLQIADDFIESVVTAACQLARHRKSSTLEVKDVQLHLERQWNMWI
Sc_Taf12_414-490   --------LPPYEMDTQRVMSKRKLRELVKTVGIDEGDGETVIDGDVEELLLDLADDFVTNVTAFSCRLAKHRKSDNLEARDIQLHLERNWNIRI
At_taf12b_527_598  ------------TEATNQLLGKRKIQDLVSQVDVHAK-----LDPDVEDLLLEVADDFIDSVTSFACSLAKHRKSSVLEPKDILLHLEKNLHLTI
Os_taf12b_400-471  -------------------LGKRKIQDLVSQVDPLGK-----VDPEVEDLLLEIADDFIDSVTAFACTLAKHRKSSVLEAKDVLLHLEKNWHLSV
At_Taf12_399-469   PGNHAKTVSAETEPSDDRILGKRSIHELLQQIDPSEK-----LDPEVEDILSDIAEDFVESITTFGCSLAKHRKSDILEAKDIL-----------
Os_Taf12_169-240   ------------------LLSKRSIHELVAQIDPSEK-----LDPEVEDVLIDIAEDFVESVATFACSLAKHRKSSILEAKDVLLHAERSWNITL
Clustal Consensus                     : *:.:::*:  :.         :* :**::* ::*:**: .:.: .* **:****. ** :*:
```

## >TRA-FAT

```
AtTRA1b_FAT_2776-3116  YEILLDSLWKAPDWTYLK---DHVIPKAQVEETPKLRLVQACFSLHEKNANGVGDAEN--IVGKGVDLALEQWWQLPEMSLHARVPLLQQFQQLVEVQES
AtTRA1a_FAT_2785-3125  YEILLDSLWKLPDWAYLK---DHVIPKAQVEETPKLRLVQSYFALHDRNSNGVGDAEN--TVGKGVDLALEQWWQLPEMSVHARVPLLQQFQQLVEVQES
Os_FAT_1-98            ----------------------------------------------------------------------------------------------------
Sc_FAT_2757-3106       -DLLLECGWRVADWNSDRDALEQSVKSVMDVPTPRRQMFKTFLALQNFAESRKGDQEVRKLCDEGIQLSLIKWVSLPIRYTPAHKWLLHGFQQYMEFLEA
Hs_FAT_2833-3175       --LVLECAWRVSNWTAMKEALVQVEVSCPKEMAWKVNMYRGYLAICHPEEQQLSFIER--LVEMASSLAIREWRRLPHVVSHVHTPLLQAAQQIIELQEA
Clustal Consensus


AtTRA1b_FAT_2776-3116  SRIYVDIANGSKVPGNAAVGGQGNLYADLKDILETWRLRTPNEWDNMTVWYDMLQWRNEMYNVVIDAFKDFVTSNTPLH---------HLGYRDKAWNVN
AtTRA1a_FAT_2785-3125  ARIHVDIANGNKVSGNTAVGGLGNRYADLKDILETWRLRTPNEWDNMTVWYDMLQWRNEMYNVVIDAFKDFATSNSPLH---------HLGFRDKAWNVN
Os_FAT_1-98            ----------------------------------------------------------------------------------------------------
Sc_FAT_2757-3106       TQIYANLHT-------TTVQNLDSKAQEIKRILQAWRDRLPNTWDDVNMWNDLVTWRQHAFQVINNAYLPLIPALQQSNSNSNINTHAYRGYHEIAWVIN
Hs_FAT_2833-3175       AQINAGLQP--------TNLGRNNSLHDMKTVVKTWRNRLPIVSDDLSHWSSIFMWRQHHYQAIVTAYENSSQHDPSSN-------NAMLGVHASASAII
Clustal Consensus

AtTRA1b_FAT_2776-3116  KLARIARKQGLYDVCVQILEKMYGHSQMEVQEAFVKIKEQAKAHLETK-----GELATGLNLVNSTNLEFFLAKNKAEIFRLKGDFHLKLNDTEGANLAY
AtTRA1a_FAT_2785-3125  KLARIARKQGLYDVCVQILEKMYGHSTMEVQEAFVKIREQAKAYLEMK-----GERASGLNLINSTNLEYFPDKIKAEIFRLKGDFHLKLNDTESANIAY
Os_FAT_1-98            --------------------------------------------------------------INNTNLEFFPVKNKAEIFRLRGDFLLKMNDCENANVAY
Sc_FAT_2757-3106       RFAHVARKHNMPDVCISQLARIYTLPNIEIQEAFLKLREQAKCHYQNM-----NELTTGLDVISNTNLVYFGTVQKAEFFTLKGMFLSKLRAYEEANQAF
Hs_FAT_2833-3175       QYGKIARKQGLVNVALDILSRIHTIPTVPIVDCFQKIRQQVKCYLQLAGVMGKNECMQGLEVIESTNLKYFTKEMTAEFYALKGMFLAQINKSEEANKAF
Clustal Consensus                                                                    :..*** :*    .**:: *:* *  ::.  * ** *:

AtTRA1b_FAT_2776-3116  SNAITLFKNLPKGWISWGNYCDMAYQDTQDEIWLEY-AVSCFLQGIRFG-VSNSRSHMARVL-
AtTRA1a_FAT_2785-3125  SNAITLFKNLPKGWISWGSYCDMAYQETQEEIWLEY-AVSCFLQGIRFG-VSNSRSHIARVL-
Os_FAT_1-98            SNAITLFKHLPKAWISWGNYCDMVFKETKDEIWLEY-AVSCFFQGIKYG-VSNSRSHLARIL-
Sc_FAT_2757-3106       ATAVQIDLNLAKAWAQWGFFNDRRLSEEPNNISFASNAISCYLQAAGLYKNSKIRELLCRILW
Hs_FAT_2833-3175       SAAVQMHDVLVKAWAMWGDYLENIFVKER-QLHLGVSAITCYLHACRHQNESKSRKYLAKVLW
Clustal Consensus      : *: :   * *.*  ** : :    .   :: :   *::*:::.      *: *. :.::*
```

## >TRA-FATc

```
At-Tra1a-Fatc-3827-3858  -PQSVQRGVNELVEAALSPRNLCMMDPTWHPWF
At-Tra1b-Fatc-3803-3834  -PQSVQRGVSELVEAALSPRNLCMMDPTWHPWF
Os-Tra1-Fatc-811-842     -PQSVQRGVTDLVEAALSSRNLCMMDPTWHPWF
Hs-TRRAP-Fatc-3803-3830  -----ESKVNTLVAAANSLDNLCRMDPAWHPWL
Sc-Tra1-Fatc-3712-3744   TPTVTTQFILDCIGSAVSPRNLARTDVNFMPWF
Clustal Consensus                :   : :* *  **.  *  : **:
```

## >TRA-TRRAP

```
AtTRA1b_TRRAP-3458-3746  LDRVGADIQIVRRHGSSCRRLTLIGSDGSQKHFIVQTSLTPN-ARSDERILQLFRVMNQMFDKHKESRRRHLGLHTPIIIPVWSQVRMVEDDLMYNTFLE
AtTRA1a_TRRAP-3482-3770  LDRVGADVPIVRRHGSSFRRLTLIGSDGSQKHFIVQTSLTPN-ARSDERILQLFRVMNQMFDKHKESRRRHIGIHTPIIIPVWSQVRMVEDDLMYNTFLE
```

```
OsTRA1_TRRAP-467-756     LDRVGPDIPIVRRHGSSFRRLTLIGSDGSQRHFIVQTSLTPN-ARSDERMLQLFRVLNKMFDKHKESRQRHLAIHTPIIIPVWSQVRMVEDDLMYSTFLE
Sc_TRRAP_3371-3676       IARFLPTVDFVRGTHSSYRRLMIRGHDGSVHSFAVQYPAVRH-SRREERMFQLYRLFNKSLSKNVETRRRSIQFNLPIAIPLSPQVRIMNDSVSFTTLHE
Hs_TRRAP_3467-3754       IARFMPRVEIVQKHNTAARRLYIRGHNGKIYPYLVMNDACLTESRREERVLQLLRLLNPCLEKRKETTKRHLFFTVPRVVAVSPQMRLVEDNPSSLSLVE
Clustal Consensus        : *. . : :*:   :: *** : * :*.   : *        :* :**::** *::*  :.*. *: :* : :  *  :.: .*:*:::*.    :: *

AtTRA1b_TRRAP-3458-3746  VYENHCGRNGRESDLPITYFKEKLNQAITGQISPEAIGDLRLQAYGEITKNIVNDTIFSQYMYKTSMSGSHLWAFKKQFAVQLAVSNFMSFILQIGGRSP
AtTRA1a_TRRAP-3482-3770  VYENHCARNDREADLPITHFKEQLNQAISGQISAEAIGDLRLQAYIDITKTLVNDSIFSQYMYKTLMSGSHMWAFKKQFAVQLAVSSFMSFMLQIGGRSP
OsTRA1_TRRAP-467-756     VYEINCARHNREADSPITIFKEQLNQAISGQVSPEAVVELRLQAYNEITKNIVNDNIFSQYMHKILPTGNHLWTFKKQFAIQVALSCFMSYMLQIGGRAP
Sc_TRRAP_3371-3676       IHNEFCKKKGFDPDDIQDFMADKLNAAHDDALPAPDMTILKVEIFNSIQTMFVPSNVLKDHFTSLFTQFEDFWLFRKQFASQYSSFVFMSYMMMINNRTP
Hs_TRRAP_3467-3754       IYKQRCAKKGIEHDNPISRYYDRLATVQARGTQASHQVLR--DILKEVQSNMVPRSMLKEWALHTFPNATDYWTFRKMFTIQLALIGFAEFVLHLNRLNP
Clustal Consensus        :::  * ::. : *       ::*  .      .        :   .: . :*  .::.:           . * *:* *: * :   * .::: :.   *

AtTRA1b_TRRAP-3458-3746  NKILFAKNSGKMFQTDFHPSYDSNG-----------------MIELNEPVPFRLTRNMHAFLSHFGVEGPLMSNMCSASQAVFSSKQNEHLRYQLAMFF
AtTRA1a_TRRAP-3482-3770  NKVLFAKNTGKMFQTDFHPAYDANG-----------------MIEFNEPVPFRLTRNMQAFFSQFGVEGLLMSSMCSAAQAVISSKQNEHLRYQLAMFF
OsTRA1_TRRAP-467-756     NKILFAKNTGKIFQNDFHPAYDPNG-----------------MIEFNELVPFRLTRNMQAFFSNFGVEGLIVSAMCSAAQSVVSPKQSQHIWHHLAMFF
Sc_TRRAP_3371-3676       HKIHVDKTSGNVFTLEMLPSRFPYERVKPLLKNHDLSLPPDSPIFHNNEPVPFRLTPNIQSLIGDSALEGIFAVNLFTISRALIEP--DNELNTYLALFI
Hs_TRRAP_3467-3754       EMLQIAQDTGKLNVAYFRFDINDATG----------------DLDANRPVPFRLTPNISEFLTTIGVSGPLTASMIAVARCFAQP--NFKVDGILKTVL
Clustal Consensus        . : . : :*::    :                          :. *. ****** *:  ::   .:.* :   : : ::.. ..  . .:   *  .:

AtTRA1b_TRRAP-3458-3746  RDELLSWF-
AtTRA1a_TRRAP-3482-3770  RDELLSWF-
OsTRA1a_TRRAP-467-756    RDELLSWSW
Sc_TRRAP_3371-3676       RDEIISWFS
Hs_TRRAP_3467-3754       RDEIIAWHK
Clustal Consensus        ***:::*
```

## >SGF29

```
At_sgf29a_131-266  ASLKGEQVAARVTAESADKDEWFVVKVIHFDRETKEVEVLDEEPGDDEEGSGQRTYKLPMLCILPFPKRNDPSNTQEFPPGKHVLAVYPGTTALYKATVVSTPRKRKSDEYLLEFDDDEEDGALPQRTVPFHKVVALPE----
At_sgf29b_134-269  --LKGEQVAARVTAEDAEKDEWFVVKVIHFDRETKEVEVLDEEPGDDEEGGGQRTYKLSMSCILPFPKRNDPSSTQEFIPGKHVLAVYPGTTALYKATVISTPRKRKSDEYLLEFDDDEEDGALPQRTVPFHKVVALPEGHRQ
Os_sgf29_199-332   --LKGEQVAAKVKSD-EEKDEWFVVKVIHFDKETKEYEVLDEEPGDDEE-SAQKKYKLPMSDIIPFPKRGDPSSAPDFGQGRQVLAVYPSTTALYRATVASN-RKRKSDDYLLLFDDDEEDGNLPQRAVPFYRVVPLPE----
Hs_Sgf29_158-289   ---PGDKVAARVKAVDGDEQ-WILAEVVSYSHATNKYEVDDIDEEGKERHTLSRRRVIPLPQWKANP---ETDPEALFQKEQLVLALYPQTTCFYRALIHAPPQRPQDDYSVLFEDTSYADGYSPPLNVAQRYVVACKE----
Sc_Sgf29_126-250   --LVGSEVAYKPRRG-SADGEWIQCEVLKVVADGTRFEVRDPEP--DELGNSGKVYKCNRKELLLIP---PGFPTKNYPPGTKVLARYPETTTFYPAIVIGT---KRDGTCRLRFDGEEE--VDKETEVTRRLVLPSP-----
Clustal Consensus     *.:** :        .  *:  :*:       .. ** * :   .*     :              *          :     *** ** ** :* * : .     :..   *  * .           *.   *:.
```

## >UBP8

```
AT_UBP22_177-528   ----TFPLGLRGLNNLGSTCFMNAVLQALVHAPPLRNFWLSGQHNRDLCPRRT-MG---------LLCLPCDLDVIFSAMFSGDRTPYSP-----------AHLLYSWWQHSTNLATYEQQDSHEFFISLLDRIHE--NEGKSKCLYQ---DNEECQCITHKAFSGLLRSDVTCTTCGSTS-TTYDPFIDISLTLDS--
OS_UBP22_211-576   SASAAAPAGLRGLNNLGNTCFMNSVLQALLHAPPLRNYFLGDRHNRYLCPRQTPMRRRSAEANDKAACLACDLDEIYSAAFSGERTPYSP-----------AKFLYSWWQHASNLASYEQQDAHEFFISILDHIHENIKDDQHKSLAQ---GHGDC-CIAHRVFSGILRSDVTCTHCGFTS-TTFEPCMDISLDLDAGY
HS_USP22_176-518   ----------RGLINLGNTCFMNCIVQALTHTPLLRDFFLSDRHR---CEMQSPSS----------CLVCEMSSLFQEFYSGHRSPHIP-----------YKLLHLVWTHARHLAGYEQQDAHEFLIAALDVLHRHCKGDDNGKKAN---NPNHCNCIIDQIFTGGLQSDVTCQVCHGVS-TTIDPFWDISLDLPGSS
SC_UBP8p_138-466   -----------GLINMGSTCFMSSILQCLIHNPYFIRHSMSQIHSNN-CKVRSPDK----------CFSCALDKIVHELYGALNTKQASSSSTSTNRQTGFIYLLTCAWKINQNLAGYSQQDAHEFWQFIINQIHQSYVLDLPNAKEVSRANNKQCECIVHTVFEGSLESSIVCPGCQNNSKTTIDPFLDLSLDIKD--
Clustal Consensus             ** *:*.****..::*.* * * :  . :.  *    *  ::                *: * :. :    :.. .:   .           :*   *    :** *.***:***    :: :*.     .           .  .* ** .  * * *.*.:.*  *   * ** :*  *:** :

AT_UBP22_177-528   MNGFSPADCRKNRYSGGPS---VNAIMPTLSGCLDFFTRSEKLGPDQKLNCQSCGEKRESSKQMSIRRLPLLLCLHVKRFEHSLTRKTSRKIDSYLQYPFRLNMSPYLSSSIIGKRFGNRIFAFDGEGEYDSSSSSSPSAEFEIFAVVTHKGMLESGHYVTYLR-LKGLWYRCDDAWINEVEEEVVRGCECYMLFY-
OS_UBP22_211-576   NNSLGVANPKVHVRNGERSSGGTNTKVSTLMRCLERFTRAERLDAEQKFFCERCKERQESLKQMSIRRLPLVSCFHIKRFEHSSVKKMSRKVDHCLQFPFSLDMAPYLSSSILRSRFGNRIFPSEAS---DADSVSEFSSEFEIFAVIMHSGKLEAGHYVTYLR-LNNHWYKCDDAWVTRVEEHTVRTSQAYMLFYV
HS_USP22_176-518   TPFWPLSPGSEGNVVNGES---HVSGTTTLTDCLRRFTRPEHLGSSAKIKCSGCHSYQESTKQLTMKKLPIVACFHLKRFEHS--AKLRRKITTYVSFPLELDMTPFMASSKESRMNGQYQQP--------TDSLNN-DNKYSLFAVVNHQGTLESGHYTSFIRQHKDQWFKCDDAIITKASIKDVLDSEGYLLFYH
SC_UBP8p_138-466   ------------------------KKKLYECLDSFHKKEQLK-DFNYHCGECNSTQDAIKQLGIHKLPSVLVLQLKRFEHLLNGSNR-KLDDFIEFPTYLNMKNYCSTKEKDKHSENGKVP--------------DIIYELIGIVSHKGTVNEGHYIAFCKISGGQWFKFNDSMVSSISQEEVLKEQAYLLFYT
Clustal Consensus                                .*   **   * : *:*   . :   *   *  . ::: **: :::** :  :::*****    .   *:   :.:*  *:*  : ::.       :    .               .  :.::.:: *.* :: *** :: :    . *:: :*: :.   . . *    : *:***
```

## >SGF11

```
At_Sgf11-89-121    ASEVFNCMNCGRQIVAGRFAPHLEKCMGKGRKA
Os_Sgf11-93-125    AADLVDCMNCGRPVAAGRFAPHLEKCMGKGRKA
Hs_Sgf11-80-112    KSKECVCPNCSRSIAASRFAPHLEKCLGMGRNS
Sc_Sgf11-67-99     SSQYIHCENCGRDVSANRLAAHLQRCLSRGARR
Clustal Consensus   :.    * **.* : *.*:*.**::*:. * .
```

## >ENY2

```
At_eny2_26-110     EKDKVTLREIINVKLVESGEKENLMELVRDRLVECGWKDEMRIACREHVKKKGRKDVTVDELIRVITPKGRASVPDSVKAELLNRIQNFI-
Os_eny2_22-107     -----SLREIINVKLVESGEKEKLMELLRERLVECGWRDEMKALCRAYARKKGRNNVTVDDLIHVITPKGRASVPDSVKAELLQRIQSFLM
Hs_eny2_11-95      -----QMRAAINQKLIETGERERLKELLRAKLIECGWKDQLKAHCKEVIKEKGLEHVTVDDLVAEITPKGRALVPDSVKKELLQRIRTFL-
Sc_eny2_7-90       -----QMRAAINQKLIETGERERLKELLRAKLIECGWKDQLKAHCKEVIKEKGLEHVTVDDLVAEITPKGRALVPDSVKKELLQRIRTFL-
Clustal Consensus       :*  ** **:*:**:*.* **:* :*:****:*:::  *:   ::** :.****:*:  ******* ****** ***:**:.*:
```

## >CHD1_chromo

```
At_chromo_535-588  QNSQVERIIADRI-SKDGLGD-------------VVPEYLVKWQGLSYAEATWEKDVD-IAFAQ-VAIDEYKAR
Os_chromo_507-560  ----VERIFADRV-SKVDGDD-------------LVPEYLVKWQGLPYAESTWEKDTD-IEFAQ-DAIDEYKAR
Sc_chromo_286-341  ---VPERIIDSQR-ASLEDGT-------------SQLQYLVKWRRLNYDEATWENATDIVKLAP-EQVKHFQNR
Hs_chromo_378-447  --QIVERVIAVKT-SKSTLGQTDFPAHSRKPAPSNEPEYLCKWMGLPYSECSWEDEALIGKKFQ-NCIDSFHSR
Hs_chromo_300-325  ----------------KDEGE---------------IQYLIKWKGWSYIHSTWESEE-----------------
At-chromo_460-499  ----------------------------------NEMEFLIKWKGQSHLHCQWKTLSDLQNLSGFKKVLNYTKK
Os_chromo_433-471  ----------------------------------DVEFYIKWKGQSFLHCQWKTLSELQNVSGFKKVLNYMKR
Sc_chromo_176-247  -FHGIDIVINHRLKTSLEEGKVLEKTVPDLNNCKENYEFLIKWTDESHLHNTWETYESIGQVRGLKRLDNYCK-
Clustal Consensus                                      :::  **    . .  *:
```

## >CHD1_helic

```
At_HELICc_937-1061  SSGKLVILDKLLVRLRETKHRVLIFSQMVRMLDILAEYLSLRGFQFQRLDGSTKAELRQQAMDHFNAPASDDFCFLLSTRAGGLGINLATADTVVIFDSDWNPQNDLQAMSRAHRIGQQEVVNIY
Os_HELICc_926-1034  ----------------ETNHRVLIFSQMVRMLDILAEYLSLRGFQFQRLDGSTRADLRHQAMEHFNAPGSDDFCFLLSTRAGGLGINLATADTVIIFDSDWNPQNDLQAMSRAHRIGQQETVNIY
Hs_HELICc_807-913   ------------------GNRVLIFSQMVRMLDILAEYLTIKHYPFQRLDGSIKGEIRKQALDHFNADGSEDFCFLLSTRAGGLGINLASADTVVIFDSDWNPQNDLQAQARAHRIGQKKQVNIY
Sc_HELICc_693-817   -SGKMVLLDQLLTRLKKDGHRVLIFSQMVRMLDILGDYLSIKGINFQRLDGTVPSAQRRISIDHFNSPDSNDFVFLLSTRAGGLGINLMTADTVVIFDSDWNPQADLQAMARAHRIGQKNHVMVY
Clustal Consensus                      :***************.:**:::   ******:  .  *: :::***:  *:** ************** :****:********* **** :*******:: * :*
```

## >CHD1-dexdc

```
Hs_DEXDc_503-648   ---NSVILADEMGLGKTIQTISFLSYLFHQHQLYGPFLIVVPLSTLTSWQREFEIWAPEINVVVYIGDLMSRNTIREYEWIHS----QTKRLKFNALITTYEILLKDKTVLGSINWAFLGVDEAHRLKNDDSLLYKTLIDFKSNHRLLITGTP
Sc_DEXDc_396-544   ----NGILADEMGLGKTVQTVAFISWLIFARRQNGPHIIVVPLSTMPAWLDTFEKWAPDLNCICYMGNQKSRDTIREYEFYTNPRAKGKKTMKFNVLLTTYEYILKDRAELGSIKWQFMAVDEAHRLKNAESSLYESLNSFKVANRMLITGTP
At_DEXDc_645-791   LNDTNVILADEMGLGKTVQSVSMLGFLQNTQQIPGPFLVVVPLSTLANWAKEFRKWLPGMNIIVYVGTRASREVCQQYEFYNE--KKVGRPIKFNALLTTYEVVLKDKAVLSKIKWIYLMVDEAHRLKNSEAQLYTALLEFSTKNKLLITGTP
Os_DEXDc_617-763   ----NVILADEMGLGKTIQSVSMLGFLHNAQEINGPFLVVVPLSTLSNWAKEFRKWLPDMNVVVYVGNRASREICQQHEFFTN--KKGGRHVKFHTLITTYEVILKDKAALSKIKWNYLMVDEAHRLKNCEASLYTTLLEFSTKNKLLITGTP
Clustal Consensus      . ***********:*:::::.:*   :.  **.::******:. *   *. * * :* : *:*   **:  :::*:  .      : :**:.*:**** :***:: *..*:* :: ********* :: ** :* .*.  :::******
```

## >ADA2b_SWIRM

```
At_SWIRM_409-483   ---LDLIGFTESQLLSESEKRLCSEVKLVPPVYLQMQQVMSHEIFK-GNVTKKSDAYSLFKIDPTKVDRVYDMLVKK-GI---
Os_SWIRM_485-553   ---WDIVGFPGAELLSTSEKNLCCQNRLLPNHYLKMQEVLMQEIFK-GSVAKKEDAHVLFKVDPAKVDNVYDMVTKKLGTNEE
Sc_SWIRM_355_428   MTISDIQHAPDYALLSNDEQQLCIQLKILPKPYLVLKEVMFRELLKTGGNLSKSACRELLNIDPIKANRIYDFF---------
Hs_SWIRM_364-440   --PLNLTGLPGTEKLNEKEKELCQMVRLVPGAYLEYKSALLNECNKQGGLR-LAQARALIKIDVNKTRKIYDFLIREGYI---
Clustal Consensus      ::   .    *. .*:.**   :::*  **  :..: .*  * *.      .  *:::*  *. .:**:.
```

## > ADA2b_SANT

```
At_SANT_105-147    ICPDWSADDEMLLLEGLEIYGLGNWAEVAEHVGTKSKEQCLEHYRNI-
Os_SANT_111-152    ----WNADEEILLLEGIEMYGLGNWAEVAEHVGTKTKAQCIDHYTT--
Hs_SANT_74-118     ---SWTAQEEMALLEAVMDCGFGNWQDVANQMCTKTKEECEKHYMKHF
Sc_SANT_65-107     ----WGADEELQLIKGAQTLGLGNWQDIADHIGSRGKEEVKEHYLKY-
Clustal Consensus      * *::*: *::.    *:*** ::*::: :: * :  .** .
```

## >ADA2b_ZZ-ADA2

```
At_ZZ_ADA2_45-93   GGGKYNCDYCQKDITGKIRIKCAVC--PDFDLCIECMSVGAEITPHKCDHPYRVM
Os_ZZ_ADA2_51-99   ----YHCNYCNKDISGKIRIKCSKC--PDFDLCVECFSVGAEVTPHRSNHPYRVM
Sc_ZZ_ADA2_5-53    ----FHCDVCSADCTNRVRVSCAIC--PEYDLCVPCFSQGSYTGKHRPYHDYRII
Hs_ZZ_ADA2_15-64   ----PPCRGCSSYLMEPY-IKCAECGPPPFFLCLQCFTRGFEYKKHQSDHTYEIM
Clustal Consensus        *  *.      :.*: *  * : **: *:: *     *:  * *.::
```

## >Spt3_TAF13

```
atspt3             KTLFQKELQHMMYGFGDEQN--------------------PLPESVALVEDIVVEYVTDLTHKAQEIGSKRGR--LLVDDFLYLIRKDLPKLNRCRELLAMQEELKQARKAFDVD
osspt3             RGVFQKDLQHMMYGFGDDPNARNCLGIGCNLCTLCCGTLEPLPETVALVEDIVVEYVTDLVHKAQNVASKRGK--LLTEDFLYLIRKDVRKLHRATELLSMNEELKQARKAFD--
Hsspt3             ---FATELQSMMYSLGDARR--------------------PLHETAVLVEDVVHTQLINLLQQAAEVSQLRGARVITPEDLLFLMRKDKKKLRRLLKYMFIRDYKSKIVKGID--
Scspt3             KHKYRVEIQQMMFVSGEIND--------------------PPVETTSLIEDIVRGQVIEILLQSNKTAHLRGSRSILPEDVIFLIRHDKAKVNRLRTYLSWKDLRKNAKDQD---
Clustal Consensus     :  ::* **:  *:                       *  *:. *:**:*   :  ::  :: : .  **   :  :*.::*:*:*  *:.*    :  .:  .:  .
```

## >Spt20_spt20

```
At_spt20-62-208    ----------DHEVSFILSLYPNGYSIG-------KTSEAMQQISFRDVPKVLHPYDRAAEGLLSAIEAGRLPGDILEDIP----CKFVDGVVICEVHDY
Os_spt20-76-236    ---------LDLEPSFALNLFPDGYSISDPGKRLLVSAKGMLLFLIGDDPQK-RPYSKASRALFSDIEHGCLPQVILGDMP----CKFRNGTIVCEVRDY
Hs_spt20-74-226    ------------LSCLVVNLYPGNEGYS----------LMLRGKNGSDSETIRLPYEEG--ELLEYLDAEELPPILVDLLEKSQVNIFHCGCVIAEIRDY
Sc_spt20-122-341   DADSILKKYEQYLHSFEFHIYENNYKICAPAN--SRLQQQQKQPELTSDGLILTKNNETLKEFLEYVARGRIPDAIMEVLRDCN-IQFYEGNLILQVYDH
Clustal Consensus               .: . :: ..                      .        ..    ::. :   :*  ::  :       *  * :: :: *:

At_spt20-62-208    R-------------------------------------------KHTSSQVS------PVINKLRLKMSLENVVKDIPSMS--DNSWTYGDLMEVESRI
Os_spt20-76-236    R-------------------------------------------PFLSNAGDSSGDDFPIVNRVSLRLGTERVVKDLASVV--NASWTYHDQLIAESTI
Hs_spt20-74-226    R-------------------------------------------QSSNMKSPG-----YQSRHILLRPTMQTLICDVHSITSDNHKWTQEDKLLLESQL
Sc_spt20-122-341   TNTVDVTPKENKPNLNSSSSPSNNNSTQDNSKIQQPSEPNSGVANTGANTANKKASFKRPRVYRTLLKPNDLTTYYDMMSYAD-NARFSDSIYQQFESEI
Clustal Consensus                                                .              : *:        *: *    :  ::      ** :

At_spt20-62-208    LKALQPELCLDPLPRLDRLS------
Os_spt20-76-236    LRALQPRLNLDPTPCLERLQN-----
Hs_spt20-74-226    ILATAEPLCLDPSIAVTCTANRLLYN
Sc_spt20-122-341   LTLTKRNLSLSVPLNPYEHRDMLE--
Clustal Consensus  :      * *.
```

## >ADA3-ada3

```
At-ADA3-835-957    --ETLGIDEKIYMEAQSIGICLDPMPS-----------ISNVEDEGIVDDIKTLEEAICEVVSKKKDMLNRLLKPALEMKERQEKEFERLGYEKLIEMAY
Os-ADA3-889-995    ------FSDRIIIELSEVGVSIEPVPD-----------LAQSEDEDINSEICKLEGQLHKEVVDKKNLLRKLDGILRTKKESQHREFSRRAMERLLLIAY
Sc-ADA3-505-649    NLDYPTFEERLKRELKYVGIYMNLPKDENNPNSDDPDWVTGREDDEISAELRELQGTLKQVTKKNQKRKAQLIPLVERQLAWQEYSSILEDLDKQIDQAY
Hs-ADA3-309-431    -PHTKSLESRIKEELIAQGLLESEDRP------------AEDSEDEVLAELRKRQAELKALSAHNRTKKHDLLRLAKEEVSRQELRQRVRMADNEVMDAF
Clustal Consensus        :..::  *    *:  .                :  .:: :  ::  :  :     .::    *          *.        :. :  *:

At-ADA3-835-957    EKSK----ASRRHHSASGK-----SSATKISKQAAFAFVKRTLER
Os-ADA3-889-995    EK----------YMAFCGS-----SSSKNVNRAGKHAAL------
```

```
Sc-ADA3-505-649     VKRIRVPKKRKKHHTAASNNVNTGTTSQIAQQKAANSSLKSLLDK
Hs-ADA3-309-431     RKIMA---ARQKKRTPTKK-----EKDQAWKTLKERESILKLLD-
Clustal Consensus    *            :   .      .    .       :
```

## >ADA1-TAD1

```
Os-ada1a-6-270      -AKKVAARVDTMEIKSQIAKKLGAERSEHYFHSLKKFLGGQLGKEEFDKICVATMG--------------RDNIKYHNFLIRSILSNAYLATAPPPPPPP
Os-ada1b-3-236      -TSRKLARVDIAELKQRLVKRLGRQRAGQYFAHLTRLLNLKLTKVEFDKLCYATIG--------------RENIALHNALIRGIISNALSG-----VPPP
At-ada1a-3-248      -SDQCFSRLNSLEIKALIYQKIGHQRADTYFDQLGKFLTSRISKSEFDKLCSKTVG--------------RENISLHNRLVRSILKNASVAK-----SPP
At-ada1b-3-243      TSQHHVVRTDISELKSQIEKRIGRAKTESYLNLLSKFLSLKISKSDFDKLIIVTVK--------------RENISLHNALLRGILKNICLSKT---LPPF
Hs-ada1-7-195       ---------ELEAAKKNLSEALG-DNVKQYWANLKLWFKQKISKEEFDLEAHRLLT--------------QDNVHSHNDFLLAILT-------------R
Sc-ada1-54-317      -PQGPNQRIDLGAMIEELTSLLGKESWTKYAQIISLFILGKLSRKELSNELELVFSPSAASLEKSNTNHHHSLVRLHNQLLLGIFANSLRENPLGRNGNE
Clustal Consensus            :       : . :*      *   :   :  :: : ::.      .               :. :  ** :: .*:

Os-ada1a-6-270      SRQATTGNSQTSTVSVSNGAVANHGVMAGVMRGPALATREARFERPSPLGKSPLGHQGTGEFVSAGSKAPLEVVSVEDG-----EEVNQAGGSPVYAQSR
Os-ada1b-3-236      SRQAVTG--QSGTTTAPSGQCVGIALQSARNVGAVVDSGDGDFAR---------------ERAVAG-----KVLSVEDG-----EEVEQVRSAP-CVQSR
At-ada1a-3-248      PRYPKKSLYGDPVFPPSPRKCRSRKFRDRPSPLGPLGKPQSLTTTND-------------ESMSKAQRLPMEVVSVEDG-----EEVEQMTGSP-SVQSR
At-ada1b-3-243      VKNGVESDNKKKKQLNGAFQSLCKELPRSPRKGRTQRRLNK------------------DGNISKGKSLVTEVVSSSGRQQWSMENVEEVDQLIPCWRSQ
Hs-ada1-7-195       CQILVSTPDGAGSLPWPGGSAAKPGKPKGKKKLSSVR-----------------------------------------------QKFDHR------FQPQ
Sc-ada1-54-317      SSWGFGNGSNNPNNKLKRINKHNSQIEVYKKIVMSLPLNDR----------------NRLKMITKEAGKRGFIFCSVFQ-----ARLNNIPKIP-IVTNP
Clustal Consensus                                                                                        ..:.

Os-ada1a-6-270      SPIRAPLGVSFGDPKAQNSRPSIPHPSLICYKNGELPEAQRLLKLLENKLQAEGLS--LTQECADVLNSGLNAYLSRLLKSCMGVAK
Os-ada1b-3-236      SPITAPLGIST-TPTYGARTWRLDDPMVSCYDSHHLLDTGSLFKGLQRRLESDGIG--VSVQGVEVLNRGLDEFLRRLIKPCMEL--
At-ada1a-3-248      SPLTAPLGVSFHLKSKARFSTYNGINRETCQSSGELPDMITLRARLEKKLEMEGIK--LSMDSANLLNRGLNAYMRRLIEPCLSLAS
At-ada1b-3-243      -PIEAPFGVNLRDVIKKQHR-----IDTCCYSSGELPDSVSLKKKLEDDLE-EGLE--VSVGFANSLNAGLDVFLKRLIKPCLEL--
Hs-ada1-7-195       NPLSGAQQFVAKDPQD-------DDDLKLCSHTMMLPTRGQLEGRMIVTAYEHGLDN-VTEEAVSAVVYAVENHLKDILTSVVSRRK
Sc-ada1-54-317      ESLKRVKSNNLKTPLEWSQDIMNGFNVPLASESHSLPDTDSFYLRMVGIAREHGLVGTVDARCVELISLALDQYLKNIIEFTIDTVR
Clustal Consensus    .:                          .  .  *     :   :      .*:   :    .. :  .:: .:  ::   :
```

**S1 Fig: Domain similarity between human, *S. cerevisiae*, *Arabidopsis* and *O. sativa* SAGA complex encoding genes.**

Sequence alignments of the representative domains of each protein of the SAGA complex were done by using Clustal X.
