# Supplementary material for: Spt-Ada-Gcn5-Acetyltransferase (SAGA) Complex in Plants: Genome Wide Identification, Evolutionary Conservation and Functional Determination
Source: PLoS One. 2015 Aug 11;10(8):e0134709. doi: 10.1371/journal.pone.0134709 (PMC4532415; doi:10.1371/journal.pone.0134709)
Supplement: S4 Fig — SAGA encoding genes are plotted on the five Arabidopsis chromosomes according to their sequence spots. The chromosome number is shown at the top of each chromosome and the centromeric regions by constriction on chromosome line bar. Each identical duplicated chromosomal segment is marked by same line colour. The scale is in mega bases (Mb). (PDF) [file pone.0134709.s004.pdf]

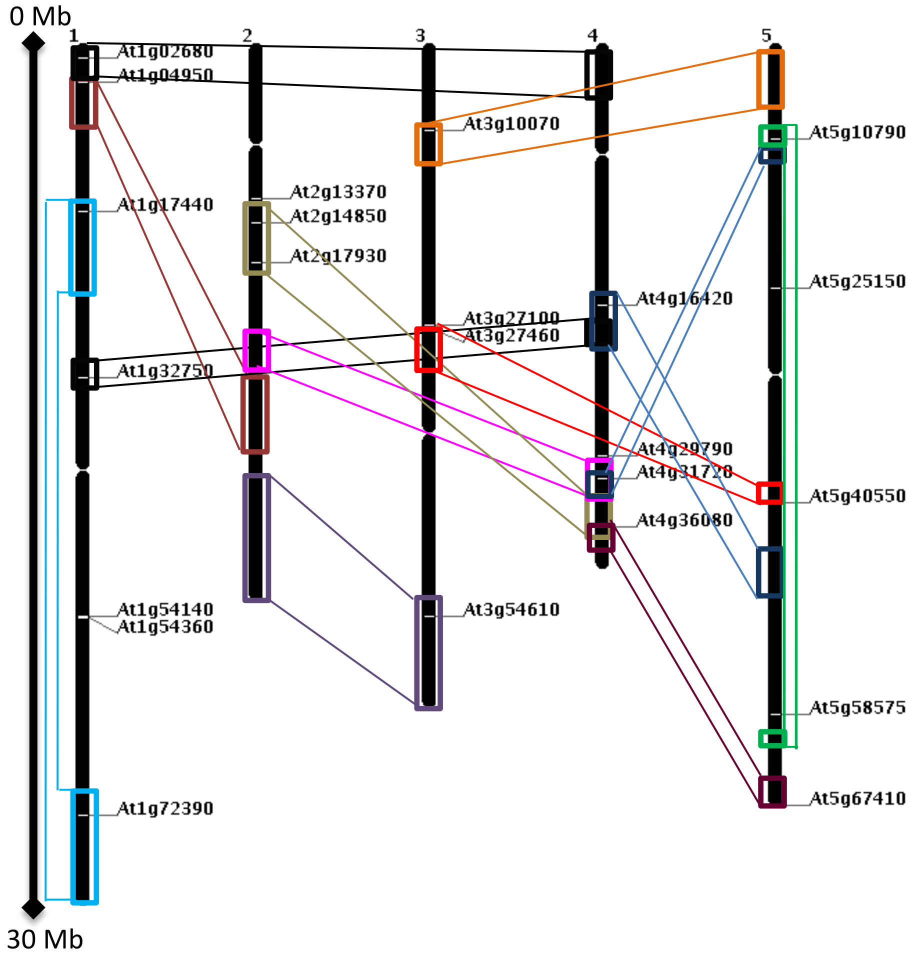

S4 Fig. Chromosomal distribution of the SAGA subunit genes in the *Arabidopsis* genome. SAGA encoding genes are plotted on the five *Arabidopsis* chromosomes according to their sequence spots. The chromosome number is shown at the top of each chromosome and the centromeric regions by constriction on chromosome line bar. Each identical duplicated chromosomal segment is marked by same line colour. The scale is in mega bases (Mb).
