# Supplementary material for: Spt-Ada-Gcn5-Acetyltransferase (SAGA) Complex in Plants: Genome Wide Identification, Evolutionary Conservation and Functional Determination
Source: PLoS One. 2015 Aug 11;10(8):e0134709. doi: 10.1371/journal.pone.0134709 (PMC4532415; doi:10.1371/journal.pone.0134709)
Supplement: S5 Fig — The co-expressed gene networks are drawn based on their rank of correlation from ATTED-II database. Orange line displays conserved co-expressed which is inferred from the comparison with mammalian co-expression data provided from COXPRESdb; Red dotted line display protein-protein interaction information that is provided from TAIR and IntAct. The octagon shape indicates transcription factor genes. White circles shape indicates SAGA complex genes which were used to give input for generating gene network. Gray circle shape indicates other genes in co-expressed gene networks. (PDF) [file pone.0134709.s005.pdf]

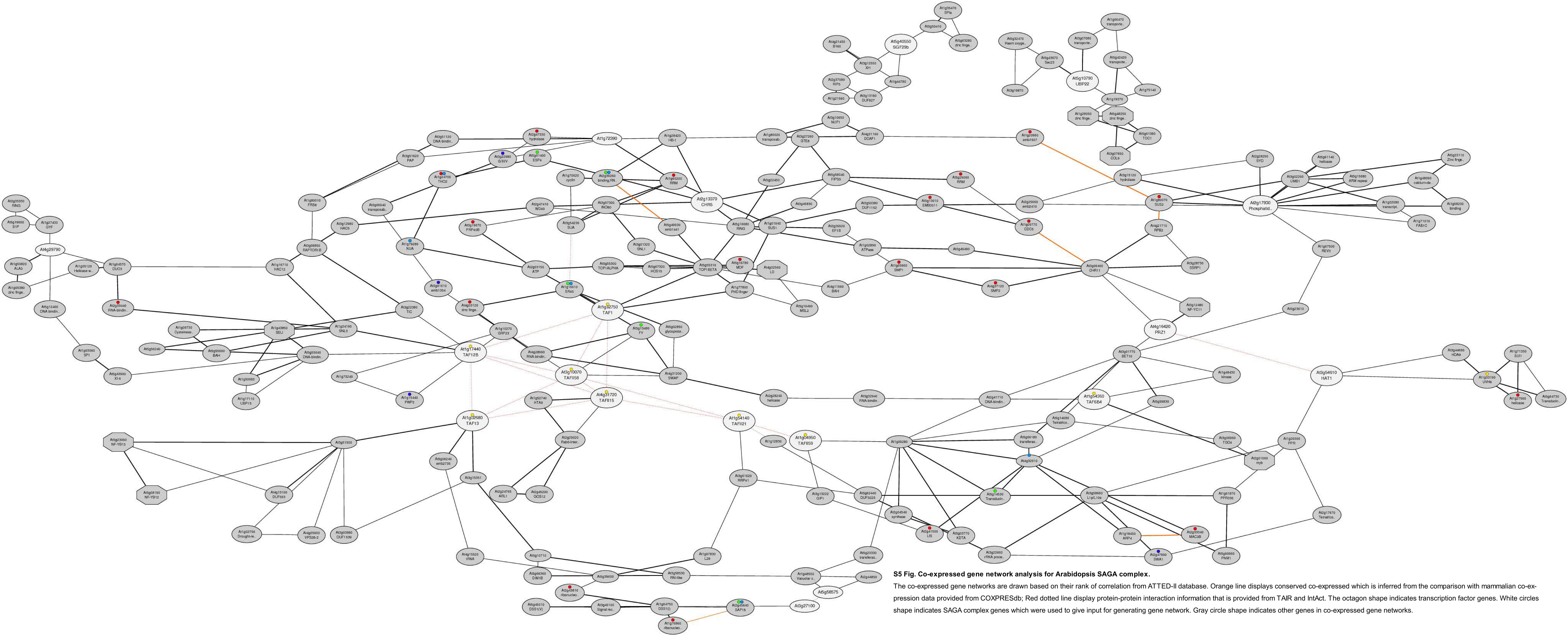

S5 Fig. Co-expressed gene network analysis for Arabidopsis SAGA complex.

The co-expressed gene networks are drawn based on their rank of correlation from ATTED-II database. Orange line displays conserved co-expressed which is inferred from the comparison with mammalian co-expression data provided from COXPRESdb; Red dotted line display protein-protein interaction information that is provided from TAIR and IntAct. The octagon shape indicates transcription factor genes. White circles shape indicates SAGA complex genes which were used to give input for generating gene network. Gray circle shape indicates other genes in co-expressed gene networks.
