# Supplementary material for: Spt-Ada-Gcn5-Acetyltransferase (SAGA) Complex in Plants: Genome Wide Identification, Evolutionary Conservation and Functional Determination
Source: PLoS One. 2015 Aug 11;10(8):e0134709. doi: 10.1371/journal.pone.0134709 (PMC4532415; doi:10.1371/journal.pone.0134709)
Supplement: S6 Fig — A biological process is analyzed by TAIR database using 181 co-expressed genes obtained from ATTED-II database. (PDF) [file pone.0134709.s006.pdf]

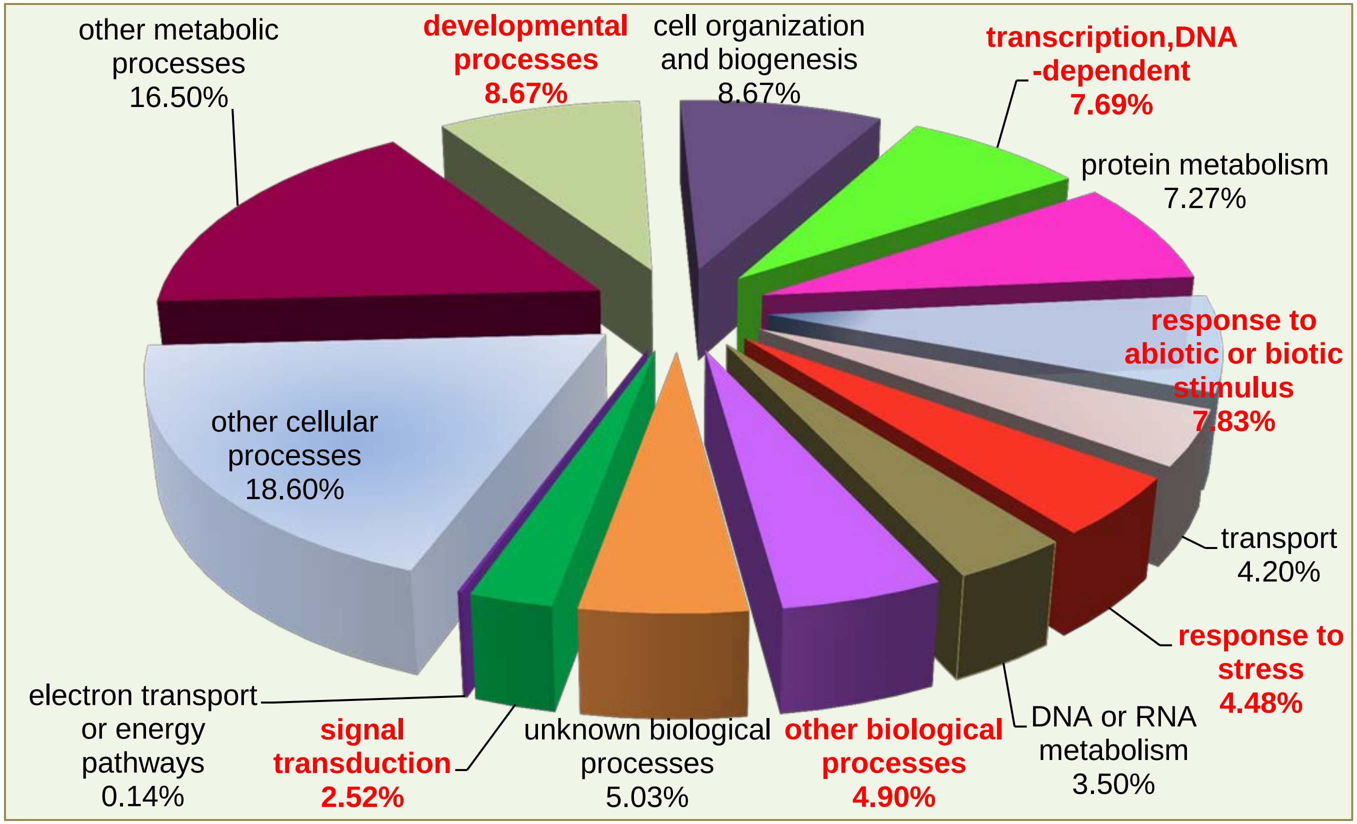

S6 Fig. Analysis of the biological process of co-expressed gene network.

A biological process is analyzed by TAIR database using 181 co-expressed genes obtained from ATTED-II database.
