# Supplementary material for: Spt-Ada-Gcn5-Acetyltransferase (SAGA) Complex in Plants: Genome Wide Identification, Evolutionary Conservation and Functional Determination
Source: PLoS One. 2015 Aug 11;10(8):e0134709. doi: 10.1371/journal.pone.0134709 (PMC4532415; doi:10.1371/journal.pone.0134709)
Supplement: S7 Fig — Characterization of Arabidopsis chr5‾, gcn5 ‾, sgf11‾, sgf29b‾, taf12b‾, taf13‾ and tra1a‾ T-DNA insertion homozygous mutants were done by qRT-PCR. RNA was isolated from homozygous T-DNA insertion mutants and Col-0 leaves or seedlings. (PDF) [file pone.0134709.s007.pdf]

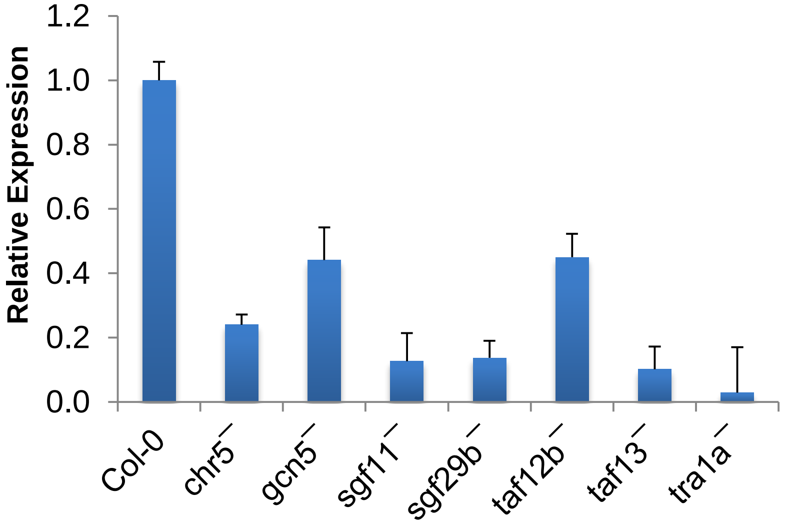

S7 Fig. Characterization of *Arabidopsis* mutant lines

Characterization of *Arabidopsis chr5*$^{-}$, *gcn5* $^{-}$, *sgf11*$^{-}$, *sgf29b*$^{-}$, *taf12b*$^{-}$, *taf13*$^{-}$ and *tra1a*$^{-}$ T-DNA insertion homozygous mutants were done by qRT-PCR. RNA was isolated from homozygous T-DNA insertion mutants and Col-0 leaves or seedlings.
