# Supplementary material for: Spt-Ada-Gcn5-Acetyltransferase (SAGA) Complex in Plants: Genome Wide Identification, Evolutionary Conservation and Functional Determination
Source: PLoS One. 2015 Aug 11;10(8):e0134709. doi: 10.1371/journal.pone.0134709 (PMC4532415; doi:10.1371/journal.pone.0134709)
Supplement: S1 Table — (PDF) [file pone.0134709.s008.pdf]

S1 Table: Query sequences from *S. cerevisiae* and human used to search *Arabidopsis* and *O. sativa* genome for SAGA gene families.

**Yeast**		**Human**		***Arabidopsis***		***O. sativa***	
**Gcn5 (Ada4)**	NP_011768.1	GCN5/PCAF	NP_003875.3	At-Gcn5	NP_567002	Os-Gcn5	NP_001064604
**Ada1**	NP_015069.1	ADA1/STAF42	NP_444281.1	At-Ada1a	NP_179091	Os-Ada1a	NP_001067130
**Ada2**	NP_010736.1	ADA2b	NP_001479.3	At-Ada1b	NP_201542	Os-Ada1b	NP_001051362
**Ada3**	NP_010461.1	ADA3	NP_006345.1	At-Ada2b	NP_974561	Os-Ada2b	NP_001051287
**Spt3**	NP_010680.1	SPT3	NP_003590.1	At-Ada3	NP_194708	Os-Ada3	NP_001055276
**Spt7**	NP_009637.1	Spt7	NP_055675.1	At-Spt3	NP_171768	Os-Spt3	BAD53126
**Spt8**	NP_013156.1	SPT20/FAM48A/p38IP	NP_060039.1	At-Spt20	NP_177383	Os-Spt20	NP_001041855
**Spt20 (Ada5)**	NP_014493.1	TAF5	NP_008882.2	At-Taf5	NP_197897	Os-Taf5	NP_001058217
**Taf5**	NP_009757.1	TAF6	NP_005632.1	At-Taf6	NP_171987	Os-Taf6	NP_001043168
**Taf6**	NP_011403.1	TAF9/TAF9b	NP_001015892.1	At-Taf6b	NP_175838		
**Taf9**	NP_013963.1	TAF10	NP_006275.1	At-Taf9	NP_175816	Os-Taf9	NP_001050338
**Taf10**	NP_010451.1	TAF12	NP_001128690.1			Os-Taf9b	NP_001060252
**Taf12**	NP_010429.1	TRRAP	NP_003487.1	At-Taf10	NP_194900	Os-Taf10	NP_001063238
**Tra1**	NP_011967.1	SGF29/STAF36	NP_612423.1	At-Taf12	NP_566367	Os-Taf12	NP_001044860
**Sgf29**	NP_009917.1	USP22	NP_056091.1	At-Taf12b	NP_849680	Os-Taf12b	NP_001044793
**Ubp8**	NP_013950.1	ATXN7L3	NP_001092303.	At-Tra1a	NP_179383	Os-Tra1	NP_001060452
**Sgf11**	NP_015278.1	ENY2	NP_064574.1	At-Tra1b	NP_680770		
**Sus1**	NP_878049.2	ATXN7	NP_000324.1	At-Sgf29a	NP_189382	Os-Sgf29	NP_001066601
**Sgf73**	NP_011449.1	Chd1	NP_001262.3	At-Sgf29b	NP_198871		
**Chd1**	NP_011091.1			At-Ubp22	NP_568239	Os-Ubp22	NP_001054070
				At-Sgf11	NP_200665	Os-Sgf11	NP_001055278
				At-Sus1	NP_189346	Os-Sus1	NP_001045218
				At-Chd1	NP_178970	Os-Chd1	EEE67748
