# Supplementary material for: Spt-Ada-Gcn5-Acetyltransferase (SAGA) Complex in Plants: Genome Wide Identification, Evolutionary Conservation and Functional Determination
Source: PLoS One. 2015 Aug 11;10(8):e0134709. doi: 10.1371/journal.pone.0134709 (PMC4532415; doi:10.1371/journal.pone.0134709)
Supplement: S2 Table — (PDF) [file pone.0134709.s009.pdf]

S2 Table: Protein similarities of SAGA encoding gene in *Arabidopsis*, *O. sativa*, human and *S. cerevisiae*.

SAGA Subunits	*Arabidopsis thaliana*			*Oryza sativa*		Human
	Human	Yeast	*O. sativa*	Human	Yeast	Yeast
ADA1a	31.8	26.2	51./56.3	35.0	30.5	31.2
ADA1b	32.8	27.3	48.1/56.3	35.1	28.6	-
ADA2b	44.5	49.1	68.3	41.3	44.1	52.7
ADA3	16.3	26.3	51.3	15.3	24.4	28.3
CHD1	46.6	44.5	72.2	47.1	45.0	45.1
GCN5(ADA4)	37.7	50.8	72.4	35.6	53.9	34.0
SGF11	23.8	22.5	60.7	23.2	30.5	15.3
SGF29a	45.2	42.9	75.0	43.0	39.7	34.0
SGF29b	47.0	40.2	77.1	-	-	-
SPT20(ADA5)	23.1	20.8	25.9	35.3	25.9	32.5
SPT3	23.5	17.2	58.5	24.3	23.1	30.0
SUS1	65.5	43.6	85.5	68.1	44.2	55.9
TAF10	36.6	40.0	72.1	41.5	36.4	39.9
TAF12	16.4	39.1	31.2/50.5	37.5	34.5	19.6
TAF12b	21.0	39.5	36.2/42.9	19.9	37.8	-
TAF5	48.7	49.2	82.1	47.8	47.5	49.9
TAF6	46.3	47.9	77.7	47.0	53.6	39.9
TAF6b	40.7	43.3	67.3	-	-	-
TAF9	39.7	41.4	57.9	42.2	35.3	29.4
TAF9b	-	-	26.6	32.3	18.1	29.4
TRA1a	49.9	48.8	19.3	10.5	11.3	50.7
TRA1b	50.3	48.4	19.2	-	-	-
UBP8	51.6	42.7	57.1	48.5	39.0	45.0
