# Supplementary material for: Spt-Ada-Gcn5-Acetyltransferase (SAGA) Complex in Plants: Genome Wide Identification, Evolutionary Conservation and Functional Determination
Source: PLoS One. 2015 Aug 11;10(8):e0134709. doi: 10.1371/journal.pone.0134709 (PMC4532415; doi:10.1371/journal.pone.0134709)
Supplement: S3 Table — (PDF) [file pone.0134709.s010.pdf]

S3 Table: List of primer used in qRT-PCR

S.No.	Locus ID	Primer Sequence
1	AT4G05320 F	5’CACACTCCACTTGGTCTTGCGT3’
2	AT4G05320 R	5’TGGTCTTTCCGGTGAGAGTCTTCA3’
3	AT2G13370 F	5’GGGCATTGACACAGCAAAGTT3’
4	AT2G13370 R	5’TCGTTGGTCGCTCAGTCTGA3’
5	AT5G40550 F	5’GGGACAACAGCACTCTACAAAGC3’
6	AT5G40550 R	5’CAATGCTCCATCTTCCTCATCAT3’
7	AT1G02680 F	5’CCCATTATCTAGCATCCCGTATATATC3’
8	AT1G02680 R	5’CAAAAATCGGGTGGTTATTCTAAATT3’
9	AT2G17930 F	5’TGATGAGCGGAAGCCATATG3’
10	AT2G17930 R	5’GACCTCCCACCAATTTGTAGCA3’
11	AT5G10790 F	5’TGTATCACCCACAAGGCATTCT3’
12	AT5G10790 R	5’AAACGGGTCATATGTTGTTGATGT3’
13	AT1G72390 F	5’GTCTGTTCCTGGCATCGGTAA3’
14	AT1G72390 R	5’TTAATGCACCAGATCGGAGTTG3’
15	AT3G54610 F	5'GTAGAACTTACAACTCCCCCGATACC3'
16	AT3G54610 R	5’GATTGGAGACCTGCTTGTACTTTGCT3’
17	AT5G67410 F	5’CGGGCCAAAACAGAGAGTTA3’
18	AT5G67410 R	5’ATTCTCCCTCTTCACCGTCA3’
19	AT5G58575 F	5’GCAACAACCTCGAAACCAACA3’
20	AT5G58575 R	5’CGCCCACAGTTCATGCAAT3’
21	AT1G04950 F	5’CCCACCTTTACCATCTCCGTC3’
22	AT1G04950 R	5’TGTGGGGATCAGTACTGATTATC3’
23	AT1G67090 F	5`ATGGCTTCCTCTATGCTCTCTTC3`
24	AT1G67090 R	5`TTCGGAATCGGTAAGGTCAGGA3`
25	AT5G12030 F	5’TGCTTGTGGTGAGTGGCAAA3’
26	AT5G12030 R	5’TTCCCCATCCTCCTCTCCAT3’
27	AT1G71000 F	5’GGTGGCATTAGGCAAACGTATT3’
28	AT1G71000 R	5’GCTTGTGATATGCTCGTCGTATCT3’
29	AT2G40140 F	5’CTCAGATGAACCACTATCCGTCTTC3’
30	AT2G40140 R	5’CAAACGCTGAGGATCTTGCAT3’
31	AT4G02770 F	5`ATGGCAACTCAAGCCGCCGG3`
32	AT4G02770 R	5`ATCGGAGACGGTGTGTTTGGGT3`
33	AT1G56600 F	5’ GCGGTTTTGCCGGATGT3’
34	AT1G56600 R	5’GGTTTGATTCTCCGGTGGATAA3’
35	AT1G17440 F	5’CTCCGGCGATGACTGTAACT3’
36	AT1G17440R	5’ CCGATTGGTGGGTTAGAAGA3’
