# Supplementary material for: Spt-Ada-Gcn5-Acetyltransferase (SAGA) Complex in Plants: Genome Wide Identification, Evolutionary Conservation and Functional Determination
Source: PLoS One. 2015 Aug 11;10(8):e0134709. doi: 10.1371/journal.pone.0134709 (PMC4532415; doi:10.1371/journal.pone.0134709)
Supplement: S4 Table — (PDF) [file pone.0134709.s011.pdf]

S4 Table: Gene Ontology list of *Arabidopsis* and *O. sativa* for SAGA complex gene.

.*Arabidopsis* SAGA Gene Ontology

Cellular component	Molecular function	Biological process
Chloroplast	ATP binding	Chromatin assembly or disassembly
Chloroplast envelope	Chromatin binding	Cold acclimation
Cytosol	DNA binding	Cytokinin mediated signaling pathway
Histone acetyltransferase complex	H3 histone acetyltransferase activity	Flower development
Nucleus	Helicase activity	Histone acetylation
Plasmodesma	Histone acetyltransferase activity	Histone h3 acetylation
Transcription factor complex	Inositol or phosphatidylinositol kinase activity	Histone h4 acetylation
Transcription factor tfiid complex	Nucleic acid binding	Jasmonic acid mediated signaling pathway
	Nucleotide binding	Phosphorylation
	Phosphotransferase activity, alcohol group as acceptor	Pollen tube growth
	Protein binding	Positive regulation of transcription, dna-dependent
	Sequence-specific dna binding transcription factor activity	Regulation of cell proliferation
	Transcription coactivator activity	Regulation of ethylene mediated signaling pathway
	Ubiquitin thiolesterase activity	Regulation of sequence-specific dna binding transcription factor activity
	Ubiquitin-specific protease activity	Regulation of transcription, dna-dependent
	Zinc ion binding	Response to auxin stimulus
		Response to cytokinin stimulus
		Response to light stimulus
		Response to salt stress
		Root morphogenesis
		Transcription from rna polymerase ii promoter
		Transcription initiation, dna-dependent
		Ubiquitin-dependent protein catabolic process

O. sativa SAGA Gene Ontology

Cellular component	Molecular function	Biological process
Golgi apparatus	DNA binding	Transport
Plasma membrane	Transcription factor activity	Signal transduction
Membrane	Nucleotide binding	Cellular component organization and biogenesis
Nucleus	Signal transducer activity	Transcription
Nucleoplasm	Transporter activity	Catabolic process
Intracellular	Transcription regulator activity	Cellular process
	Kinase activity	Protein metabolic process
	Hydrolase activity	
