# Supplementary material for: Spt-Ada-Gcn5-Acetyltransferase (SAGA) Complex in Plants: Genome Wide Identification, Evolutionary Conservation and Functional Determination
Source: PLoS One. 2015 Aug 11;10(8):e0134709. doi: 10.1371/journal.pone.0134709 (PMC4532415; doi:10.1371/journal.pone.0134709)
Supplement: S5 Table — (PDF) [file pone.0134709.s012.pdf]

S5 Table: List of *Arabidopsis* SAGA complex subunits known and predicted protein interactions from STRING database.

#Node1	Node2	Neighborhood	Fusion	Co-occurrence	Homology	Co-expression	Experimental	Knowledge	Text mining	Combined Score
ADA2B	CHR5	0	0	0	0	0	0.641	0	0	0.641
ADA2B	GCN5	0	0	0	0	0	0.998	0	0.908	0.999
ADA2B	HAF01	0	0	0	0	0	0.713	0	0	0.713
ADA2B	SGF29a	0	0	0	0	0	0.714	0	0.292	0.784
ADA2B	TAF12	0	0	0	0	0	0.714	0	0.429	0.826
ADA2B	TAF12B	0	0	0	0	0	0.714	0	0.429	0.826
ADA2B	TAF6	0	0	0	0	0	0.714	0	0.429	0.826
ADA2B	TAF6B4	0	0	0	0	0	0.714	0	0.429	0.826
ADA2B	TAF9	0	0	0	0	0	0.769	0.526	0.758	0.969
ADA2B	TRA1b	0	0	0	0	0	0.714	0	0.429	0.826
CHR5	TAF9	0	0	0	0	0	0.604	0	0	0.604
ENY2	TAF9	0	0	0	0	0	0	0	0.818	0.818
ENY2	TRA1b	0	0	0	0	0	0.622	0	0	0.622
GCN5	CHR5	0	0	0	0	0	0.593	0	0.234	0.667
GCN5	HAF01	0	0	0	0	0	0.638	0	0.207	0.693
GCN5	SGF29a	0	0	0	0	0	0.648	0	0.24	0.714
GCN5	TAF12	0	0	0	0	0	0.638	0.525	0.344	0.871
GCN5	TAF12B	0	0	0	0	0	0.638	0.525	0.344	0.871
GCN5	TAF6	0	0	0	0	0	0.647	0	0.336	0.749
GCN5	TAF6B4	0	0	0	0	0	0.647	0	0.336	0.749
GCN5	TAF9	0	0	0	0	0	0.72	0.526	0.368	0.904
GCN5	TRA1b	0	0	0	0	0	0.648	0.525	0.336	0.873
HAF01	TAF12B	0	0	0	0	0	0.999	0.9	0.344	0.999
HAF01	TAF13	0	0	0	0	0	0.769	0.9	0.5	0.986
HAF01	TAF6	0	0	0	0	0	0	0.9	0.499	0.946
SGF11	ADA2B	0	0	0	0	0	0.768	0	0.5	0.876
SGF11	ENY2	0	0	0	0	0	0.797	0	0.372	0.864
SGF11	GCN5	0	0	0	0	0	0.72	0	0.368	0.811
SGF11	SGF29a	0	0	0	0	0	0.72	0	0.336	0.801
SGF11	SGF29b	0	0	0	0	0	0.72	0	0.336	0.801
SGF11	TAF10	0	0	0	0	0	0.775	0	0.549	0.891
SGF11	TAF12	0	0	0	0	0	0.72	0	0.544	0.863
SGF11	TAF12B	0	0	0	0	0	0.72	0	0.544	0.863
SGF11	TAF5	0	0	0	0	0	0.768	0	0.544	0.887
SGF11	TAF6	0	0	0	0	0	0.768	0	0.544	0.887
SGF11	TAF6B4	0	0	0	0	0	0.768	0	0.544	0.887
SGF11	TAF9	0	0	0	0	0	0.775	0	0.549	0.891
SGF11	TRA1a	0	0	0	0	0	0.72	0	0.544	0.863
SGF11	TRA1b	0	0	0	0	0	0.72	0	0.544	0.863
SGF11	UBP22	0	0	0	0	0	0.775	0	0.858	0.966
SGF29a	TAF12	0	0	0	0	0	0.712	0	0.236	0.765
SGF29a	TAF12B	0	0	0	0	0	0.712	0	0.236	0.765
SGF29a	TAF6	0	0	0	0	0	0.713	0	0.096	0.723

SGF29a	TAF6B4	0	0	0	0	0	0.713	0	0.096	0.723
SGF29a	TAF9	0	0	0	0	0	0.72	0	0	0.72
SGF29a	TRA1b	0	0	0	0	0	0.712	0	0.121	0.73
SGF29b	ADA2B	0	0	0	0	0	0.714	0	0.292	0.784
SGF29b	GCN5	0	0	0	0	0	0.648	0	0.24	0.714
SGF29b	TAF10	0	0	0	0	0	0.72	0	0.119	0.736
SGF29b	TAF12	0	0	0	0	0	0.712	0	0.236	0.765
SGF29b	TAF12B	0	0	0	0	0	0.712	0	0.236	0.765
SGF29b	TAF5	0	0	0	0	0	0.712	0	0.122	0.73
SGF29b	TAF6	0	0	0	0	0	0.713	0	0.096	0.723
SGF29b	TAF6B4	0	0	0	0	0	0.713	0	0.096	0.723
SGF29b	TAF9	0	0	0	0	0	0.72	0	0	0.72
SGF29b	TRA1a	0	0	0	0	0	0.712	0	0.121	0.73
SGF29b	TRA1b	0	0	0	0	0	0.712	0	0.121	0.73
SGF29b	UBP22	0	0	0	0	0	0.72	0	0.28	0.785
TAF10	ADA2B	0	0	0	0	0	0.769	0	0.5	0.877
TAF10	CHR5	0	0	0	0	0	0.604	0	0	0.604
TAF10	ENY2	0	0	0	0	0	0.715	0	0.122	0.733
TAF10	GCN5	0	0	0	0	0	0.72	0.526	0.759	0.963
TAF10	HAF01	0	0	0	0	0	0.999	0.9	0.424	0.999
TAF10	SGF29a	0	0	0	0	0	0.72	0	0.119	0.736
TAF10	TAF12	0	0	0	0	0	0.999	0.949	0.587	0.999
TAF10	TAF12B	0	0	0	0	0	0.999	0.949	0.567	0.999
TAF10	TAF13	0	0	0	0	0	0.999	0.955	0.586	0.999
TAF10	TAF6	0	0	0	0	0	0	0.949	0.57	0.976
TAF10	TAF6B4	0	0	0	0	0	0.999	0.949	0	0.999
TAF10	TAF9	0	0	0	0	0.071	0.999	0.954	0.79	0.999
TAF10	TRA1b	0	0	0	0	0	0.768	0.527	0.717	0.964
TAF12	HAF01	0	0	0	0	0	0.999	0.9	0.344	0.999
TAF12	TAF12B	0	0	0	0.81	0	0.999	0.9	0.749	0.999
TAF12	TAF13	0	0	0	0	0	0.999	0.949	0.544	0.999
TAF12	TAF6	0	0	0	0	0	0	0.949	0	0.949
TAF12	TAF6B4	0	0	0	0	0	0.999	0.949	0	0.999
TAF12	TAF9	0	0	0	0	0	0.999	0.949	0.544	0.999
TAF12	TRA1b	0	0	0	0	0	0.713	0.525	0.432	0.912
TAF12B	TAF13	0	0	0	0	0	0.999	0.949	0.565	0.999
TAF12B	TAF6	0	0	0	0	0	0	0.949	0.287	0.961
TAF5	ADA2B	0	0	0	0	0	0.713	0	0.429	0.825
TAF5	GCN5	0	0	0	0	0	0.648	0	0.645	0.866
TAF5	HAF01	0	0	0	0	0	0.999	0.9	0.499	0.999
TAF5	SGF29a	0	0	0	0	0	0.712	0	0.122	0.73
TAF5	TAF10	0	0	0	0	0	0.999	0.949	0.28	0.999
TAF5	TAF12	0	0	0	0	0	0.999	0.949	0.595	0.999
TAF5	TAF12B	0	0	0	0	0	0.999	0.949	0	0.999
TAF5	TAF13	0	0	0	0	0	0.768	0.949	0.596	0.994
TAF5	TAF6	0	0	0	0	0	0	0.949	0.564	0.976
TAF5	TAF6B4	0	0	0	0	0	0.999	0.949	0.564	0.999
TAF5	TAF9	0	0	0	0	0.074	0.999	0.949	0.544	0.999
TAF5	TRA1a	0	0	0	0	0	0.713	0	0.432	0.826
TAF5	TRA1b	0	0	0	0	0	0.713	0	0.432	0.826
TAF5	UBP22	0	0	0	0	0	0.663	0	0.5	0.82
TAF6	TAF13	0	0	0	0	0	0	0.949	0.254	0.959

TAF6B4	HAF01	0	0	0	0	0	0.999	0.9	0.499	0.999
TAF6B4	TAF12B	0	0	0	0	0	0.999	0.949	0	0.999
TAF6B4	TAF13	0	0	0	0	0	0.999	0.949	0.135	0.999
TAF6B4	TAF6	0	0	0	0.916	0.108	0.999	0.9	0	0.999
TAF6B4	TAF9	0	0	0	0	0	0.999	0.949	0.547	0.999
TAF9	HAF01	0	0	0	0	0	0.772	0.9	0.342	0.982
TAF9	TAF12B	0	0	0	0	0	0	0.949	0.544	0.975
TAF9	TAF13	0	0	0	0	0.07	0.8	0.954	0.115	0.99
TAF9	TAF6	0	0	0	0	0	0.999	0.949	0.981	0.999
TRA1a	ADA2B	0	0	0	0	0	0.714	0	0.429	0.826
TRA1a	ENY2	0	0	0	0	0	0.622	0	0	0.622
TRA1a	GCN5	0	0	0	0	0	0.648	0.525	0.336	0.873
TRA1a	SGF29a	0	0	0	0	0	0.712	0	0.121	0.73
TRA1a	TAF10	0	0	0	0	0	0.768	0.527	0	0.883
TRA1a	TAF12	0	0	0	0	0	0.713	0.525	0.432	0.912
TRA1a	TAF12B	0	0	0	0	0	0.713	0.525	0.432	0.912
TRA1a	TAF6	0	0	0	0	0	0.713	0	0	0.713
TRA1a	TAF6B4	0	0	0	0	0	0.713	0	0	0.713
TRA1a	TAF9	0	0	0	0	0	0.768	0.526	0	0.883
TRA1b	CHR5	0	0	0	0	0.294	0.54	0	0.092	0.664
TRA1b	TAF12B	0	0	0	0	0	0.713	0.525	0.432	0.912
TRA1b	TAF6	0	0	0	0	0	0.713	0	0.921	0.975
TRA1b	TAF6B4	0	0	0	0	0	0.713	0	0	0.713
TRA1b	TAF9	0	0	0	0	0	0.768	0.526	0.993	0.999
UBP22	ADA2B	0	0	0	0	0	0.663	0	0.368	0.773
UBP22	ENY2	0	0	0	0	0	0.544	0	0.372	0.694
UBP22	GCN5	0	0	0	0	0	0.719	0	0.34	0.802
UBP22	SGF29a	0	0	0	0	0	0.72	0	0.28	0.785
UBP22	TAF10	0	0	0	0	0	0.72	0	0.544	0.863
UBP22	TAF12	0	0	0	0	0	0.662	0	0.5	0.819
UBP22	TAF12B	0	0	0	0	0	0.662	0	0.5	0.819
UBP22	TAF6	0	0	0	0	0	0.663	0	0.5	0.82
UBP22	TAF6B4	0	0	0	0	0	0.663	0	0.5	0.82
UBP22	TAF9	0	0	0	0	0	0.72	0	0.544	0.863
UBP22	TRA1a	0	0	0	0	0	0.72	0	0.5	0.85
UBP22	TRA1b	0	0	0	0	0	0.72	0	0.5	0.85
