# Supplementary material for: Spt-Ada-Gcn5-Acetyltransferase (SAGA) Complex in Plants: Genome Wide Identification, Evolutionary Conservation and Functional Determination
Source: PLoS One. 2015 Aug 11;10(8):e0134709. doi: 10.1371/journal.pone.0134709 (PMC4532415; doi:10.1371/journal.pone.0134709)
Supplement: S6 Table — (PDF) [file pone.0134709.s013.pdf]

S6 Table: List of *S. cerevisiae* SAGA complex subunits known and predicted protein interactions from STRING database.

#Node1	Node2	Node1 External ID	Node2 External ID	Neighborhood	Fusion	Co-occurrence	Homology	Co-expression	Experimental	Knowledge	Text mining	Combined Score
ADA2	TAF10	YDR448W	YDR167W	0	0	0	0	0	0.999	0.9	0.728	0.999
ADA2	SUS1	YDR448W	YBR111W-A	0	0	0	0	0	0.999	0.9	0	0.999
ADA2	TAF5	YDR448W	YBR198C	0	0	0	0	0	0.999	0.9	0.728	0.999
ADA2	TAF12	YDR448W	YDR145W	0	0	0	0	0	0.999	0.9	0.728	0.999
ADA2	SPT7	YDR448W	YBR081C	0	0	0	0	0	0.999	0.9	0.774	0.999
ADA2	SGF29	YDR448W	YCL010C	0	0	0	0	0	0.999	0.9	0.271	0.999
ADA2	NGG1	YDR448W	YDR176W	0	0	0	0	0	0.999	0.9	0.896	0.999
ADA2	SPT3	YDR448W	YDR392W	0	0	0	0	0	0.999	0.9	0.779	0.999
CHD1	SGF29	YER164W	YCL010C	0	0	0	0	0	0.814	0.9	0.094	0.98
CHD1	SPT7	YER164W	YBR081C	0	0	0	0	0.115	0.814	0.9	0.488	0.989
CHD1	TAF12	YER164W	YDR145W	0	0	0	0	0.071	0.814	0.9	0.109	0.981
CHD1	NGG1	YER164W	YDR176W	0	0	0	0	0	0.814	0.9	0.063	0.98
CHD1	ADA2	YER164W	YDR448W	0	0	0	0	0	0.997	0.9	0	0.999
CHD1	TAF5	YER164W	YBR198C	0	0	0	0	0	0.814	0.9	0.285	0.984
CHD1	SUS1	YER164W	YBR111W-A	0	0	0	0	0	0	0.9	0.283	0.923
CHD1	SPT3	YER164W	YDR392W	0	0	0	0	0	0.87	0.9	0.119	0.986
CHD1	TAF10	YER164W	YDR167W	0	0	0	0	0	0.814	0.9	0.316	0.985
GCN5	SPT7	YGR252W	YBR081C	0	0	0	0.586	0	0.999	0.9	0.874	0.999
GCN5	SPT3	YGR252W	YDR392W	0	0	0	0	0	0.999	0.948	0.951	0.999
GCN5	CHD1	YGR252W	YER164W	0	0	0	0	0	0.982	0.9	0.62	0.999
GCN5	SUS1	YGR252W	YBR111W-A	0	0	0	0	0	0.962	0.9	0.515	0.997
GCN5	SGF29	YGR252W	YCL010C	0	0	0	0	0	0.999	0.9	0.575	0.999
GCN5	TAF10	YGR252W	YDR167W	0	0	0	0	0	0.999	0.948	0.862	0.999
GCN5	TAF6	YGR252W	YGL112C	0	0	0	0	0	0.999	0.9	0.726	0.999
GCN5	SGF73	YGR252W	YGL066W	0	0	0	0	0	0.999	0.9	0.705	0.999
GCN5	TAF5	YGR252W	YBR198C	0	0	0	0	0	0.999	0.9	0.908	0.999
GCN5	TAF12	YGR252W	YDR145W	0	0	0	0	0	0.999	0.948	0.726	0.999
GCN5	ADA2	YGR252W	YDR448W	0	0	0	0	0	0.999	0.9	0.991	0.999
GCN5	NGG1	YGR252W	YDR176W	0	0	0	0	0	0.999	0.9	0.851	0.999
HFI1	SPT8	YPL254W	YLR055C	0	0	0	0	0	0.999	0.9	0.725	0.999
HFI1	CHD1	YPL254W	YER164W	0	0	0	0	0	0.814	0.9	0	0.98
HFI1	TAF12	YPL254W	YDR145W	0	0	0	0	0	0.999	0.9	0.726	0.999
HFI1	SGF11	YPL254W	YPL047W	0	0	0	0	0	0.997	0.9	0.726	0.999
HFI1	SPT20	YPL254W	YOL148C	0	0	0	0	0	0.999	0.9	0.729	0.999
HFI1	SPT7	YPL254W	YBR081C	0	0	0	0	0	0.999	0.9	0.729	0.999
HFI1	GCN5	YPL254W	YGR252W	0	0	0	0	0.146	0.999	0.9	0.781	0.999
HFI1	SUS1	YPL254W	YBR111W-A	0	0	0	0	0	0.843	0.9	0	0.983
HFI1	SPT3	YPL254W	YDR392W	0	0	0	0	0	0.999	0.9	0.773	0.999
HFI1	SGF29	YPL254W	YCL010C	0	0	0	0	0	0.999	0.9	0	0.999
HFI1	TRA1	YPL254W	YHR099W	0	0	0	0	0	0.999	0.9	0.726	0.999
HFI1	SGF73	YPL254W	YGL066W	0	0	0	0	0	0.999	0.9	0	0.999

HFI1	NGG1	YPL254W	YDR176W	0	0	0	0	0	0.999	0.9	0.774	0.999
HFI1	TAF6	YPL254W	YGL112C	0	0	0	0	0	0.999	0.9	0.726	0.999
HFI1	ADA2	YPL254W	YDR448W	0	0	0	0	0	0.999	0.9	0.855	0.999
HFI1	TAF9	YPL254W	YMR236W	0	0	0	0	0	0.999	0.9	0.726	0.999
HFI1	TAF5	YPL254W	YBR198C	0	0	0	0	0	0.999	0.9	0.726	0.999
HFI1	TAF10	YPL254W	YDR167W	0	0	0	0	0	0.999	0.9	0.726	0.999
HFI1	UBP8	YPL254W	YMR223W	0	0	0	0	0	0.999	0.9	0.726	0.999
NGG1	SPT7	YDR176W	YBR081C	0	0	0	0.458	0	0.999	0.9	0.854	0.999
NGG1	TAF10	YDR176W	YDR167W	0	0	0	0	0	0.999	0.9	0.772	0.999
NGG1	TAF12	YDR176W	YDR145W	0	0	0	0	0	0.999	0.9	0.772	0.999
NGG1	TAF5	YDR176W	YBR198C	0	0	0	0	0	0.999	0.9	0.772	0.999
NGG1	SUS1	YDR176W	YBR111W-A	0	0	0	0	0	0.992	0.9	0.12	0.999
NGG1	SGF29	YDR176W	YCL010C	0	0	0	0	0	0.999	0.9	0.402	0.999
SGF11	TAF12	YPL047W	YDR145W	0	0	0	0	0	0.998	0.9	0.777	0.999
SGF11	SGF73	YPL047W	YGL066W	0	0	0	0	0	0.999	0.9	0.81	0.999
SGF11	SPT8	YPL047W	YLR055C	0	0	0	0	0	0.998	0.9	0.776	0.999
SGF11	TAF9	YPL047W	YMR236W	0	0	0	0	0	0.985	0.9	0.777	0.999
SGF11	SGF29	YPL047W	YCL010C	0	0	0	0	0	0.99	0.9	0.62	0.999
SGF11	TAF6	YPL047W	YGL112C	0	0	0	0	0	0.999	0.9	0.777	0.999
SGF11	SPT20	YPL047W	YOL148C	0	0	0	0	0	0.999	0.9	0.728	0.999
SGF11	GCN5	YPL047W	YGR252W	0	0	0	0	0	0.999	0.9	0.726	0.999
SGF11	ADA2	YPL047W	YDR448W	0	0	0	0	0	0.999	0.9	0.728	0.999
SGF11	SPT7	YPL047W	YBR081C	0	0	0	0	0	0.999	0.9	0.729	0.999
SGF11	SUS1	YPL047W	YBR111W-A	0	0	0	0	0	0.999	0.9	0.85	0.999
SGF11	TAF10	YPL047W	YDR167W	0	0	0	0	0	0.997	0.9	0.777	0.999
SGF11	TRA1	YPL047W	YHR099W	0	0	0	0	0	0.996	0.9	0.777	0.999
SGF11	TAF5	YPL047W	YBR198C	0	0	0	0	0	0.999	0.9	0.777	0.999
SGF11	UBP8	YPL047W	YMR223W	0	0	0	0	0	0.999	0.9	0.974	0.999
SGF11	SPT3	YPL047W	YDR392W	0	0	0	0	0	0.989	0.9	0.771	0.999
SGF11	NGG1	YPL047W	YDR176W	0	0	0	0	0	0.999	0.9	0.772	0.999
SGF11	CHD1	YPL047W	YER164W	0	0	0	0	0	0	0.9	0.35	0.93
SGF29	SUS1	YCL010C	YBR111W-A	0	0	0	0	0	0.827	0.9	0.4	0.988
SGF29	SPT7	YCL010C	YBR081C	0	0	0	0	0	0.999	0.9	0.62	0.999
SGF29	TAF5	YCL010C	YBR198C	0	0	0	0	0	0.999	0.9	0.428	0.999
SGF73	SPT3	YGL066W	YDR392W	0	0	0	0	0	0.999	0.9	0.233	0.999
SGF73	SPT7	YGL066W	YBR081C	0	0	0	0	0	0.999	0.9	0.74	0.999
SGF73	SUS1	YGL066W	YBR111W-A	0	0	0	0	0	0.999	0.9	0.702	0.999
SGF73	CHD1	YGL066W	YER164W	0	0	0	0	0	0	0.9	0.186	0.913
SGF73	TAF10	YGL066W	YDR167W	0	0	0	0	0	0.999	0.9	0.41	0.999
SGF73	TAF5	YGL066W	YBR198C	0	0	0	0	0	0.999	0.9	0.512	0.999
SGF73	ADA2	YGL066W	YDR448W	0	0	0	0	0	0.999	0.9	0.256	0.999
SGF73	NGG1	YGL066W	YDR176W	0	0	0	0	0	0.999	0.9	0.427	0.999
SGF73	SGF29	YGL066W	YCL010C	0	0	0	0	0	0.999	0.9	0.81	0.999
SGF73	TAF12	YGL066W	YDR145W	0	0	0	0	0	0.999	0.9	0.62	0.999
SPT20	CHD1	YOL148C	YER164W	0	0	0	0	0	0.814	0.9	0.46	0.988
SPT20	SPT3	YOL148C	YDR392W	0	0	0	0	0	0.999	0.9	0.779	0.999
SPT20	SPT7	YOL148C	YBR081C	0	0	0	0	0	0.999	0.9	0.82	0.999
SPT20	SPT8	YOL148C	YLR055C	0	0	0	0	0	0.999	0.9	0.812	0.999
SPT20	SGF29	YOL148C	YCL010C	0	0	0	0	0	0.999	0.9	0	0.999
SPT20	NGG1	YOL148C	YDR176W	0	0	0	0	0	0.999	0.9	0.78	0.999

SPT20	UBP8	YOL148C	YMR223W	0	0	0	0	0	0.999	0.9	0.728	0.999
SPT20	GCN5	YOL148C	YGR252W	0	0	0	0	0	0.999	0.9	0.808	0.999
SPT20	ADA2	YOL148C	YDR448W	0	0	0	0	0	0.999	0.9	0.772	0.999
SPT20	TAF10	YOL148C	YDR167W	0	0	0	0	0	0.999	0.9	0.728	0.999
SPT20	TRA1	YOL148C	YHR099W	0	0	0	0	0	0.999	0.9	0.728	0.999
SPT20	TAF12	YOL148C	YDR145W	0	0	0	0	0	0.999	0.9	0.728	0.999
SPT20	SGF73	YOL148C	YGL066W	0	0	0	0	0	0.999	0.9	0	0.999
SPT20	TAF6	YOL148C	YGL112C	0	0	0	0	0	0.999	0.9	0.728	0.999
SPT20	TAF9	YOL148C	YMR236W	0	0	0	0	0	0.999	0.9	0.728	0.999
SPT20	TAF5	YOL148C	YBR198C	0	0	0	0	0	0.999	0.9	0.728	0.999
SPT20	SUS1	YOL148C	YBR111W-A	0	0	0	0	0	0.963	0.9	0.23	0.996
SPT3	NGG1	YDR392W	YDR176W	0	0	0	0	0	0.999	0.9	0.78	0.999
SPT3	SGF29	YDR392W	YCL010C	0	0	0	0	0	0.998	0.9	0.269	0.999
SPT3	TAF5	YDR392W	YBR198C	0	0	0	0	0	0.999	0.9	0.771	0.999
SPT3	TAF10	YDR392W	YDR167W	0	0	0	0	0	0.999	0.948	0.771	0.999
SPT3	TAF12	YDR392W	YDR145W	0	0	0	0	0	0.999	0.948	0.771	0.999
SPT3	SPT7	YDR392W	YBR081C	0	0	0	0	0	0.999	0.948	0.78	0.999
SPT3	SUS1	YDR392W	YBR111W-A	0	0	0	0	0	0.81	0.9	0	0.979
SPT8	TAF6	YLR055C	YGL112C	0	0	0	0	0	0.999	0.9	0.776	0.999
SPT8	TAF5	YLR055C	YBR198C	0	0	0	0	0	0.999	0.9	0.808	0.999
SPT8	TAF10	YLR055C	YDR167W	0	0	0	0	0	0.999	0.9	0.776	0.999
SPT8	NGG1	YLR055C	YDR176W	0	0	0	0	0	0.999	0.9	0.771	0.999
SPT8	GCN5	YLR055C	YGR252W	0	0	0	0	0	0.999	0.9	0.81	0.999
SPT8	SPT3	YLR055C	YDR392W	0	0	0	0	0	0.999	0.9	0.73	0.999
SPT8	TRA1	YLR055C	YHR099W	0	0	0	0	0	0.999	0.9	0.776	0.999
SPT8	SGF73	YLR055C	YGL066W	0	0	0	0	0	0.999	0.9	0.651	0.999
SPT8	TAF12	YLR055C	YDR145W	0	0	0	0	0	0.999	0.9	0.776	0.999
SPT8	SUS1	YLR055C	YBR111W-A	0	0	0	0	0	0.982	0.9	0.48	0.998
SPT8	ADA2	YLR055C	YDR448W	0	0	0	0	0	0.999	0.9	0.728	0.999
SPT8	SPT7	YLR055C	YBR081C	0	0	0	0	0	0.999	0.9	0.921	0.999
SPT8	CHD1	YLR055C	YER164W	0	0	0	0	0	0.361	0.9	0.482	0.962
SPT8	SGF29	YLR055C	YCL010C	0	0	0	0	0	0.999	0.9	0.652	0.999
SUS1	SPT7	YBR111W-A	YBR081C	0	0	0	0	0	0.999	0.9	0.576	0.999
TAF10	TAF5	YDR167W	YBR198C	0	0	0	0	0	0.999	0.948	0.92	0.999
TAF10	SGF29	YDR167W	YCL010C	0	0	0	0	0	0.999	0.9	0.35	0.999
TAF10	SUS1	YDR167W	YBR111W-A	0	0	0	0	0	0.81	0.9	0.087	0.98
TAF10	SPT7	YDR167W	YBR081C	0	0	0	0	0	0.999	0.948	0.959	0.999
TAF10	TAF12	YDR167W	YDR145W	0	0	0	0	0	0.999	0.948	0.879	0.999
TAF12	SUS1	YDR145W	YBR111W-A	0	0	0	0	0	0.992	0.9	0.362	0.999
TAF12	TAF5	YDR145W	YBR198C	0	0	0	0	0	0.999	0.948	0.879	0.999
TAF12	SPT7	YDR145W	YBR081C	0	0	0	0	0	0.999	0.948	0.742	0.999
TAF12	SGF29	YDR145W	YCL010C	0	0	0	0	0	0.999	0.9	0.53	0.999
TAF5	SUS1	YBR198C	YBR111W-A	0	0	0	0	0	0.81	0.9	0.233	0.983
TAF5	SPT7	YBR198C	YBR081C	0	0	0	0	0.071	0.999	0.9	0.874	0.999
TAF6	CHD1	YGL112C	YER164W	0	0	0	0	0	0.814	0.9	0.102	0.98
TAF6	SGF29	YGL112C	YCL010C	0	0	0	0	0	0.999	0.9	0.092	0.999
TAF6	TAF5	YGL112C	YBR198C	0	0	0	0	0	0.999	0.948	0.872	0.999
TAF6	SPT7	YGL112C	YBR081C	0	0	0	0	0	0.999	0.9	0.729	0.999
TAF6	TAF10	YGL112C	YDR167W	0	0	0	0	0	0.999	0.948	0.81	0.999
TAF6	SUS1	YGL112C	YBR111W-A	0	0	0	0	0	0.992	0.9	0	0.999

TAF6	SPT3	YGL112C	YDR392W	0	0	0	0	0	0.999	0.9	0.771	0.999
TAF6	ADA2	YGL112C	YDR448W	0	0	0	0	0	0.999	0.9	0.728	0.999
TAF6	SGF73	YGL112C	YGL066W	0	0	0	0	0	0.999	0.9	0.069	0.999
TAF6	TAF12	YGL112C	YDR145W	0	0	0	0	0	0.999	0.948	0.777	0.999
TAF6	NGG1	YGL112C	YDR176W	0	0	0	0	0	0.999	0.9	0.772	0.999
TAF9	TRA1	YMR236W	YHR099W	0	0	0	0	0	0.999	0.948	0.777	0.999
TAF9	TAF10	YMR236W	YDR167W	0	0	0	0	0	0.999	0.948	0.81	0.999
TAF9	SGF29	YMR236W	YCL010C	0	0	0	0	0	0.999	0.9	0	0.999
TAF9	CHD1	YMR236W	YER164W	0	0	0	0	0	0.814	0.9	0	0.98
TAF9	UBP8	YMR236W	YMR223W	0	0	0	0	0	0.999	0.9	0.777	0.999
TAF9	NGG1	YMR236W	YDR176W	0	0	0	0	0	0.999	0.9	0.772	0.999
TAF9	SPT8	YMR236W	YLR055C	0	0	0	0	0	0.999	0.9	0.776	0.999
TAF9	SUS1	YMR236W	YBR111W-A	0	0	0	0	0	0.81	0.9	0	0.979
TAF9	ADA2	YMR236W	YDR448W	0	0	0	0	0	0.999	0.948	0.854	0.999
TAF9	SGF73	YMR236W	YGL066W	0	0	0	0	0	0.999	0.9	0.093	0.999
TAF9	SPT7	YMR236W	YBR081C	0	0	0	0	0	0.999	0.948	0.729	0.999
TAF9	SPT3	YMR236W	YDR392W	0	0	0	0	0	0.999	0.948	0.771	0.999
TAF9	TAF6	YMR236W	YGL112C	0	0	0	0	0	0.999	0.948	0.898	0.999
TAF9	GCN5	YMR236W	YGR252W	0	0	0	0	0	0.999	0.948	0.726	0.999
TAF9	TAF5	YMR236W	YBR198C	0	0	0	0	0	0.999	0.948	0.81	0.999
TAF9	TAF12	YMR236W	YDR145W	0	0	0	0	0	0.999	0.948	0.79	0.999
TRA1	SGF29	YHR099W	YCL010C	0	0	0	0	0	0.999	0.9	0.257	0.999
TRA1	SPT3	YHR099W	YDR392W	0	0	0	0	0	0.999	0.948	0.771	0.999
TRA1	GCN5	YHR099W	YGR252W	0	0	0	0	0	0.999	0.948	0.726	0.999
TRA1	SUS1	YHR099W	YBR111W-A	0	0	0	0	0	0.999	0.9	0.401	0.999
TRA1	TAF6	YHR099W	YGL112C	0	0	0	0	0	0.999	0.9	0.777	0.999
TRA1	CHD1	YHR099W	YER164W	0	0	0	0	0.109	0.814	0.9	0.1	0.981
TRA1	ADA2	YHR099W	YDR448W	0	0	0	0	0	0.999	0.9	0.728	0.999
TRA1	TAF5	YHR099W	YBR198C	0	0	0	0	0	0.999	0.9	0.777	0.999
TRA1	SGF73	YHR099W	YGL066W	0	0	0	0	0	0.999	0.9	0.187	0.999
TRA1	TAF10	YHR099W	YDR167W	0	0	0	0	0	0.999	0.948	0.777	0.999
TRA1	SPT7	YHR099W	YBR081C	0	0	0	0	0	0.999	0.948	0.729	0.999
TRA1	TAF12	YHR099W	YDR145W	0	0	0	0	0	0.999	0.948	0.777	0.999
TRA1	NGG1	YHR099W	YDR176W	0	0	0	0	0	0.999	0.9	0.772	0.999
UBP8	TRA1	YMR223W	YHR099W	0	0	0	0	0	0.998	0.9	0.777	0.999
UBP8	TAF12	YMR223W	YDR145W	0	0	0	0	0	0.999	0.9	0.777	0.999
UBP8	TAF5	YMR223W	YBR198C	0	0	0	0	0	0.999	0.9	0.777	0.999
UBP8	CHD1	YMR223W	YER164W	0	0	0	0	0	0.814	0.9	0.522	0.989
UBP8	NGG1	YMR223W	YDR176W	0	0	0	0	0	0.999	0.9	0.772	0.999
UBP8	GCN5	YMR223W	YGR252W	0	0	0	0	0	0.999	0.9	0.741	0.999
UBP8	SUS1	YMR223W	YBR111W-A	0	0	0	0	0	0.999	0.9	0.747	0.999
UBP8	ADA2	YMR223W	YDR448W	0	0	0	0	0	0.999	0.9	0.728	0.999
UBP8	SPT3	YMR223W	YDR392W	0	0	0	0	0	0.999	0.9	0.771	0.999
UBP8	SPT8	YMR223W	YLR055C	0	0	0	0	0	0.999	0.9	0.776	0.999
UBP8	TAF10	YMR223W	YDR167W	0	0	0	0	0	0.999	0.9	0.777	0.999
UBP8	SGF73	YMR223W	YGL066W	0	0	0	0	0	0.999	0.9	0.81	0.999
UBP8	SGF29	YMR223W	YCL010C	0	0	0	0	0	0.999	0.9	0.653	0.999
UBP8	TAF6	YMR223W	YGL112C	0	0	0	0	0	0.999	0.9	0.777	0.999
UBP8	SPT7	YMR223W	YBR081C	0	0	0	0	0	0.999	0.9	0.741	0.999
