# Supplementary material for: Spt-Ada-Gcn5-Acetyltransferase (SAGA) Complex in Plants: Genome Wide Identification, Evolutionary Conservation and Functional Determination
Source: PLoS One. 2015 Aug 11;10(8):e0134709. doi: 10.1371/journal.pone.0134709 (PMC4532415; doi:10.1371/journal.pone.0134709)
Supplement: S7 Table — (PDF) [file pone.0134709.s014.pdf]

S7 Table: MPSS data for *Arabidopsis* SAGA complex encoding genes showing different tissue-specific abundance.

LOCUS	Signature	CAF	INF	LEF	ROF	SIF	AP1	AP3	AGM	INS	ROS	SAP	LES	GSE	CAS	SIS
**At1g02680**	20	35	41	45	38	46	34	46	12	32	4	14	17	9	24	64
**Spt3**	17	31	37	39	34	40	38	41	10	26	17	12	17	11	20	50
**At1g04950**	20	34	142	48	24	128	40	50	14	0	6	0	28	0	28	62
**Taf6**	17	34	116	46	30	112	37	47	12	0	6	0	26	0	26	52
**At1g17440**	20	56	34	39	11	17	42	57	41	36	15	9	35	49	80	16
**Taf12b**	17	47	28	35	9	15	36	50	35	32	15	9	33	43	67	15
**At1g32750**	20	143	82	68	140	79	72	121	197	138	82	172	82	4	37	63
**Haf01/Spt7**	17	121	76	59	120	70	64	105	171	122	71	153	77	2	30	71
**At1g54140**	20	48	106	47	90	172	34	25	5	28	5	23	7	1	30	0
**Taf9**	17	41	96	44	88	159	30	21	5	26	5	20	7	3	42	4
**At1g54360**	20	0	0	0	0	0	17	16	7	6	36	12	6	0	98	27
**Taf6b**	17	0	0	0	0	0	15	19	6	6	35	11	6	0	82	37
**At1g72390**	20	24	154	68	27	39	14	6	12	41	4	18	22	0	14	2
**Spt20**	17	26	166	67	39	45	12	6	10	36	4	26	20	0	19	3
**At2g13370**	20	0	0	0	9	3	43	42	46	83	58	27	100	18	20	58
**Chr5**	17	0	2	4	10	4	41	35	46	82	54	28	95	11	30	56
**At2g14850**	20	74	54	28	17	49	19	9	11	23	49	32	0	0	50	0
**Ada1a**	17	64	48	24	14	48	17	9	10	20	44	30	0	0	39	0
**At2g17930**	20	4	0	0	0	0	23	30	0	13	10	4	14	24	0	0
**Tra1a**	17	4	0	0	0	0	19	24	0	14	9	6	22	18	0	0
**At3g10070**	20	0	4	16	0	0	0	0	0	0	0	6	0	4	8	0
**Taf12**	17	0	4	16	0	0	0	0	0	0	0	4	0	0	8	0
**At3g27100**	20	13	14	15	20	21	11	28	20	16	150	20	46	44	53	21
**Eny2/Sus1**	17	11	13	13	17	19	11	26	19	16	140	17	43	36	43	22
**At3g27460**	20	3	2	0	3	0	4	1	0	0	0	0	3	0	0	4
**Sgf29a**	17	8	10	0	4	3	9	3	10	0	6	0	5	0	0	3
**At3g54610**	20	48	0	0	0	24	0	5	0	0	0	0	0	0	0	0
**GCN5**	17	54	0	0	0	38	0	4	0	0	0	0	0	0	0	0
**At4g16420**	20	105	29	62	9	14	11	9	11	14	0	10	92	2	10	45
**Ada2b**	17	106	56	97	20	15	26	11	12	13	0	10	180	25	26	113
**At4g29790**	20	23	0	15	0	0	11	6	0	7	33	12	0	7	0	17
**Ada3**	17	4	0	0	0	0	0	0	0	0	0	0	0	1	0	0
**At4g31720**	20	134	184	232	306	260	88	167	48	127	560	59	74	5	312	194
**Taf10**	17	116	156	207	268	226	74	141	43	112	486	61	66	5	258	178
**At4g36080**	20	0	0	0	0	0	5	12	4	6	4	0	20	4	0	31
**Tra1b**	17	0	0	0	0	0	10	12	4	13	4	0	20	3	0	40
**At5g10790**	20	13	0	8	11	4	0	2	0	0	1	0	16	41	16	0
**Ubp22**	17	14	4	10	20	10	0	0	0	5	15	0	14	10	30	0
**At5g25150**	20	194	94	28	44	56	16	64	60	54	71	20	0	0	0	4
**Taf5**	17	174	102	38	36	58	20	68	68	60	83	20	0	4	0	6
**At5g40550**	20	10	8	12	26	38	14	22	14	16	4	5	0	0	0	4
**Sgf29b**	17	8	8	12	22	30	12	18	22	14	0	9	0	2	0	2
**At5g58575**	20	29	10	16	19	37	19	20	18	17	40	5	26	0	18	0
**Sgf11**	17	29	10	14	18	36	17	19	15	15	33	5	27	4	44	19
**At5g67410**	20	10	0	3	1	14	0	0	0	0	5	29	0	0	11	2
**Ada1b**	17	9	0	2	1	12	0	0	0	0	6	25	1	14	13	3

CAF Callus - actively growing, classic MPSS,
LEF Leaves - 21 day, untreated, classic MPSS,
SIF Silique - 24 to 48 hr post-fertilization, classic MPSS,
AP3 ap3-6 infloresence - mixed stage, immature buds,
INS Infloresence - mixed stage, immature buds,
SAP sup/ap1 infloresence - mixed stage, immature buds
GSE Germinating seedlings,
SIS Siliques - 24 to 48 hr post-fertilization, signature MPSS
INF Infloresence - mixed stage, immature buds, classic MPSS
ROF Root - 21 day, untreated, classic MPSS
AP1 ap1-10 infloresence - mixed stage, immature buds
AGM agamous infloresence - mixed stage, immature buds
ROS Root - 21 day, untreated
LES Leaves - 21 day, untreated
CAS Callus - actively growing, signature MPSS
