# Supplementary material for: Spt-Ada-Gcn5-Acetyltransferase (SAGA) Complex in Plants: Genome Wide Identification, Evolutionary Conservation and Functional Determination
Source: PLoS One. 2015 Aug 11;10(8):e0134709. doi: 10.1371/journal.pone.0134709 (PMC4532415; doi:10.1371/journal.pone.0134709)
Supplement: S8 Table — (PDF) [file pone.0134709.s015.pdf]

S8 Table: MPSS data for *O. sativa* SAGA complex encoding genes showing different tissue-specific abundance.

LOCUS	Signature	NYR	NRA	NRB	NGD	NST	NYL	NLA	NLB	NLC	NLD	NME	NPO	NOS	NIP	NGS	NCA
**LOC_Os01g02860**	20	0	0	0	3	0	0	0	0	0	0	12	0	0	0	0	0
**Spt20**	17	0	0	0	2	0	0	0	0	0	0	10	0	0	0	0	0
**LOC_Os01g23630**	20	69	69	9	0	0	45	18	2	0	2	29	84	182	67	78	121
**TAF13/Spt3**	17	82	56	10	66	32	33	18	0	28	10	28	70	198	73	106	82
**LOC_Os01g32750**	20	0	0	53	0	16	54	0	0	0	0	0	0	5	0	0	7
**Taf6**	17	0	0	44	0	40	46	0	29	0	2	0	0	24	0	0	26
**LOC_Os01g62820**	20	0	3	0	0	0	64	0	0	0	0	0	0	0	0	0	0
**Taf12b**	17	0	3	0	0	0	77	0	0	0	0	3	0	27	0	0	3
**LOC_Os01g63940**	20	0	0	0	0	0	0	0	0	0	7	0	0	0	0	0	24
**Taf12b**	17	0	0	0	0	0	0	0	0	0	0	0	0	0	0	0	19
**LOC_Os01g69110**	20	0	45	0	6	45	43	12	0	0	0	33	0	4	23	0	0
**Sus1**	17	2	44	0	12	31	32	8	5	0	0	25	0	4	49	3	21
**LOC_Os03g29470**	20	0	17	0	0	0	0	0	0	4	34	0	0	0	0	4	0
**Taf9**	17	2	40	19	0	0	41	0	7	3	43	0	0	64	18	3	64
**LOC_Os03g53960**	20	196	70	186	38	46	126	46	309	249	262	64	29	80	178	124	155
**Ada2b**	17	166	70	173	38	78	142	78	277	204	228	57	30	71	162	136	127
**LOC_Os03g55450**	20	9	0	82	0	6	0	24	198	141	133	56	49	73	156	0	36
**Ada1b**	17	21	23	64	22	9	21	39	162	115	108	60	44	64	135	14	59
**LOC_Os04g55360**	20	0	0	0	0	0	3	0	0	0	5	0	6	0	28	4	0
**Ubp22**	17	0	11	0	0	0	2	0	0	0	3	0	7	10	37	9	0
**LOC_Os05g28300**	20	0	20	0	0	25	0	0	0	14	0	21	0	0	0	0	0
**Ada3**	17	9	36	3	0	26	21	0	1	11	0	20	48	6	43	0	0
**LOC_Os05g28370**	20	There are no expressed signatures associated with LOC_Os05g28370															
**Sgf11**	17	0	0	0	0	0	0	0	0	0	0	0	0	0	0	0	0
**LOC_Os06g43790**	20	0	98	145	0	0	31	9	279	86	175	95	85	176	23	36	43
**Haf01/Spt7**	17	0	81	150	0	0	25	7	208	66	134	91	73	134	18	43	53
**LOC_Os06g44030**	20	3	5	28	0	22	24	42	22	15	0	57	0	87	17	0	68
**Taf5**	17	5	4	19	0	15	3	0	0	0	0	0	0	17	0	0	0
**LOC_Os07g42150**	20	0	0	0	0	0	0	0	0	0	0	0	0	0	0	0	0
**Taf9b**	17	0	0	0	0	0	0	0	0	0	0	0	0	0	0	0	0
**LOC_Os07g45064**	20	297	560	374	339	198	348	511	1131	424	525	785	228	2295	364	163	452
**Tra1**	17	218	440	297	499	148	275	393	865	329	419	627	188	1732	290	121	365
**LOC_Os09g26180**	20	75	90	119	57	242	293	274	142	226	38	158	78	316	219	55	322
**Taf10**	17	68	87	98	92	184	236	214	119	211	48	134	127	262	184	45	267
**LOC_Os10g28040**	20	0	0	0	0	0	0	0	0	0	0	0	0	0	0	0	0
**Gcn5**	17	0	0	0	0	0	0	0	0	0	0	0	0	0	0	0	0
**LOC_Os12g19350**	20	23	0	149	0	1	8	9	0	0	2	0	18	62	0	9	11
**Sgf29**	17	19	0	121	1	0	6	18	0	0	2	0	21	51	0	25	31
**LOC_Os12g39090**	20	0	0	17	0	0	0	0	0	0	0	0	0	15	0	0	0
**Ada1a**	17	0	0	18	4	0	1	0	0	0	0	2	2	12	0	0	0

NYR 14 days - Young Roots,
NRB 60 days - Mature Roots - Replicate B,
NST 60 days - Stem,
NLA 60 days - Mature Leaves - Replicate A ,
NLC 60 days - Mature Leaves - Replicate C,
NME 60 days - Crown vegetative meristematic tissue
NOS Ovary and mature stigma
NGS 3 days - Germinating seed
NRA 60 days - Mature Roots - Replicate A
NGD 10 days - Germinating seedlings grown in dark
NYL 14 days - Young leaves
NLB 60 days - Mature Leaves - Replicate B
NLD 60 days - Mature Leaves - Replicate D
NPO Mature Pollen,
NIP 90 days - Immature panicle,
NCA 35 days – Callus
