# Supplementary material for: Spt-Ada-Gcn5-Acetyltransferase (SAGA) Complex in Plants: Genome Wide Identification, Evolutionary Conservation and Functional Determination
Source: PLoS One. 2015 Aug 11;10(8):e0134709. doi: 10.1371/journal.pone.0134709 (PMC4532415; doi:10.1371/journal.pone.0134709)
Supplement: S9 Table — (PDF) [file pone.0134709.s016.pdf]

S9 Table: List of co-expression genes in *Arabidopsis*.

S.No.	Locus1		Locus2		MR (All)	MR (Tissue)	MR (Abiotic)	MR (Biotic)	MR (Hormone)
43	**At1g72390**		**At3g10070**	TAF12	1056.8	3177.3	450.3	3261.2	2811.9
44	**At4g16420**	ADA2B	**At4g31720**	TAF10	1075.7	2188.5	4541.6	2344.7	17.4
45	**At1g54140**	TAF9	**At2g17930**	TRA1A	1140.2	211.7	21441.6	14897.3	640
46	**At1g72390**		**At5g10790**	UBP22	1147	3321.4	584.1	3348.9	9222.1
47	**At4g16420**	ADA2B	**At5g58575**		1175.3	7785.6	920.7	1499.3	1762.8
48	**At4g29790**		**At5g58575**		1195.5	624.8	9594.9	1291	1966.4
49	**At1g04950**	TAF6	**At1g72390**		1331.1	2755.2	3837.1	4852.6	397.8
50	**At2g13370**	CHR5	**At2g17930**	TRA1A	1438.6	5119.3	7426.7	1606.5	133.9
51	**At1g02680**	TAF13	**At1g17440**	TAF12B	1490.5	1044.8	11102.1	4385.5	1770.7
52	**At1g54140**	TAF9	**At3g10070**	TAF12	1501	1855.4	6495.3	18679.8	1960.3
53	**At1g02680**	TAF13	**At3g54610**	GCN5	1517.4	512	3250.3	4148.3	13157.9
54	**At1g17440**	TAF12B	**At1g32750**	HAF01	1527	1880.8	8200.7	821.2	2976.9
55	**At1g04950**	TAF6	**At3g54610**	GCN5	1532.7	683.2	571.5	4620.3	10099.1
56	**At1g04950**	TAF6	**At5g58575**		1561.7	14556	490.7	964.7	2144.1
57	**At1g04950**	TAF6	**At1g54360**	TAF6B4	1596.4	2707	4842.9	3184.6	1643.6
58	**At3g10070**	TAF12	**At3g54610**	GCN5	1633.8	2121.7	7467.6	4182.5	1139.2
59	**At3g10070**	TAF12	**At5g10790**	UBP22	1762.1	13780	62.4	5011.8	13784.9
60	**At2g14850**		**At4g29790**		1771.9	441.9	1964.2	10415.7	19140
61	**At1g54140**	TAF9	**At4g16420**	ADA2B	1889.8	1243.2	4331.1	1841.1	1340.9
62	**At2g17930**	TRA1A	**At3g54610**	GCN5	2065.7	338.9	6722.4	2479.2	11210.9
63	**At1g54140**	TAF9	**At1g72390**		2070.5	3967.8	3070.9	5405.9	316.5
64	**At3g54610**	GCN5	**At4g16420**	ADA2B	2091.4	1710.1	5018.6	398.6	10305.8
65	**At1g04950**	TAF6	**At1g32750**	HAF01	2130.9	3833.8	3510.5	2725.4	19705.3
66	**At1g02680**	TAF13	**At1g54360**	TAF6B4	2202.6	2241.5	9594.7	2007.3	648.5
67	**At2g17930**	TRA1A	**At4g31720**	TAF10	2218.9	2717.3	13000.1	2358.4	357
68	**At1g54360**	TAF6B4	**At3g27100**		2373.5	3580	7606.3	489.4	1742.5
69	**At1g54140**	TAF9	**At2g14850**		2456.3	3630.5	6181.6	2305.5	12267.7
70	**At2g17930**	TRA1A	**At3g10070**	TAF12	2519.8	2225.7	2472.4	4844	5244
71	**At1g02680**	TAF13	**At4g29790**		2802.6	7589.2	3958	3889.1	556
72	**At1g04950**	TAF6	**At4g29790**		2965	11547.5	4488.5	9087.8	2271.8
73	**At3g27100**		**At4g16420**	ADA2B	3007.9	6549.6	3131.6	1428.2	6145.5
74	**At2g13370**	CHR5	**At5g10790**	UBP22	3164	1599.2	1813.4	5161.7	21397.9
75	**At1g54140**	TAF9	**At1g54360**	TAF6B4	3230.7	2291	4121.9	18659	1565.3
76	**At4g16420**	ADA2B	**At5g40550**		3244.4	2813.1	4415.3	8781.8	2825.6
77	**At2g13370**	CHR5	**At5g58575**		3248.4	3902.7	8219.8	1425.2	17800.4
78	**At1g72390**		**At5g58575**		3349.1	2736.4	7775.9	2403.8	13506.4
79	**At1g02680**	TAF13	**At5g58575**		3376.7	11474.3	699.6	104.4	188.1
80	**At1g17440**	TAF12B	**At1g54360**	TAF6B4	3456.8	4046.6	19058.5	750.4	1572.6
81	**At3g10070**	TAF12	**At4g16420**	ADA2B	3572.4	1738.7	1591.7	6684.9	8094.8
82	**At1g17440**	TAF12B	**At3g10070**	TAF12	3641.8	10591.4	1157.1	5715.2	2010.7
83	**At1g17440**	TAF12B	**At5g10790**	UBP22	3642.8	12151.9	1268.1	3072.4	21406.2
84	**At2g13370**	CHR5	**At2g14850**		3704.1	6221.4	465.6	2622.7	17498.2
85	**At2g17930**	TRA1A	**At5g40550**		3718.6	6512	1506.7	2875.2	13551.1
86	**At3g10070**	TAF12	**At4g31720**	TAF10	3746.7	2122.1	19512.2	6958	7126.3
87	**At2g13370**	CHR5	**At3g10070**	TAF12	3747.6	7678.1	884	2512	3531.5
88	**At2g14850**		**At3g54610**	GCN5	3748.2	4818.3	3790.2	3252.1	14056.6
89	**At1g72390**		**At4g31720**	TAF10	3755.1	5482.3	10768.4	5151.6	346.8
90	**At4g31720**	TAF10	**At5g58575**		3779.4	13586.6	2267.1	7.8	6255.3

91	**At1g54140**	TAF9	**At3g27100**		3807.9	2548.2	6256.2	14730.4	7072.7
92	**At4g29790**		**At5g10790**	UBP22	3819.5	1548.8	10561	3818.8	6219.9
93	**At1g72390**		**At5g40550**		3836.1	7835.7	1799.8	11166.8	15171.3
94	**At2g14850**		**At2g17930**	TRA1A	3838.9	1747	21300.7	2975.6	10137.9
95	**At1g32750**	HAF01	**At5g40550**		3890.1	8313.9	1934.6	4921.5	5648.2
96	**At2g13370**	CHR5	**At3g27100**		3919.3	10181.7	6059.9	335	7323
97	**At2g17930**	TRA1A	**At4g29790**		3954.2	7415.1	15071	3098.7	1674.3
98	**At2g17930**	TRA1A	**At3g27100**		3960.2	4267.6	14180.2	305.4	9076.8
99	**At3g27100**		**At5g58575**		3979.9	12609.9	2263.1	851.1	4984.8
100	**At1g04950**	TAF6	**At1g54140**	TAF9	4021.8	117.8	18025.9	18613.8	2393.9
101	**At1g54140**	TAF9	**At5g40550**		4262.7	4588.1	9902.9	10823.8	18280.6
102	**At1g54360**	TAF6B4	**At4g31720**	TAF10	4305.9	8233.5	4415.6	2749.6	618.4
103	**At1g02680**	TAF13	**At3g10070**	TAF12	4312.3	3202.5	10864.3	2754.9	6056.2
104	**At1g02680**	TAF13	**At2g13370**	CHR5	4321.1	12073.2	7769.8	375	5097.2
105	**At3g27100**		**At3g54610**	GCN5	4357.1	1671.7	6430.5	1328.9	14953.4
106	**At3g54610**	GCN5	**At4g31720**	TAF10	4376.7	2498.7	4974.5	9497.3	14250.4
107	**At1g17440**	TAF12B	**At3g54610**	GCN5	4486.7	5489.7	15910.5	2894.9	5267.2
108	**At1g72390**		**At3g54610**	GCN5	4544.8	6174.7	7313.5	2093.4	4155.4
109	**At5g10790**	UBP22	**At5g58575**		4648.6	665	4898	10566	7433.2
110	**At1g32750**	HAF01	**At3g10070**	TAF12	4691.2	5010.4	760.6	3436.1	11187.5
111	**At1g32750**	HAF01	**At3g54610**	GCN5	4708.6	6331.9	2130.6	4589.1	3945
112	**At1g17440**	TAF12B	**At1g54140**	TAF9	4794.9	6853.8	15122.8	4872.4	295.7
113	**At1g04950**	TAF6	**At5g40550**		4814.8	3885.4	5170.3	4267.5	3947.8
114	**At1g54360**	TAF6B4	**At4g16420**	ADA2B	4967.6	6703.3	12344.9	5335.5	1138.5
115	**At4g16420**	ADA2B	**At5g10790**	UBP22	4980.1	10884.9	2305	5982.3	13056.8
116	**At2g14850**		**At3g10070**	TAF12	5225	7049.9	9878.4	8468.3	9665.9
117	**At1g32750**	HAF01	**At1g54140**	TAF9	5238.2	3994.8	9817	10433	10915
118	**At1g32750**	HAF01	**At3g27100**		5375	6006	14507.1	2866.5	4446.8
119	**At2g13370**	CHR5	**At3g54610**	GCN5	5532.8	10860	2476.2	2348.9	5965.1
120	**At1g32750**	HAF01	**At5g10790**	UBP22	5771.3	7107.8	5109.4	9222.4	16902.4
121	**At1g32750**	HAF01	**At4g31720**	TAF10	5843.1	4663.6	7825.5	3109.6	17931.9
122	**At1g04950**	TAF6	**At2g13370**	CHR5	5853.1	11766.7	11588.2	5274.1	12853.7
123	**At1g04950**	TAF6	**At3g10070**	TAF12	5885.1	929.8	13156.2	4463.3	7600.5
124	**At1g04950**	TAF6	**At2g14850**		5906.7	3019.7	12191.5	7894.6	14107.2
125	**At1g54360**	TAF6B4	**At3g10070**	TAF12	5978.7	7297.9	6413.5	1551.3	4132.7
126	**At4g31720**	TAF10	**At5g40550**		6084.3	1887.7	10971.6	19028.5	16785.9
127	**At2g14850**		**At3g27100**		6694.2	7468.7	8202.4	4007.6	9894.9
128	**At1g04950**	TAF6	**At3g27100**		6830.1	8716.5	5061.9	2360.5	19061.3
129	**At2g17930**	TRA1A	**At5g58575**		6981.2	13336.8	852.4	1867.3	18378.1
130	**At1g32750**	HAF01	**At1g54360**	TAF6B4	7101.5	16103.4	859.9	3823.3	5452.1
131	**At3g54610**	GCN5	**At5g40550**		7163.9	5889.1	8837.1	11196.4	18156.1
132	**At2g14850**		**At5g58575**		7505.5	3229.1	12156.3	2887.3	15309.9
133	**At2g13370**	CHR5	**At4g31720**	TAF10	7741.2	10675.9	22128.3	1024.6	3271.2
134	**At1g04950**	TAF6	**At1g17440**	TAF12B	7883.9	5410	18056.6	10341.4	6445.2
135	**At1g02680**	TAF13	**At5g40550**		7935.9	9559.2	10303.4	8254.3	1542.9
136	**At2g14850**		**At4g31720**	TAF10	8412.4	4027.9	16101.9	2895.3	15955.6
137	**At3g27100**		**At5g40550**		8841.5	8222.9	11837.2	11362.5	5388.4
138	**At1g54360**	TAF6B4	**At5g40550**		8906.4	14125.6	2284.8	5117.6	7160.5
139	**At3g27100**		**At4g29790**		9265.8	12560.3	8933.9	6493.8	17394.1
140	**At5g40550**		**At5g58575**		9360.1	10485.6	2129.9	13255.5	11896.7
141	**At1g17440**	TAF12B	**At5g58575**		9689.1	6644.9	10859.3	5542.1	12085.3
142	**At3g54610**	GCN5	**At4g29790**		9696.7	14439.2	7849.8	10191.6	3400.9

143	**At4g29790**		**At4g31720**	TAF10	9793	12587	13821.5	7385.3	3094.4
144	**At1g17440**	TAF12B	**At3g27100**		9873.5	9356.2	17193.1	3086.9	14415.6
145	**At1g72390**		**At3g27100**		10071.3	9812.6	14895	2043	19734.2
146	**At2g13370**	CHR5	**At5g40550**		10346.8	3931	13280	12883.5	19281.8
147	**At2g17930**	TRA1A	**At5g10790**	UBP22	10771.3	16800.7	5130.6	6095.1	18944.2
148	**At1g54360**	TAF6B4	**At1g72390**		10773	15791.2	10724.9	3134.8	3148.2
149	**At1g54360**	TAF6B4	**At2g13370**	CHR5	11099.4	19238.6	16694.5	2442.2	1993.6
150	**At3g27100**		**At5g10790**	UBP22	11165.1	13797.3	11742.4	2310.8	13092.8
151	**At1g54140**	TAF9	**At2g13370**	CHR5	11905.2	15111.4	18873.3	11297.7	964.3
152	**At1g54140**	TAF9	**At4g29790**		12772.1	14337.5	14951.3	2654	1778.2
153	**At2g14850**		**At5g10790**	UBP22	13031.3	10619.5	11821.6	16446.8	19427.3
154	**At2g14850**		**At5g40550**		13228	13661.5	17455.6	11868.5	547.2
155	**At1g54360**	TAF6B4	**At2g14850**		14298.8	12267.2	18444	7935	11624.4
156	**At3g10070**	TAF12	**At4g29790**		14723.6	11886.2	17077.6	20380.2	3455.6
157	**At1g17440**	TAF12B	**At5g40550**		14776.6	20624.4	9277.2	6549.1	17356.5
158	**At1g54360**	TAF6B4	**At5g58575**		14937.3	19917.6	6379.5	3471.5	12674.1
159	**At3g10070**	TAF12	**At3g27100**		15303.1	12231	19894	3322.3	18092.3
160	**At4g29790**		**At5g40550**		15569.4	12957	15340.7	11427.4	13896.7
161	**At1g02680**	TAF13	**At5g10790**	UBP22	15648.4	18698.6	15654.5	5465.7	15914.5
162	**At1g17440**	TAF12B	**At4g31720**	TAF10	16344.4	15403.8	22616.2	7331.4	1717.3
163	**At1g54140**	TAF9	**At5g10790**	UBP22	16470.6	20083	10932	13833.9	15515.4
164	**At1g54360**	TAF6B4	**At5g10790**	UBP22	17315	21010.3	13178.6	2149.4	22712
165	**At1g04950**	TAF6	**At5g10790**	UBP22	17328.7	19247	14768.1	11680.9	13632.9
166	**At3g54610**	GCN5	**At5g10790**	UBP22	17670	20398.3	18611.2	4380	5140.5
167	**At1g54360**	TAF6B4	**At4g29790**		18137.7	19283.2	21084.6	11704.1	8997.6
168	**At3g54610**	GCN5	**At5g58575**		18783.1	19223.5	6420	5987.1	19339.3
169	**At1g54140**	TAF9	**At5g58575**		19236.4	15966.1	15601.9	15513.4	14085.2
170	**At3g10070**	TAF12	**At5g58575**		19725.7	16208.3	5683.2	9919.2	17563
171	**At4g31720**	TAF10	**At5g10790**	UBP22	20462.2	16238.5	21426.9	14806.4	19161.3
