# Supplementary material for: Spt-Ada-Gcn5-Acetyltransferase (SAGA) Complex in Plants: Genome Wide Identification, Evolutionary Conservation and Functional Determination
Source: PLoS One. 2015 Aug 11;10(8):e0134709. doi: 10.1371/journal.pone.0134709 (PMC4532415; doi:10.1371/journal.pone.0134709)
Supplement: S10 Table — (PDF) [file pone.0134709.s017.pdf]

S10 Table: List of genes obtained from ATTED-II for *Arabidopsis* SAGA complex co-expressed gene network analysis.

S.N.	Locus	Target*	Function
1	At3g15351	O,M	
2	At1g70620	O,N	cyclin-related
3	At3g01320	M,N	SIN3-like 1
4	At1g35470	O,Y	SPla/RYanodine receptor (SPRY) domain-containing protein
5	At4g05000	O,N_P	Vacuolar protein sorting-associated protein VPS28 family protein
6	At1g03190	O,Y	RAD3-like DNA-binding helicase protein
7	At3g19870	O,P	
8	At5g55310	O,N	DNA topoisomerase 1 beta
9	At1g15440	O,Y	periodic tryptophan protein 2
10	At3g03880	C,N	Protein of unknown function (DUF1639)
11	At1g02890	C,C	AAA-type ATPase family protein
12	At1g24706	O,N	THO2
13	At4g31160	O,N	DDB1-CUL4 associated factor 1
14	At5g32470	M,C	Haem oxygenase-like, multi-helical
15	At3g07650	O,N	CONSTANS-like 9
16	At5g01400	M,N	HEAT repeat-containing protein
17	At5g49930	O,Y	zinc knuckle (CCHC-type) family protein
18	At3g01610	M,N	cell division cycle 48C
19	At2g47330	M,N	P-loop containing nucleoside triphosphate hydrolases superfamily protein
20	At5g03770	M,C	KDO transferase A
21	At3g12480	O,N	nuclear factor Y, subunit C11
22	At5g14080	M,C	Tetratricopeptide repeat (TPR)-like superfamily protein
23	At2g33340	O,Y	MOS4-associated complex 3B
24	At1g52740	O,N	histone H2A protein 9
25	At1g79280	O,N	nuclear pore anchor
26	At2g41710	O,N	Integrase-type DNA-binding superfamily protein
27	At5g43900	O,S	myosin 2
28	At1g58230	C,N	binding
29	At2g17670	C,C	Tetratricopeptide repeat (TPR)-like superfamily protein
30	At1g03060	C,P	Beige/BEACH domain ;WD domain, G-beta repeat protein
31	At4g11560	O,N	bromo-adjacent homology (BAH) domain-containing protein
32	At4g21430	O,N	Zinc finger, RING-type;Transcription factor jumonji/aspartyl beta-hydroxylase
33	At1g05380	C,N	Acyl-CoA N-acyltransferase with RING/FYVE/PHD-type zinc finger protein
34	At1g71010	O,N	FORMS APLOID AND BINUCLEATE CELLS 1C
35	At2g28290	O,N	P-loop containing nucleoside triphosphate hydrolases superfamily protein
36	At5g23330	M,C	Nucleotidylyl transferase superfamily protein
37	At3g58530	O,N	RNI-like superfamily protein
38	At1g67500	O,N	recovery protein 3
39	At2g25660	C,C	embryo defective 2410

40	At5g19660	M,P	SITE-1 protease
41	At3g51120	O,N	DNA binding;zinc ion binding;nucleic acid binding;nucleic acid binding
42	At1g64570	O,N	Homeodomain-like superfamily protein
43	At1g09730	O,N	Cysteine proteinases superfamily protein
44	At1g16610	C,N	arginine/serine-rich 45
45	At2g28240	O,N	ATP-dependent helicase family protein
46	At4g37120	O,N	Pre-mRNA splicing Prp18-interacting factor
47	At5g02850	C,C	hydroxyproline-rich glycoprotein family protein
48	At2g47410	O,N	WD40/YVTN repeat-like-containing domain;Bromodomain
49	At5g48250	O,N	B-box type zinc finger protein with CCT domain
50	At2g24765	O,C	ADP-ribosylation factor 3
51	At1g65660	O,N	Pre-mRNA splicing Prp18-interacting factor
52	At5g61140	M,C	U5 small nuclear ribonucleoprotein helicase
53	At5g08190	O,C	nuclear factor Y, subunit B12
54	At5g06240	O,C	embryo defective 2735
55	At3g22380	M,N	time for coffee
56	At5g59830	O,N	
57	At3g15120	O,N	P-loop containing nucleoside triphosphate hydrolases superfamily protein
58	At5g60960	C,C	Pentatricopeptide repeat (PPR) superfamily protein
59	At5g23090	O,C	nuclear factor Y, subunit B13
60	At3g49100	O,C	Signal recognition particle, SRP9/SRP14 subunit
61	At1g73240	O,C	
62	At5g55600	O,N	agenet domain-containing protein / bromo-adjacent homology (BAH) domain-containing protein
63	At2g47990	O,N	transducin family protein / WD-40 repeat family protein
64	At1g16710	M,N	histone acetyltransferase of the CBP family 12
65	At5g62440	O,N	Protein of unknown function (DUF3223)
66	At2g37080	C,N	ROP interactive partner 3
67	At1g20300	M,C	Pentatricopeptide repeat (PPR) superfamily protein
68	At2g29020	O,E	Rab5-interacting family protein
69	At5g61380	O,N	CCT motif -containing response regulator protein
70	At4g15520	O,Y	tRNA/rRNA methyltransferase (SpoU) family protein
71	At3g54230	O,N	suppressor of abi3-5
72	At2g45200	O,G	golgi snare 12
73	At5g16680	M,N	RING/FYVE/PHD zinc finger superfamily protein
74	At4g03120	O,N	C2H2 and C2HC zinc fingers superfamily protein
75	At5g50410	O,N	
76	At5g16780	O,N	SART-1 family
77	At3g19670	O,N	pre-mRNA-processing protein 40B
78	At1g44780	O,N	
79	At4g28990	O,N	RNA-binding protein-related
80	At4g21710	O,N	DNA-directed RNA polymerase family protein
81	At1g17110	S,	ubiquitin-specific protease 15

82	At2g43810	O,Y	Small nuclear ribonucleoprotein family protein
83	At2g41500	C,N	WD-40 repeat family protein / small nuclear ribonucleoprotein Prp4p-related
84	At2g33440	O,K	RNA-binding (RRM/RBD/RNP motifs) family protein
85	At5g12400	O,N	DNA binding;zinc ion binding;DNA binding
86	At3g22980	O,Y	Ribosomal protein S5/Elongation factor G/III/V family protein
87	At1g77800	O,N	PHD finger family protein
88	At1g06470	O,P	Nucleotide/sugar transporter family protein
89	At5g39600	O,C	
90	At1g80020		transposable element gene
91	At3g45830	O,N	
92	At1g21560	O,N	
93	At1g75140	S,P	
94	At1g48550	C,M	Vacuolar protein sorting-associated protein 26
95	At2g39260	O,N	binding;RNA binding
96	At2g03150	M,N	ATP/GTP-binding protein family
97	At5g55040	M,N	DNA-binding bromodomain-containing protein
98	At3g13222	O,N	GBF-interacting protein 1
99	At1g02750	O,N	Drought-responsive family protein
100	At5g23110	O,Y	Zinc finger, C3HC4 type (RING finger) family protein
101	At5g10710	O,N	
102	At1g28050	O,N	B-box type zinc finger protein with CCT domain
103	At1g09280	O,N	
104	At4g32910	O,N	
105	At5g15680	M,N	ARM repeat superfamily protein
106	At3g32940	C,N	RNA-binding KH domain-containing protein
107	At5g23610	O,N	
108	At3g06400	O,Y	chromatin-remodeling protein 11
109	At5g13010	O,N	RNA helicase family protein
110	At1g24190	O,N	SIN3-like 3
111	At3g07080	S,P	EamA-like transporter family
112	At1g01040	O,N	dicer-like 1
113	At3g61620	O,Y	3'-5'-exoribonuclease family protein
114	At5g14530	O,Y	Transducin/WD40 repeat-like superfamily protein
115	At1g12830	O,N	
116	At1g09770	O,Y	cell division cycle 5
117	At3g58660	C,Y	Ribosomal protein L1p/L10e family
118	At1g10270	M,C	glutamine-rich protein 23
119	At2g26920	C,N	Ubiquitin-associated/translation elongation factor EF1B protein
120	At1g28420	O,N	homeobox-1
121	At5g58040	O,N	homolog of yeast FIP1 [V]
122	At1g18450	O,N	actin-related protein 4
123	At2g04540	M,C	Beta-ketoacyl synthase
124	At5g66360	M,C	Ribosomal RNA adenine dimethylase family protein

125	At3g12550	O,Y	XH/XS domain-containing protein
126	At3g13190	O,Y	Plant protein of unknown function (DUF827)
127	At2g45640	M,Y	SIN3 associated polypeptide P18
128	At4g13160	S,P	Protein of unknown function, DUF593
129	At1g71350	O,Y	eukaryotic translation initiation factor SUI1 family protein
130	At3g06960	O,N	pigment defective 320
131	At3g12980	M,N	histone acetyltransferase of the CBP family 5
132	At5g43670	O,N	Sec23/Sec24 protein transport family protein
133	At1g76860	O,Y	Small nuclear ribonucleoprotein family protein
134	At1g27430	O,N	GYF domain-containing protein
135	At1g43850	C,N	SEUSS transcriptional co-regulator
136	At1g80010	C,N	FAR1-related sequence 8
137	At1g64750	O,N	deletion of SUV3 suppressor 1(I)
138	At1g48090	S,N	calcium-dependent lipid-binding family protein
139	At3g51500	C,Y_N	
140	At1g27900	O,N	RNA helicase family protein
141	At1g19370	S,P	
142	At3g49490	O,N	
143	At5g13480	O,N	Transducin/WD40 repeat-like superfamily protein
144	At3g08850	O,N	HEAT repeat ;WD domain, G-beta repeat protein protein
145	At5g25060	O,N	RNA recognition motif (RRM)-containing protein
146	At1g80070	C,Y	Pre-mRNA-processing-splicing factor
147	At1g48430	O,Y	Dihydroxyacetone kinase
148	At2g35330	S,C	RING/U-box superfamily protein
149	At3g02260	O,N	auxin transport protein (BIG)
150	At4g02560	O,N	Homeodomain-like superfamily protein
151	At5g67320	S,N	WD-40 repeat family protein
152	At3g50380	M,P	Protein of unknown function (DUF1162)
153	At1g61870	M,C	pentatricopeptide repeat 336
154	At3g27260	O,N	global transcription factor group E8
155	At5g22450	C,N	
156	At3g44680	O,Y	histone deacetylase 9
157	At3g22660	O,N	rRNA processing protein-related
158	At3g51620	O,N	PAP/OAS1 substrate-binding domain superfamily
159	At5g56240	C,N	
160	At1g59820	M,P	aminophospholipid ATPase 3
161	At3g01770	O,N	bromodomain and extraterminal domain protein 10
162	At4g31200	M,N	SWAP (Suppressor-of-White-APricot)/surp RNA-binding domain-containing protein
163	At1g50660	C,N	
164	At2g01060	O,N	myb-like HTH transcriptional regulator family protein
165	At1g07830	M,C	ribosomal protein L29 family protein
166	At5g10490	C,C	MSCS-like 2

167	At5g55300	O,N	DNA topoisomerase I alpha
168	At5g66180	O,Y	S-adenosyl-L-methionine-dependent methyltransferases superfamily protein
169	At1g02080	C,Y	transcription regulators
170	At2g44850	O,Y	
171	At3g57300	O,N	INO80 ortholog
172	At5g42420	O,V	Nucleotide-sugar transporter family protein
173	At1g20960	M,Y	U5 small nuclear ribonucleoprotein helicase, putative
174	At1g05120	O,N	Helicase protein with RING/U-box domain
175	At3g10650	O,N	
176	At5g45010	O,Y	DSS1 homolog on chromosome V
177	At3g06940		transposable element gene
178	At1g60200	C,N	splicing factor PWI domain-containing protein / RNA recognition motif (RRM)-containing protein
179	At5g63280	S,P	C2H2-like zinc finger protein
180	At5g64730	O,Y	Transducin/WD40 repeat-like superfamily protein
181	At3g28730	O,N	high mobility group

*C-chloroplast; E-Endoplasmic reticulum; G-golgi bodies; K-cytoskeletal; L-lysosome; M-mitochondria; N-nuclear; P-plasma membrane; S-extracellular; V-vacuole; X-peroxisome; Y-cytoplasm
