# Supplementary material for: Spt-Ada-Gcn5-Acetyltransferase (SAGA) Complex in Plants: Genome Wide Identification, Evolutionary Conservation and Functional Determination
Source: PLoS One. 2015 Aug 11;10(8):e0134709. doi: 10.1371/journal.pone.0134709 (PMC4532415; doi:10.1371/journal.pone.0134709)
Supplement: S11 Table — (PDF) [file pone.0134709.s018.pdf]

S11 Table: Analysis of cis-regulatory element in 1000bp upstream promoter sequences from TSS in SAGA complex subunit genes using PlantCARE and PLACE database.

S. No.	GENE	Locus	Site	Position from TSS	Strand	Element Sequence	Function
1	TAF13	**AT1G02680**	HSE	288	-	AGAAAATTCG	cis-acting element involved in heat stress responsiveness
			MBS	436	-	CAACTG	MYB binding site involved in drought-inducibility
				954	-	CAACTG	MYB binding site involved in drought-inducibility
				795	+	CAACTG	MYB binding site involved in drought-inducibility
			TC-rich repeats	301	+	ATTTTCTTCA	cis-acting element involved in defense and stress responsiveness
				548	+	ATTTTCTCCA	cis-acting element involved in defense and stress responsiveness
2	TAF6	**AT1G04950**	ABRE	671	-	TACGTG	cis-acting element involved in the abscisic acid responsiveness
			HSE	849	+	AAAAAATTTC	cis-acting element involved in heat stress responsiveness
			LTR	916	+	CCGAAA	cis-acting element involved in low-temperature responsiveness
			TC-rich repeats	746	-	ATTTTCTTCA	cis-acting element involved in defense and stress responsiveness
3	TAF12 b	**AT1G17440**	HSE	3	+	AAAAAATTTC	cis-acting element involved in heat stress responsiveness
			MBS	420	-	TAACTG	MYB binding site involved in drought-inducibility
				834	-	CAACTG	MYB binding site involved in drought-inducibility
4	TAF1	**AT1G32750**	HSE	190	+	AGAAAATTCG	cis-acting element involved in heat stress responsiveness
			MBS	492	-	AAAAAATTTC	cis-acting element involved in heat stress responsiveness
				717	-	TAACTG	MYB binding site involved in drought-inducibility
			TC-rich repeats	300	+	ATTTTCTCCA	cis-acting element involved in defense and stress responsiveness
				585	-	ATTTTCTTCA	cis-acting element involved in defense and stress responsiveness
				447	+	ATTTTCTTCA	cis-acting element involved in defense and stress responsiveness
5	TAF9	**AT1G54140**	ABRE	364	+	GCCACGTACA	cis-acting element involved in the abscisic acid responsiveness
			LTR	463	-	CCGAAA	cis-acting element involved in low-temperature responsiveness
			MBS	776	+	CGGTCA	MYB Binding Site
			TC-rich repeats	599	+	ATTTTCTTCA	cis-acting element involved in defense and stress responsiveness
6	TAF6b	**AT1G54360**	MBS	386	-	TAACTG	MYB binding site involved in drought-inducibility
			TC-rich repeats	877	+	ATTTTCTTCA	cis-acting element involved in defense and stress responsiveness
7	SPT20	**AT1G72390**	LTR	547	-	CCGAAA	cis-acting element involved in low-temperature responsiveness
				85	-	CCGAAA	cis-acting element involved in low-temperature responsiveness
			MBS	115	-	TAACTG	MYB binding site involved in drought-inducibility
8	CHR5	**AT2G13370**	MBS	721	-	CAACTG	MYB binding site involved in drought-inducibility
			TC-rich repeats	971	+	ATTTTCTCCA	cis-acting element involved in defense and stress responsiveness

9	ADA1A	**AT2G14850**	ABRE	153	-	CGCACGTGTC	cis-acting element involved in the abscisic acid responsiveness
				66	+	CGTACGTGCA	cis-acting element involved in the abscisic acid responsiveness
				155	-	CACGTG	cis-acting element involved in the abscisic acid responsiveness
				68	+	TACGTG	cis-acting element involved in the abscisic acid responsiveness
			HSE	659	+	AAAAAATTTC	cis-acting element involved in heat stress responsiveness
				162	-	AAAAAATTTC	cis-acting element involved in heat stress responsiveness
				211	-	AAAAAATTTC	cis-acting element involved in heat stress responsiveness
				161	-	AGAAAATTCG	cis-acting element involved in heat stress responsiveness
			MBS	408	-	TAACTG	MYB binding site involved in drought-inducibility
			TC-rich repeats	377	-	GTTTTCTTAC	cis-acting element involved in defense and stress responsiveness
				61	+	GTTTTCTTAC	cis-acting element involved in defense and stress responsiveness
10	TRA1A	**AT2G17930**	LTR	620	+	CCGAAA	cis-acting element involved in low-temperature responsiveness
			PRECONSCRHSP70A	929	-	SCGAYNRNNNNNNNNNNNNNNNHD	Involved in induction of HSP70A gene S=G/C; Y=C/T; R=A/G; H=T/C/A; D=A/T/G
				919	-	SCGAYNRNNNNNNNNNNNNNNNHD	Involved in induction of HSP70A gene S=G/C; Y=C/T; R=A/G; H=T/C/A; D=A/T/G
11	TAF12	**AT3G10070**	LTR	917	-	CCGAAA	cis-acting element involved in low-temperature responsiveness
				950	-	CCGAAA	cis-acting element involved in low-temperature responsiveness
			TC-rich repeats	300	-	ATTTTCTTCA	cis-acting element involved in defense and stress responsiveness
				581	+	ATTTTCTCCA	cis-acting element involved in defense and stress responsiveness
			PRECONSCRHSP70A	943		SCGAYNRNNNNNNNNNNNNNNNHD	Involved in induction of HSP70A gene S=G/C; Y=C/T; R=A/G; H=T/C/A; D=A/T/G
				14		SCGAYNRNNNNNNNNNNNNNNNHD	Involved in induction of HSP70A gene S=G/C; Y=C/T; R=A/G; H=T/C/A; D=A/T/G
12	ENY2	**AT3G27100**	ABRE	876	-	TACGTG	cis-acting element involved in the abscisic acid responsiveness
				943	-	TACGTG	cis-acting element involved in the abscisic acid responsiveness
			LTR	920	-	CCGAAA	cis-acting element involved in low-temperature responsiveness
			MBS	419	-	TAACTG	MYB binding site involved in drought-inducibility
			TC-rich repeats	68	-	ATTTTCTTCA	cis-acting element involved in defense and stress responsiveness
				557	+	ATTTTCTTCA	cis-acting element involved in defense and stress responsiveness
				494	+	ATTTTCTCCA	cis-acting element involved in defense and stress responsiveness
13	SGF29a	**AT3G27460**	ABRE	398	+	CACGTG	cis-acting element involved in the abscisic acid responsiveness
				399	+	ACGTGGC	cis-acting element involved in the abscisic acid responsiveness
			HSE	646	+	AAAAAATTTC	cis-acting element involved in heat stress responsiveness
			TC-rich repeats	125	+	ATTTTCTTCA	cis-acting element involved in defense and stress responsiveness
				242	-	ATTTTCTTCA	cis-acting element involved in defense and stress

							responsiveness
14	GCN5	**AT3G54610**	LTR	125	+	CCGAAA	cis-acting element involved in low-temperature responsiveness
			MBS	775	+	CGGTCA	MYB Binding Site
			CCAATBOX1	194	-	CCAAT	Cooperatively with HSEs to increase the hs promoter activity
			PRECONSCRHSP70A	754	-	SCGAYNRNNNNNNNNNNNNNNNHD	Involved in induction of HSP70A gene S=G/C; Y=C/T; R=A/G; H=T/C/A; D=A/T/G
				179	-	SCGAYNRNNNNNNNNNNNNNNNHD	Involved in induction of HSP70A gene S=G/C; Y=C/T; R=A/G; H=T/C/A; D=A/T/G
15	ADA2B	**AT4G16420**	HSE	17	-	AAAAAATTTC	cis-acting element involved in heat stress responsiveness
				860	+	AAAAAATTTC	cis-acting element involved in heat stress responsiveness
			TC-rich repeats	597	-	ATTTTCTTCA	cis-acting element involved in defense and stress responsiveness
				896	+	ATTTTCTTCA	cis-acting element involved in defense and stress responsiveness
			MYB1AT	561	+	WAACCA	MYB binding site involved in drought-inducibility W=A/T
				643	+	WAACCA	MYB binding site involved in drought-inducibility W=A/T
16	ADA3	**AT4G29790**	HSE	930	+	AAAAAATTTC	cis-acting element involved in heat stress responsiveness
			MBS	311	+	GTTTTCTTAC	cis-acting element involved in defense and stress responsiveness
			ABRELATERD1	347	-	ACGTG	early responsive to dehydration
17	TAF10	**AT4G31720**	MBS	987	-	CGGTCA	MYB Binding Site
			TC-rich repeats	371	-	ATTTTCTCCA	cis-acting element involved in defense and stress responsiveness
				515	-	ATTTTCTCCA	cis-acting element involved in defense and stress responsiveness
			CCAATBOX1	529	+	CCAAT	Cooperatively with HSEs to increase the hs promoter activity
			MYB1AT	438	+	WAACCA	MYB binding site involved in drought-inducibility W=A/T
18	TRA1B	**AT4G36080**	HSE	716	-	AGAAAATTCG	cis-acting element involved in heat stress responsiveness
			MBS	543	+	CGGTCA	MYB Binding Site
				712	-	CGGTCA	MYB Binding Site
19	UBP22	**AT5G10790**	HSE	499	-	AAAAAATTTC	cis-acting element involved in heat stress responsiveness
				33	+	AAAAAATTTC	cis-acting element involved in heat stress responsiveness
				265	-	AAAAAATTTC	cis-acting element involved in heat stress responsiveness
			LTR	39	-	CCGAAA	cis-acting element involved in low-temperature responsiveness
			MBS	703	-	TAACTG	MYB binding site involved in drought-inducibility
				694	+	TAACTG	MYB binding site involved in drought-inducibility
			TC-rich repeats	504	+	ATTTTCTCCA	cis-acting element involved in defense and stress responsiveness
20	TAF5	**AT5G25150**	HSE	841	+	AAAAAATTTC	cis-acting element involved in heat stress responsiveness
				715	-	AAAAAATTTC	cis-acting element involved in heat stress responsiveness
				814	+	AAAAAATTTC	cis-acting element involved in heat stress responsiveness
				815	+	AAAAAATTTC	cis-acting element involved in heat stress

							responsiveness
				774	+	AAAAAATTTC	cis-acting element involved in heat stress responsiveness
			MBS	525	-	TAACTG	MYB binding site involved in drought-inducibility
21	SGF29 B	**AT5G40550**	HSE	651	-	AAAAAATTTC	cis-acting element involved in heat stress responsiveness
				367	-	AAAAAATTTC	cis-acting element involved in heat stress responsiveness
			MBS	237	+	TAACTG	MYB binding site involved in drought-inducibility
			TC-rich repeats	435	-	GTTTTCTTAC	cis-acting element involved in defense and stress responsiveness
22	SFG11	**AT5G58575**	ABRE	551	-	TACGTG	cis-acting element involved in the abscisic acid responsiveness
			TC-rich repeats	522	+	ATTTTCTTCA	cis-acting element involved in defense and stress responsiveness
				104	-	GTTTTCTTAC	cis-acting element involved in defense and stress responsiveness
			CCAATB OX1	571	+	CCAAT	Cooperatively with HSEs to increase the hs promoter activity
23	ADA1B	**AT5G67410**	ABRE	419	+	CACGTG	cis-acting element involved in the abscisic acid responsiveness
			HSE	480	+	AAAAAATTTC	cis-acting element involved in heat stress responsiveness
				885	-	AAAAAATTTC	cis-acting element involved in heat stress responsiveness
