# Supplementary material for: Spt-Ada-Gcn5-Acetyltransferase (SAGA) Complex in Plants: Genome Wide Identification, Evolutionary Conservation and Functional Determination
Source: PLoS One. 2015 Aug 11;10(8):e0134709. doi: 10.1371/journal.pone.0134709 (PMC4532415; doi:10.1371/journal.pone.0134709)
Supplement: S2 Fig — SAGA complex subunit protein sequences were used from At, A. thaliana (red circle); Dm, D. melongaster; Hs, H. sapiens (red square); Os, O. sativa (red diamond shape); Mm, M. musculus; Rn, R. norvegicus; Sc, S. cerevisiae (red triangle); Sp, S. pombe; Zm, Z. mays; Rc, R. communis; Pt, P. trichocarpa; Vv, V. vinifera; Al, A. lyrta; Mt, M. truncatula; Bd, B. distachyon; Sb, S. bicolor; Sm, S. moellendorffii; Pp, P. patens; Cr, C. reinhardtii and Ol, O. lucimarinus. Phylogeny reconstruction was analyzed by neighbour-joining statistical method based on the JTT matrix-based model. Test of phylogeny was analyzed by the bootstrap method (1,000 replicates). Evolutionary analyses were conducted in MEGA 6.06. (PDF) [file pone.0134709.s002.pdf]

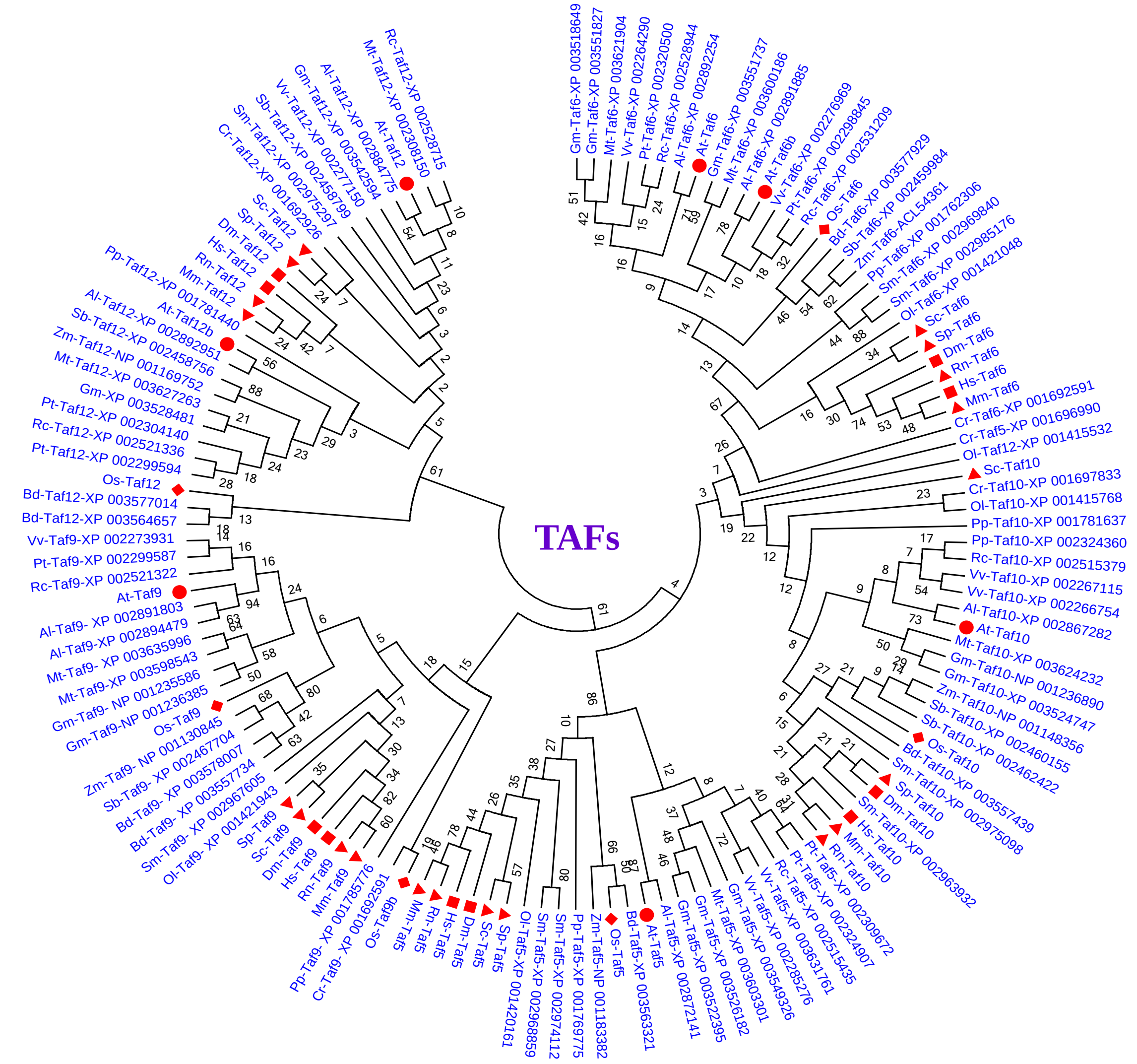

S2 Fig. The phylogenetic relationship of the TAFs group of the SAGA complex.

SAGA complex subunit protein sequences were used from At, *A. thaliana* (red circle); Dm, *D. melongaster*; Hs, *H. sapiens* (red square); Os, *O. sativa* (red diamond shape); Mm, *M. musculus*; Rn, *R. norvegicus;* Sc, *S. cerevisiae* (red triangle); Sp, *S. pombe;* Zm, *Z. mays;* Rc, *R. communis;* Pt, *P. trichocarpa;* Vv, *V. vinifera;* Al, *A. lyrta;* Mt, *M. truncatula;* Bd, *B. distachyon;* Sb, *S. bicolor;* Sm, *S. moellendorffii;* Pp, *P. patens;* Cr, *C. reinhardtii* and Ol, *O. lucimarinus*. Phylogeny reconstruction was analyzed by neighbour-joining statistical method based on the JTT matrix-based model. Test of phylogeny was analyzed by the bootstrap method (1,000 replicates). Evolutionary analyses were conducted in MEGA 6.06.
