# Supplementary material for: Spt-Ada-Gcn5-Acetyltransferase (SAGA) Complex in Plants: Genome Wide Identification, Evolutionary Conservation and Functional Determination
Source: PLoS One. 2015 Aug 11;10(8):e0134709. doi: 10.1371/journal.pone.0134709 (PMC4532415; doi:10.1371/journal.pone.0134709)
Supplement: S3 Fig — Amino acid sequences of domains of the SAGA complex subunits were used from At, A. thaliana; Dm, D. melongaster; Hs, H. sapiens; Os, O. sativa; Mm, M. musculus; Sc, S. cerevisiae. (A) Protein domain of ADAs group; (B) Protein domain of SPTs group; (C) Protein domain of DUBm group; (D) Protein domain of CHD subunit; (E) Protein domain of SGF29 subunit; (F) Protein domain of TRA1 subunit; (G) Protein domain of TAFs group. (PDF) [file pone.0134709.s003.pdf]

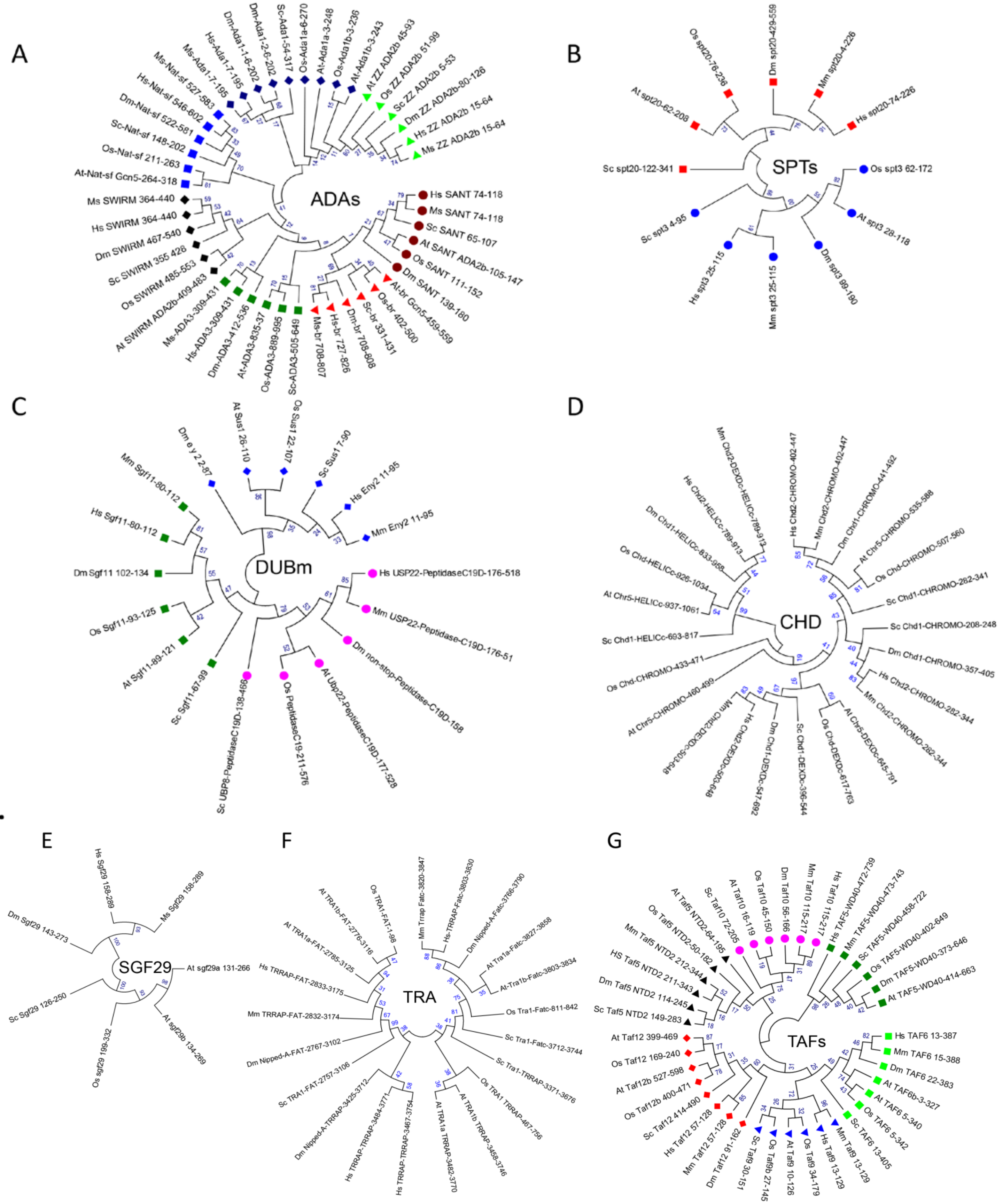

S3 Fig. Molecular phylogenetic analysis of domains of the SAGA complex components. Amino acid sequences of domains of the SAGA complex subunits were used from At, *A. thaliana*; Dm, *D. melongaster*; Hs, *H. sapiens*; Os, *O. sativa*; Mm, *M. musculus*; Sc, *S. cerevisiae.* **(A)** Protein domain of ADAs group; **(B)** Protein domain of SPTs group; **(C)** Protein domain of DUBm group; **(D)** Protein domain of CHD subunit; **(E)** Protein domain of SGF29 subunit; **(F)** Protein domain of TRA1 subunit; **(G)** Protein domain of TAFs group.
